# Supplementary material for: Development and validation of a multimorbidity risk prediction nomogram among Chinese middle-aged and older adults: a retrospective cohort study
Source: BMJ Open. 2023 Nov 8;13(11):e077573. doi: 10.1136/bmjopen-2023-077573 (PMC10632863; doi:10.1136/bmjopen-2023-077573)
Supplement: Supplementary data [file bmjopen-2023-077573supp001.pdf]

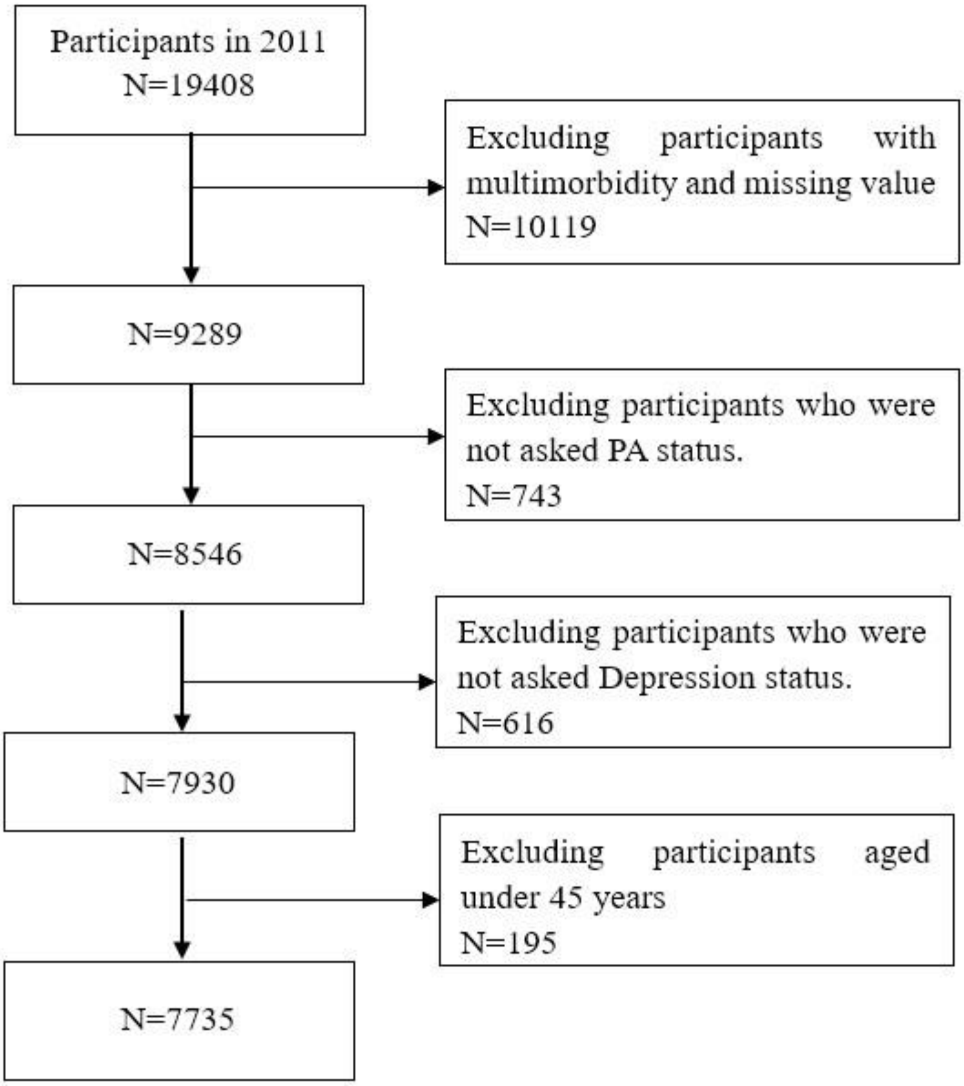


**Figure S1. Flow diagram of Sample selection**

**Table S1 Factors associated with the risk of multimorbidity (univariable cox proportional hazard models, N=7735)**

| **Characteristics** | | ***HR*** | ***95%CI*** | ***P*** |
|---|---|---|---|---|
| **Age (y)** | <50* | | | |
| | 50-69 | 1.25 | 1.13-1.39 | <0.001 |
| | 60-69 | 1.58 | 1.42-1.74 | <0.001 |
| | 70-79 | 1.41 | 1.25-1.60 | <0.001 |
| | >=80 | 1.12 | 0.90-1.41 | 0.311 |
| **Sex** | Male* | | | |
| | Female | 1.16 | 1.08-1.24 | <0.001 |
| **Hukou** | Rural* | | | |
| | Others | 1.03 | 0.95-1.13 | 0.452 |
| **Education** | Primary* | | | |
| | Secondary | 0.83 | 0.77-0.90 | <0.001 |
| **Marial** | Others* | | | |
| | Married | 0.91 | 0.83-0.99 | 0.027 |
| **Chronic Diseases** | No* | | | |
| | Yes | 2.85 | 2.66-3.06 | <0.001 |
| **Sleep (h)** | <7* | | | |
| | >=7 | 0.70 | 0.66-0.75 | <0.001 |
| **Social Activity** | No* | | | |
| | Yes | 0.96 | 0.9-1.02 | 0.215 |
| **Regular PA** | No* | | | |
| | Yes | 0.83 | 0.77-0.88 | <0.001 |
| **Eating** | No* | | | |
| | Yes | 0.97 | 0.88-1.07 | 0.594 |
| **Smoke** | No* | | | |
| | Yes | 1.46 | 1.36-1.56 | <0.001 |
| **Drink** | No* | | | |
| | Yes | 1.46 | 1.36-1.56 | <0.001 |
| **BMI** | | 1.04 | 1.03-1.05 | <0.001 |
| **Depression** | | 1.04 | 1.03-1.05 | <0.001 |
