# Supplementary material for: Development and validation of a multimorbidity risk prediction nomogram among Chinese middle-aged and older adults: a retrospective cohort study
Source: BMJ Open. 2023 Nov 8;13(11):e077573. doi: 10.1136/bmjopen-2023-077573 (PMC10632863; doi:10.1136/bmjopen-2023-077573)
Supplement: Author's manuscript [file bmjopen-2023-077573.draft_revisions.pdf]

BMJ Open

BMJ Open is committed to open peer review. As part of this commitment we make the peer review history of every article we publish publicly available.

When an article is published we post the peer reviewers' comments and the authors' responses online. We also post the versions of the paper that were used during peer review. These are the versions that the peer review comments apply to.

The versions of the paper that follow are the versions that were submitted during the peer review process. They are not the versions of record or the final published versions. They should not be cited or distributed as the published version of this manuscript.

BMJ Open is an open access journal and the full, final, typeset and author-corrected version of record of the manuscript is available on our site with no access controls, subscription charges or pay-per-view fees (http://bmjopen.bmj.com).

If you have any questions on BMJ Open's open peer review process please email info.bmjopen@bmj.com

BMJ Open

# Prevalence, Predictors, and Risk Assessment of Multimorbidity among Middle-aged and Older Adults: A Comprehensive Analysis



| | |
|---|---|
| Journal: | *BMJ Open* |
| Manuscript ID | bmjopen-2023-077573 |
| Article Type: | Original research |
| Date Submitted by the Author: | 13-Jul-2023 |



| | |
|---|---|
| Complete List of Authors: | Zheng, Xiao; Southern Medical University,  Shunde Hospital<br>Xue, Benli; Southern Medical University<br>Xiao, Shujuan; Southern Medical University<br>Li, Xinru; Southern Medical University<br>Chen, Yimin; Southern Medical University<br>Shi, Lei; Southern Medical University<br>Liang, Xiaoyan; The First People's Hospital of Shunde, Department of Health Management<br>Tian, Feng; The First People's Hospital of Shunde, Department of Health Management<br>Zhang, Chichen; Southern Medical University; The First People's Hospital of Shunde, Department of Health Management |



| | |
|---|---|
| Keywords: | Aging, Behavior, Chronic Disease |
| | |


SCHOLARONE™
Manuscripts

*I, the Submitting Author has the right to grant and does grant on behalf of all authors of the Work (as defined in the below author licence), an exclusive licence and/or a non-exclusive licence for contributions from authors who are: i) UK Crown employees; ii) where BMJ has agreed a CC-BY licence shall apply, and/or iii) in accordance with the terms applicable for US Federal Government officers or employees acting as part of their official duties; on a worldwide, perpetual, irrevocable, royalty-free basis to BMJ Publishing Group Ltd ("BMJ") its licensees and where the relevant Journal is co-owned by BMJ to the co-owners of the Journal, to publish the Work in this journal and any other BMJ products and to exploit all rights, as set out in our licence.*

*The Submitting Author accepts and understands that any supply made under these terms is made by BMJ to the Submitting Author unless you are acting as an employee on behalf of your employer or a postgraduate student of an affiliated institution which is paying any applicable article publishing charge ("APC") for Open Access articles. Where the Submitting Author wishes to make the Work available on an Open Access basis (and intends to pay the relevant APC), the terms of reuse of such Open Access shall be governed by a Creative Commons licence – details of these licences and which Creative Commons licence will apply to this Work are set out in our licence referred to above.*

*Other than as permitted in any relevant BMJ Author's Self Archiving Policies, I confirm this Work has not been accepted for publication elsewhere, is not being considered for publication elsewhere and does not duplicate material already published. I confirm all authors consent to publication of this Work and authorise the granting of this licence.*

For peer review only

# Prevalence, Predictors, and Risk Assessment of Multimorbidity among Middle-aged and Older Adults: A Comprehensive Analysis

Xiao Zheng[a], Benli Xue[b], Shujuan Xiao[b], Xinru Li[b], Yimin Chen[b], Lei Shi[b], Xiaoyan Liang[a], Feng Tian[a]*, Chichen Zhang[b,a]*

[a] Department of Health Management, Shunde Hospital, Southern Medical University (The First People's Hospital of Shunde, Foshan), Foshan, China

[b] School of Health Management, Southern Medical University, Guangzhou, China

*Correspondence to:

Chichen Zhang, zhangchichen@sina.com, School of Health Management, Southern Medical University, Guangzhou, China.

Feng Tian, Department of Health Management, Shunde Hospital, Southern Medical University (The First People's Hospital of Shunde, Foshan), Foshan, China

For peer review only



For peer review only - http://bmjopen.bmj.com/site/about/guidelines.xhtml

**Abstract**

**Background:** The aim of this study is to establish a self-simple-to-use nomogram to predict the risk of multimorbidity among middle-aged and older adults. **Methods:** We used data from the Chinese Longitudinal Healthy Longevity Survey, including 7735 samples. Samples' demographic characteristics, modifiable lifestyles, and depression were collected. Cox proportional hazard models and nomogram model were used to estimate the risk factors of multimorbidity.

**Results:** A total of 3576 (46.2%) participants have multimorbidity. The result showed that age (1.01, 95%CI 1.00-1.01), female (1.23, 95%CI 1.10-1.36), chronic disease (2.61, 95%CI 2.40-2.83), sleep time<7h (0.78, 95%CI 0.72-0.84), non-regular PA (0.90, 95%CI 0.83-0.97), drinking (1.26 95%CI 1.15-1.38), smoking (1.38, 95%CI 1.24-1.53), BMI (1.04, 95%CI 1.03-1.05) and higher depression scores (1.02, 95%CI 1.01-1.03) were associated with multimorbidity. The risk scores of multimorbidity were calculated to the PI=(0.3*I[age-45])+(14.3*I[female])+(5.3*I[1-secondary education])+(1.6*I[married])+(56.9*I[chronic disease])+(14.8*I[1-sleep>=7h)+(6.4*I[1-regular PA])+(13.7*I[drink])+(19.1*I[smoke])+(2.2*BMI)+(1.4*depression), where I[ ]denotes the indicator function equal to 1 if the condition in parenthesis is met and 0 otherwise, except age.

**Conclusions:** We developed a user-friendly nomogram model to predict the risk of multimorbidity in middle-aged and older adults. This model incorporates a set of easily accessible and routinely assessed risk factors, facilitating early identification of high-risk individuals and providing personalized preventive and intervention



For peer review only - http://bmjopen.bmj.com/site/about/guidelines.xhtml

measures.

**Keywords**

Multimorbidity, Health risk appraisal, behaviors, Nomogram model, Longitudinal study

**STRENGTHS AND LIMITATIONS OF THIS STUDY**

1. In this study, Age showed a significant correlation with multimorbidity, and the prevalence of multimorbidity was higher in females compared to males. In addition, insufficient sleep, unhealthy lifestyle behaviors, obesity, and depressive symptoms were also associated with multimorbidity.

2. We developed a user-friendly nomogram model to predict the risk of multimorbidity in middle-aged and older adults. The practical implications of the nomogram model provide valuable tools for healthcare professionals to effectively manage multimorbidity risk.

3. The nomogram model was constructed based on a wide range of risk factors, there may still be other unaccounted potential factors influencing the risk of multimorbidity.

## Background

Multimorbidity, commonly defined as the co-occurrence of two or more chronic conditions(1, 2), has emerged as a significant public health concern and poses challenges for healthcare systems. Extensive evidence(3) has shown that



For peer review only - http://bmjopen.bmj.com/site/about/guidelines.xhtml

multimorbidity is associated with an increased risk of mortality(4), reduced quality of life, heightened healthcare utilization, and elevated health costs(5, 6). Thus, the prevention of multimorbidity has become a crucial focus for public health interventions. Consequently, it is essential to understand the prevalence trends of multimorbidity and the contributing factors within populations. This understanding will enable individuals to estimate and modify their personal risk of developing multimorbidity.

Age is recognized as the primary driver of multimorbidity, with a higher prevalence observed among older adults compared to other age groups(7). A systematic review focusing on multimorbidity in the elderly revealed prevalence rates ranging from 55% to 98%, with older age, female gender, and lower socioeconomic status being associated with multimorbidity.(8). Some researchers also estimated the association between sociodemographic, physical characteristics, social networks and multimorbidity(9, 10). The role of lifestyle factors in chronic diseases has been extensively investigated, with numerous reports highlighting a close relationship. Remarkably, a cross-sectional study involving 30,011 participants provided evidence linking poor sleep quality to an increased risk of multimorbidity(11-14). A study found an association between BMI, smoking, and multimorbidity(15). Furthermore, depression has been found to be associated with several common chronic diseases(16, 17). A longitudinal cohort study conducted in China demonstrated bidirectional associations between depression and multimorbidity(18). Despite the established evidence regarding the associations among sociodemographic factors, social



For peer review only - http://bmjopen.bmj.com/site/about/guidelines.xhtml

For peer review only

networks, lifestyle factors, depression, and the risk of developing multimorbidity, individuals still face challenges in accurately estimating their personal risk of developing multimorbidity.

Wider determinants of health (WDHs) encompass various social, economic, political, and environmental factors that influence health outcomes throughout an individual's life. This influential model of health determinants positions constitutional factors such as sex, age, and genetics at its core, followed by overlapping layers representing individual lifestyle factors, and finally the broader determinants(19). While the core attributes remain relatively fixed, the determinants become more modifiable as the layers extend outward. Existing research has identified that individual lifestyle factors significantly contribute to multimorbidity among older adults. Chudasama also found that adopting a healthier lifestyle was associated with longer life expectancy for middle-aged adults, regardless of the presence of multimorbidity.(4). Thus, Furthermore, within the framework of WDHs, individual behaviors represent the first layer that can be modified, particularly through self-health management. Self-management is an effective strategy for preventing and delaying the onset of chronic diseases and multimorbidity(20-22). Accurately understanding one's risk of developing multimorbidity and identifying potential risk factors are critical initial steps in self-management. Therefore, developing a user-friendly tool to help individuals estimating their risk of developing multimorbidity is of utmost importance.

A nomogram is a health risk appraisal model that offers individualized, evidence-



For peer review only - http://bmjopen.bmj.com/site/about/guidelines.xhtml

based, and highly accurate risk estimation (23, 24). It is easy to use and can facilitate self-management-related decision making. It is a user-friendly tool that facilitates decision-making related to self-management. In this study, we have developed the first nomogram for predicting the risk of multimorbidity among middle-aged and older adults.

For peer review only

## Methods

### Study population

We utilized data from the China Health and Retirement Longitudinal Study (CHARLS), a nationally representative survey of Chinese residents aged 45 years and above. The baseline survey was conducted in 2011 using a multistage probability sampling strategy and probability-proportional-to-size (PPS) sampling technique to ensure national representativeness. Follow-up waves were conducted in 2013, 2015, and 2018. Detailed information regarding the purpose, design, sample, and questionnaires used in CHARLS can be found in other studies(6, 25, 26). For this study, participants below the age of 45 and those with missing values in any variables were excluded from the analysis. The selection process is outlined in Additional file-Figure S1.

### Measurements

### Multimorbidity

In this study, multimorbidity was defined as the presence of two or more chronic non-communicable diseases, whether physical or psychological(6, 27). We assessed



For peer review only - http://bmjopen.bmj.com/site/about/guidelines.xhtml

multimorbidity by examining the presence of 14 specific non-communicable diseases. Physical chronic non-communicable diseases encompassed diagnosed conditions such as hypertension, dyslipidemia, diabetes, cancer, chronic lung disease, liver disease, heart disease, stroke, kidney disease, digestive disease, asthma, and arthritis. Psychological chronic non-communicable diseases included diagnosed emotional, nervous, or psychiatric problems, as well as memory-related diseases (all diseases were self-reported and diagnosed chronic conditions). To identify individuals with multimorbidity, we calculated the number of chronic diseases present for each participant.

**The modifiable lifestyles**

This study included four well-known healthy lifestyle factors(4): physical activity (PA), smoking, alcohol consumption and diet behavior. Besides, sleep and social activity were included in this study.

The physical activity questionnaire used in CHARLS closely resembled the short version of the International Physical Activity Questionnaire (IPAQ)(28). However, some differences existed between CHARLS and IPAQ, such as assessing PA for a "usual week" instead of "the last 7 days" and lacking information on sedentariness. Additionally, instead of continuous values, four discrete time durations ("< 30 minutes" "≥30 minutes" "< 4 hours" and "≥ 4 hours") were collected(29). We calculated the median score for each intensity level and summed the number of different intensity levels using the metabolic equivalent (MET) as a reference. The weight of each intensity level was derived from the IPAQ scoring protocol. Detailed



For peer review only - http://bmjopen.bmj.com/site/about/guidelines.xhtml

information on PA and its calculation process can be found in the study by Li(26). The total weekly PA (MET-minutes/week) was calculated by multiplying the frequency, duration, and MET values. According to IPAQ, a minimum total PA of at least 600 MET-minutes/week was defined as regular PA, while <600 MET-minutes/week indicated a lack of regular PA.

Smoking was categorized as No (not current smoker) and Yes (current smoker) at the time of assessment. Alcohol consumption status was divided into 2 groups: No (Did not drink in the past 12 months or drinking frequency is less than weekly) and Yes (others). Regular eating behavior was determined based on the frequency of meals per day, with having three meals on time considered as regular eating.

Based on studies conducted in developed countries, respondents' total sleep duration was classified into five categories: <6 hours, 6 to <7 hours, 7 to <8 hours, 8 to <9 hours, and ≥9 hours(30, 31). According to the Healthy China initiative (2019-2030), the length of night-time sleep ≥ 7h was defined as enough sleep in this study (32). Social activity was categorized as "No" and "Yes" based on engagement in social activities within the past 12 months.

**Demographic characteristics**

Demographic characteristics included age, sex (male and female), marital status (others and married), residency (others and rural), education (primary education and below, secondary education and above), and BMI scores.

**Depression**

Depression was assessed using the Chinese version of the Center for Epidemiological



For peer review only - http://bmjopen.bmj.com/site/about/guidelines.xhtml

Studies Depression scale (CES-D-10)(33). The CES-D-10 contains of 10 items with 4 response options: rare, some days (1–2 days), occasionally (3–4 days) and most of the time (5–7 days)(25). The scales for each of the 10 items were adjusted to 0, 1, 2 and 3, resulting in a CES-D-10 score ranging from 0 to 30, with higher scores indicating more negative feelings during the past week(34, 35).

**Statistical analysis**

The participants were randomly divided into a derivation set and a validation set at a ratio of 7:3. Participant characteristics, such as age and BMI, were summarized as mean ± SD and counts with proportions for categorical features. Cox proportional hazard models were used to estimate the associations between modifiable lifestyles (including physical activity, smoking, alcohol consumption, and diet behavior), depression, and other identified risk factors with the development of multimorbidity in middle-aged and older adults. Hazard ratios (HR) and 95% confidence intervals (95% CI) were reported for the total population. Factors with a significant level of less than 0.05 in the univariable regression model were entered into the multivariable Cox proportional hazard model for adjustment.

A nomogram was developed based on the results of the multivariable cox proportional hazard model in the derivation set. The nomogram assigns risk points to each variable by proportionally converting regression coefficients to a 0-100 point scale. The variable with the highest absolute value of the β coefficient is assigned 100 points. The risk points for other variables are calculated based on the ratio of risk points to the $\beta$ coefficient of the highest variable. A prognostic index (PI) was calculated by



For peer review only - http://bmjopen.bmj.com/site/about/guidelines.xhtml

summing the risk points corresponding to each weighted covariate. The nomogram was validated using the concordance index (C-index) calculated through 1000-fold bootstrap resampling to reduce overfit bias. The developed nomogram was then applied to the validation set. Model performance was further evaluated using a calibration curve, which superimposes both datasets for visual comparison of discrimination. All analyses were performed using R, version 3.0, $P<0.05$ was considered to indicate statistical significance.

For peer review only

## Results

### Baseline characteristics

A total of 7,735 participants were included in this study, with 5,449 individuals in the derivation set and 2,286 in the validation set. The baseline characteristics of the study sample are presented in Table 1. The average age of participants in both datasets was 59.0±9.2 years, and in 2011, 3,726 individuals (48.2%) had at least one chronic disease.

**Table 1 Characteristics of the sample on the baseline-2011**

| Characteristics | *N (%) /S±$\bar{X}$* | | |
|---|---|---|---|
| | **All** | **Derivation Set** | **Validation Set** |
| **Age (y)** | | | |
| <60 | 4387(56.7) | 3039(55.8) | 1348(59.0) |
| ≥60 | 3348(43.3) | 2410(44.2) | 938(41.0) |
| **Sex** | | | |
| Male | 3744(48.4) | 2684(49.3) | 1060(46.4) |
| Female | 3991(51.6) | 2765(50.7) | 1226(53.6) |



For peer review only - http://bmjopen.bmj.com/site/about/guidelines.xhtml

| | | | |
|---|---|---|---|
| **Hukou** | | | |
| Others | 6423(83.0) | 4504(82.7) | 1919(83.9) |
| Rural | 1312(17.0) | 945(17.3) | 367(16.1) |
| **Education** | | | |
| Primary education and below | 5296(68.5) | 3720(68.3) | 1576(68.9) |
| Secondary education and above | 2439(31.5) | 1729(31.7) | 710(31.1) |
| **Martial** | | | |
| Others | 1304(16.9) | 910(16.7) | 394(17.2) |
| Married | 6431(83.1) | 4539(83.3) | 1892(82.8) |
| **Chronic Disease** | | | |
| No | 4009(51.8) | 2817(51.7) | 1192(52.1) |
| Yes | 3726(48.2) | 2632(48.3) | 1094(47.9) |
| **Sleep (h)** | | | |
| <7 | 3803(49.2) | 2701(49.6) | 1102(48.2) |
| ≥7 | 3932(50.8) | 2748(50.4) | 1184(51.8) |
| **Social Activity** | | | |
| No | 3864(50.0) | 2747(50.4) | 1117(48.9) |
| Yes | 3871(50.0) | 2702(49.6) | 1169(51.1) |
| **Regular PA** | | | |
| No | 3422(44.2) | 2407(44.2) | 1015(44.4) |
| Yes | 4313(55.8) | 3042(55.8) | 1271(55.6) |
| **Regular Eating** | | | |
| No | 1009(13.0) | 707(13.0) | 302(13.2) |
| Yes | 6726(87.0) | 4742(87.0) | 1984(86.8) |
| **Smoke** | | | |
| No | 3379(43.7) | 2390(43.9) | 989(43.3) |
| Yes | 4356(56.3) | 3059(56.1) | 1297(56.7) |
| **Drink** | | | |



For peer review only

| | | | |
|---|---|---|---|
| No | 3354(43.4) | 2387(43.8) | 967(42.3) |
| Yes | 4381(56.6) | 3062(56.2) | 1319(57.7) |
| BMI | 23.32±3.69 | 23.31±3.72 | 23.35±3.62 |
| **Depression** | 9.13±4.57 | 9.10±4.56 | 9.21±4.60 |

## Prevalence of multimorbidity

In 2018, a total of 3,576 participants (46.2%) were found to have multimorbidity. Among these individuals, the prevalence of multimorbidity was higher among older adults compared to those under the age of 60 (51.0% vs. 42.6%). Additionally, females exhibited a higher prevalence of multimorbidity compared to males (48.5% vs. 43.8%). Moreover, married individuals (45.7% vs. 48.6%) and those with higher education (42.1% vs. 48.1%) had a lower prevalence of multimorbidity compared to others (Figure 1).

**Figure 1. Prevalence of multimorbidity in the population**

## Predictor factors of multimorbidity

The results of the univariable Cox proportional hazard model for the entire dataset (Additional file-Table S1) indicated that several factors were significantly associated with an increased risk of multimorbidity. These factors included older age (HR 1.01, 95% CI 1.01-1.01), female (HR 1.16, 95% CI 1.08-1.24), presence of chronic diseases (HR 2.85, 95% CI 2.66-3.06), and higher depression scores (HR 1.04, 95% CI 1.03-1.05). Higher education (HR 0.83, 95%CI 0.77-0.89), being married (HR 0.90, 95%CI 0.83-0.98), higher BMI scores (HR 1.04, 95%CI 1.03-1.05), smoking (HR 1.46, 95%CI 1.36-1.56) and drinking (1.46, 95%CI 1.36-1.56), have more than 7 hours of



For peer review only - http://bmjopen.bmj.com/site/about/guidelines.xhtml

sleep (HR 0.70, 95%CI 0.66-0.75), engaging in regular PA (HR 0.83, 95%CI 0.77-0.88) were associated with a decreased risk of multimorbidity.

Eleven meaningful variables were included in the multivariable cox proportional hazard models. The result showed that age (HR 1.01, 95%CI 1.00-1.01), sex (HR 1.23, 95%CI 1.10-1.36), chronic disease (HR 2.61, 95%CI 2.40-2.83), sleep time (HR 0.78, 95%CI 0.72-0.84), regular PA (HR 0.90, 95%CI 0.83-0.97), drink (HR 1.26 95%CI 1.15-1.38), smoke (HR 1.38, 95%CI 1.24-1.53), BMI (HR 1.04, 95%CI 1.03-1.05) and depression scores (HR 1.02, 95%CI 1.01-1.03) were associated with multimorbidity in derivation set (Table 2).

**Table 2 Factors associated with the risk of multimorbidity (multivariable cox proportional hazard model)**

| **Factors** | **Derivation Set** | | | **Validation Set** | | |
|---|---|---|---|---|---|---|
| | *HR* | *95%CI* | *P* | *HR* | *95%CI* | *P* |
| Age | 1.01 | 1.00-1.01 | 0.017 | 1.01 | 1.00-1.01 | 0.144 |
| Sex (Male) | | | | | | |
| Female | 1.23 | 1.10-1.36 | <0.001 | 1.43 | 1.22-1.67 | <0.001 |
| Education (Primary education) | | | | | | |
| Secondary education | 0.91 | 0.83-1.00 | 0.061 | 0.99 | 0.86-1.15 | 0.936 |
| Marital (Others) | | | | | | |
| Married | 1.03 | 0.92-1.15 | 0.639 | 0.98 | 0.83-1.16 | 0.848 |
| Chronic disease (No) | | | | | | |
| Yes | 2.61 | 2.40-2.83 | <0.001 | 2.72 | 2.39-3.09 | <0.001 |
| Sleep (<7h) | | | | | | |
| >=7h | 0.78 | 0.72-0.84 | <0.001 | 0.74 | 0.65-0.83 | <0.001 |
| Regular PA (No) | | | | | | |
| Yes | 0.90 | 0.83-0.97 | 0.001 | 0.89 | 0.79-1.01 | 0.072 |



For peer review only - http://bmjopen.bmj.com/site/about/guidelines.xhtml

| | | | | | | |
|---|---|---|---|---|---|---|
| Drink (No) | | | | | | |
| Yes | 1.26 | 1.15-1.38 | <0.001 | 1.35 | 1.17-1.56 | <0.001 |
| Smoke (No) | | | | | | |
| Yes | 1.38 | 1.24-1.53 | <0.001 | 1.57 | 1.34-1.84 | <0.001 |
| BMI | 1.04 | 1.03-1.05 | <0.001 | 1.02 | 1.01-1.04 | 0.001 |
| Depression | 1.02 | 1.01-1.03 | <0.001 | 1.02 | 1.01-1.04 | 0.001 |

**Development and validation of an multimorbidity -predicting nomogram**

The PI was calculated based on the hazard ratio associated with the identified risk factors for multimorbidity. The nomogram was constructed using these results, with the BMI variable assigned a total scale of 100 and a range of 5 to 50. The risk score for BMI was determined to be 2.2. The risk scores for the other risk factors of multimorbidity were calculated proportionally to the β coefficient of BMI. So the PI=(0.3*I[age-45])+(14.3*I[female])+(5.3*I[1-secondary education])+(1.6*I[married])+(56.9*I[chronic disease])+(14.8*I[1-sleep>=7h)+(6.4*I[1-regular PA])+(13.7*I[drink])+(19.1*I[smoke])+(2.2*BMI)+(1.4*depression), where I[ ]denotes the indicator function equal to 1 if the condition in parenthesis is met and 0 otherwise, except age. Additional details regarding individual prognostic scores for each risk factor can be found in Additional file-Table S2. Based upon these findings, a nomogram was configured (Figure 2).

**Figure 2. The nomogram for multimorbidity risk prediction. Note: Draw a line perpendicular from the corresponding axis of each risk factor until it reaches the line labeled "POINTS". Sum up the number of points for all risk factors then draw a line descending from the labeled**



For peer review only - http://bmjopen.bmj.com/site/about/guidelines.xhtml

**"TOTAL POINTS" until it intercepts each of the survival axes to determine 2-, 4-, and 7-year survival probabilities, multimorbidity probability=1- survival probability.**

The resulting nomogram was internally validated using the bootstrap validation method, and it demonstrated good accuracy in estimating the risk of multimorbidity, with a bootstrap-corrected C-index of 0.71 in the derivation set. Calibration plots also indicated good agreement between the risk estimation by the nomogram and the diagnosis of doctors, as depicted in Figure 2. When the estimates from the derivation set were applied to the validation set, a similar bootstrap-corrected C-index of 0.71 was obtained, along with a well-calibrated risk estimation curve (Figure 3).

**Figure 3. The predictive performance of the nomogram in estimating the risk of multimorbidity**

## Discussion

In this study, we observed a prevalence of multimorbidity of 46.2% among middle-aged and older adults. The prevalence of multimorbidity was higher among individuals with one chronic disease (62.8%) compared to those without chronic diseases (30.8%) at baseline. This result is consistent with the probability range of multimorbidity reported in a systematic study of the elderly(8). Although there were some differences in the estimation of multimorbidity compared to other studies, such as the number of included chronic diseases(36, 37), and the methods used to collect information(38). Our results confirmed the significant impact of multimorbidity on



For peer review only - http://bmjopen.bmj.com/site/about/guidelines.xhtml

middle-aged and older adults, particularly among those with one chronic disease.

Our findings provide sufficient evidence for the association between older age and multimorbidity, which is consistent with similar trends observed in countries such as Singapore(39), Ireland, and Scotland(27). The prevalence of multimorbidity among females is higher than males. Additionally, we found a higher prevalence of multimorbidity among females compared to males, which is supported by studies from various countries indicating that older men have a lower risk of multimorbidity than their female counterparts(27, 38). However, a study by Lian found that the onset of multimorbidity occurs at an earlier age in males than in females(39). This is also a new idea to understand the sex-differ in multimorbidity, but our study only included middle-aged and older adults aged over 45 years, we cannot evaluate this result of Lian. Future research could explore the prevalence and different combinations of chronic conditions in people with multimorbidity across various age and sex groups.

Existing studies have established a link between short sleep duration and multimorbidity. Experimental evidence confirms the deleterious effects of sleep deprivation on endocrine, immune, neurovitality and inflammatory pathways(40). For instance, Maria Ruiz-Castell found an association between short sleep duration and the number of chronic conditions(41). Helbig also observed a significant positive relationship between short sleep duration and multimorbidity among women(42). High-risk lifestyles, such as smoking, excessive alcohol consumption, poor diet, physical inactivity, and unhealthy body shape, have also been confirmed as contributing factors to multimorbidity(43). Smoking and excessive alcohol



For peer review only - http://bmjopen.bmj.com/site/about/guidelines.xhtml

consumption remain leading risk factors for early death and disability globally(44, 45). In our study, we found that smoking and drinking increased the risk of multimorbidity, with the highest risk index among all unhealthy behaviors. The expansion of tobacco and alcohol control measures remains a significant public health priority worldwide. Mika conducted an observational study using data from two Finnish cohort studies comprising 614,014 adults, and the results showed that obesity is a significant factor in multimorbidity(46). Similarly, our study found that higher BMI was associated with an increased risk of multimorbidity among middle-aged and older adults. There exists a bidirectional association between depression and multimorbidity(16, 47). Depression increases the risk of multimorbidity, while having multimorbidity also raises the risk of depression. Our study found the risk of multimorbidity for middle-aged and older adults with higher depression scores.

Based on our results, Based on our results, we developed a user-friendly nomogram model for predicting the risk of multimorbidity. Our nomogram model incorporates an extensive set of easily obtainable risk factors that are routinely assessed by individuals. Additionally, these results can guide preventive therapies, such as self-health-management interventions, for individuals with one chronic disease.

One of the most appealing aspects of our nomogram model is its home applicability and ease of use by individuals. For example, a 50-year-old married male with primary education, one chronic disease, a history of smoking and excessive alcohol consumption, irregular physical activity, 8 hours of sleep, a depression score of 16, and a BMI of 24.9 would have a total risk score of 219.3 points. This corresponds to a



For peer review only - http://bmjopen.bmj.com/site/about/guidelines.xhtml

2-, 4-, and 7-year probability of multimorbidity of 32%, 71%, and <10%, respectively. The nomogram is relatively straightforward to understand and can be obtained quickly using a simple intake form (Additional file -Table S2). Based on the calculated results, individuals can develop self-management strategies to reduce their risk of multimorbidity.

**Limitation**

Our study had several limitations. First, the model was constructed based on behavioral and household-level risk factors, limiting its applicability to clinical prediction. Second, the model was only evaluated in the same sample for internal validation and requires further validation in different populations. Additionally, although our model was constructed based on a wide range of risk factors, there may still be other unaccounted potential factors influencing the risk of multimorbidity. Therefore, further research is warranted to refine and optimize predictive models for assessing multimorbidity risk.

## Conclusions

This study provides a comprehensive analysis of multimorbidity among middle-aged and older adults, focusing on its prevalence, predictors, and risk assessment. This study confirms the severity of multimorbidity among middle-aged and older adults, particularly among those who already have one chronic disease. The study also identified several predictive factors associated with multimorbidity. Age showed a significant correlation with multimorbidity, and the prevalence of multimorbidity was



For peer review only - http://bmjopen.bmj.com/site/about/guidelines.xhtml

For peer review only

higher in females compared to males. In addition, insufficient sleep, unhealthy lifestyle behaviors (such as smoking and alcohol consumption), obesity, and depressive symptoms were also associated with multimorbidity. Based on these findings, we developed a user-friendly nomogram model to predict the risk of multimorbidity in middle-aged and older adults. The findings reveal a high prevalence of multimorbidity in this population and shed light on the importance of targeted interventions and healthcare planning. The practical implications of the nomogram model provide valuable tools for healthcare professionals to effectively manage multimorbidity risk. In summary, this study contributes to the understanding of multimorbidity and offers insights for reducing the burden of chronic diseases in middle-aged and older adults.

## Ethics statements

### Patient consent for publication

Not applicable.

### Ethics approval

The Biomedical Ethics Review Committee of Peking University approved CHARLS, and all participants were required to provide written informed consent. The ethical approval number was IRB00001052-11015.

## Supplementary materials

Additional file.



For peer review only - http://bmjopen.bmj.com/site/about/guidelines.xhtml

## Footnotes

**Contributors:** ZCC and TF conceived the study. ZX was a major contributor in writing the manuscript. XBL, XSJ, LXR, analyzed and interpreted the patient data. CYM SL and LXY edited and contributed content to the final draft. All authors read and approved the final manuscript.

**Funding:** This study was funded by the National Nature Science Foundation of China (No. 72274091), Guangdong Basic and Applied Basic Research Foundation (No.2022A1515110295, No.2022A1515011591), China Postdoctoral Science Foundation (No. 2022M721539).

**Competing interests:** The authors declare that they have no competing interests.

**Patient and public involvement:** Patients and/or the public were not involved in the design, or conduct, or reporting, or dissemination plans of this research.

**Availability of data and materials:** The datasets analysed during the current study are available from the corresponding author on reasonable request.

## References

1. Fortin M, Bravo G, Hudon C, Vanasse A, Lapointe L. Prevalence of multimorbidity among adults seen in family practice. Ann Fam Med. 2005;3(3):223-8.

2. Jiao DD, Watanabe K, Sawada Y, Tanaka E, Watanabe T, Tomisaki E, et al. Multimorbidity and functional limitation: the role of social relationships. Arch



For peer review only - http://bmjopen.bmj.com/site/about/guidelines.xhtml

Gerontol Geriat. 2021;92.

3. Nicholson K, Griffith LE, Sohel N, Raina P. Examining early and late onset of multimorbidity in the Canadian Longitudinal Study on Aging. J Am Geriatr Soc. 2021.

4. Chudasama YV, Khunti K, Gillies CL, Dhalwani NN, Davies MJ, Yates T, et al. Healthy lifestyle and life expectancy in people with multimorbidity in the UK Biobank: A longitudinal cohort study. PLoS Med. 2020;17(9):e1003332.

5. Ding D, Lawson KD, Kolbe-Alexander TL, Finkelstein EA, Katzmarzyk PT, van Mechelen W, et al. The economic burden of physical inactivity: a global analysis of major non-communicable diseases. Lancet. 2016;388(10051):1311-24.

6. Zhao Y, Atun R, Oldenburg B, McPake B, Tang S, Mercer SW, et al. Physical multimorbidity, health service use, and catastrophic health expenditure by socioeconomic groups in China: an analysis of population-based panel data. The Lancet Global Health. 2020;8(6):e840-e9.

7. Head A, Fleming K, Kypridemos C, Pearson-Stuttard J, O'Flaherty M. Multimorbidity: the case for prevention. J Epidemiol Community Health. 2021;75(3):242-4.

8. Marengoni A, Angleman S, Melis R, Mangialasche F, Karp A, Garmen A, et al. Aging with multimorbidity: A systematic review of the literature. Ageing Res Rev. 2011;10(4):430-9.

9. Bezerra de Souza DL, Oliveras-Fabregas A, Espelt A, Bosque-Prous M, de Camargo Cancela M, Teixido-Compano E, et al. Multimorbidity and its associated



For peer review only - http://bmjopen.bmj.com/site/about/guidelines.xhtml

factors among adults aged 50 and over: A cross-sectional study in 17 European countries. PLoS One. 2021;16(2):e0246623.

10. Olaya B, Domenech-Abella J, Moneta MV, Lara E, Caballero FF, Rico-Uribed LA, et al. All-cause mortality and multimorbidity in older adults: The role of social support and loneliness. Exp Gerontol. 2017;99:120-6.

11. Bertuol C, Tozetto WR, Streb AR, Del Duca GF. Combined relationship of physical inactivity and sedentary behaviour with the prevalence of noncommunicable chronic diseases: data from 52,675 Brazilian adults and elderly. Eur J Sport Sci. 2021.

12. Lindstrom J, Uusitupa M. Lifestyle intervention, diabetes, and cardiovascular disease. Lancet. 2008;371(9626):1731-3.

13. Elwood P, Galante J, Pickering J, Palmer S, Bayer A, Ben-Shlomo Y, et al. Healthy Lifestyles Reduce the Incidence of Chronic Diseases and Dementia: Evidence from the Caerphilly Cohort Study. Plos One. 2013;8(12).

14. Hu FB, Liu Y, Willett WC. Preventing chronic diseases by promoting healthy diet and lifestyle: public policy implications for China. Obes Rev. 2011;12(7):552-9.

15. Fortin M, Haggerty J, Almirall J, Bouhali T, Sasseville M, Lemieux M. Lifestyle factors and multimorbidity: a cross sectional study. BMC Public Health. 2014;14:686.

16. Birk JL, Kronish IM, Moise N, Falzon L, Yoon S, Davidson KW. Depression and Multimorbidity: Considering Temporal Characteristics of the Associations Between Depression and Multiple Chronic Diseases. Health Psychology. 2019;38(9):802-11.

17. Meng L, Chen DM, Yang Y, Zheng Y, Hui RT. Depression increases the risk of hypertension incidence: a meta-analysis of prospective cohort studies. J Hypertens.

For peer review only



For peer review only - http://bmjopen.bmj.com/site/about/guidelines.xhtml

2012;30(5):842-51.

18. Qiao YN, Liu SY, Zhang YX, Wu Y, Shen YP, Ke CF. Bidirectional association between depression and multimorbidity in middle-aged and elderly Chinese adults: a longitudinal cohort study. Aging & Mental Health. 2021.

19. Bambra C, Gibson M, Sowden A, Wright K, Whitehead M, Petticrew M. Tackling the wider social determinants of health and health inequalities: evidence from systematic reviews. J Epidemiol Commun H. 2010;64(4):284-91.

20. Chodosh J, Morton SC, Mojica W, Maglione M, Suttorp MJ, Hilton L, et al. Meta-analysis: Chronic disease self-management programs for older adults. Ann Intern Med. 2005;143(6):427-38.

21. Warsi A, Wang PS, LaValley MP, Avorn J, Solomon DH. Self-management education programs in chronic disease - A systematic review and methodological critique of the literature. Arch Intern Med. 2004;164(15):1641-9.

22. Li WY, Chiu FC, Zeng JK, Li YW, Huang SH, Yeh HC, et al. Mobile Health App With Social Media to Support Self-Management for Patients With Chronic Kidney Disease: Prospective Randomized Controlled Study. Journal of Medical Internet Research. 2020;22(12).

23. Lei Z, Li J, Wu D, Xia Y, Wang Q, Si A, et al. Nomogram for Preoperative Estimation of Microvascular Invasion Risk in Hepatitis B Virus-Related Hepatocellular Carcinoma Within the Milan Criteria. JAMA Surg. 2016;151(4):356-63.

24. B OH, Gransar H, Callister T, Shaw LJ, Schulman-Marcus J, Stuijfzand WJ, et al.



For peer review only - http://bmjopen.bmj.com/site/about/guidelines.xhtml

Development and Validation of a Simple-to-Use Nomogram for Predicting 5-, 10-, and 15-Year Survival in Asymptomatic Adults Undergoing Coronary Artery Calcium Scoring. JACC Cardiovasc Imaging. 2018;11(3):450-8.

25. Jing R, Xu T, Rong H, Lai X, Fang H. Longitudinal Association Between Sleep Duration and Depressive Symptoms in Chinese Elderly. Nat Sci Sleep. 2020;12:737-47.

26. Li X, Zhang W, Zhang W, Tao K, Ni W, Wang K, et al. Level of physical activity among middle-aged and older Chinese people: evidence from the China health and retirement longitudinal study. BMC Public Health. 2020;20(1):1682.

27. Barnett K, Mercer SW, Norbury M, Watt G, Wyke S, Guthrie B. Epidemiology of multimorbidity and implications for health care, research, and medical education: a cross-sectional study. Lancet. 2012;380(9836):37-43.

28. Craig CL, Marshall AL, Sjostrom M, Bauman AE, Booth ML, Ainsworth BE, et al. International physical activity questionnaire: 12-country reliability and validity. Med Sci Sports Exerc. 2003;35(8):1381-95.

29. Zhao Y, Hu Y, Smith JP, Strauss J, Yang G. Cohort profile: the China Health and Retirement Longitudinal Study (CHARLS). Int J Epidemiol. 2014;43(1):61-8.

30. Yang L, Yang H, He M, Pan A, Li X, Min X, et al. Longer Sleep Duration and Midday Napping Are Associated with a Higher Risk of CHD Incidence in Middle-Aged and Older Chinese: the Dongfeng-Tongji Cohort Study. Sleep. 2016;39(3):645-52.

31. Paudel M, Taylor BC, Ancoli-Israel S, Blackwell T, Maglione JE, Stone K, et al.



For peer review only - http://bmjopen.bmj.com/site/about/guidelines.xhtml

For peer review only

Sleep Disturbances and Risk of Depression in Older Men. Sleep. 2013;36(7):1033-40.

32. China TPsRo. Healthy China initiative (2019-2030) 2019 [Available from: http://www.gov.cn/xinwen/2019-07/15/content_5409694.htm.

33. Jin Y, Luo Y, He P. Hypertension, socioeconomic status and depressive symptoms in Chinese middle-aged and older adults: Findings from the China health and retirement longitudinal study. J Affect Disord. 2019;252:237-44.

34. Wang RX, Chen Z, Zhou YJ, Shen LN, Zhang Z, Wu X. Melancholy or mahjong? Diversity, frequency, type, and rural-urban divide of social participation and depression in middle- and old-aged Chinese: A fixed-effects analysis. Soc Sci Med. 2019;238.

35. Chen Z, Ying J, Ingles J, Zhang D, Rajbhandari-Thapa J, Wang R, et al. Gender differential impact of bereavement on health outcomes: evidence from the China Health and Retirement Longitudinal Study, 2011-2015. BMC Psychiatry. 2020;20(1):514.

36. Diederichs C, Berger K, Bartels DB. The Measurement of Multiple Chronic Diseases-A Systematic Review on Existing Multimorbidity Indices. J Gerontol a-Biol. 2011;66(3):301-11.

37. Guisado-Clavero M, Roso-Llorach A, Lopez-Jimenez T, Pons-Vigues M, Foguet-Boreu Q, Munoz MA, et al. Multimorbidity patterns in the elderly: a prospective cohort study with cluster analysis. Bmc Geriatrics. 2018;18.

38. Hewitt J, McCormack C, Tay HS, Greig M, Law J, Tay A, et al. Prevalence of multimorbidity and its association with outcomes in older emergency general surgical



For peer review only - http://bmjopen.bmj.com/site/about/guidelines.xhtml

patients: an observational study. Bmj Open. 2016;6(3).

39. Low LL, Kwan YH, Ko MSM, Yeam CT, Lee VSY, Tan WB, et al. Epidemiologic Characteristics of Multimorbidity and Sociodemographic Factors Associated With Multimorbidity in a Rapidly Aging Asian Country. JAMA Netw Open. 2019;2(11):e1915245.

40. Ferrie JE, Kivimaki M, Akbaraly TN, Singh-Manoux A, Miller MA, Gimeno D, et al. Associations Between Change in Sleep Duration and Inflammation: Findings on C-reactive Protein and Interleukin 6 in the Whitehall II Study. American Journal of Epidemiology. 2013;178(6):956-61.

41. Ruiz-Castell M, Makovski TT, Bocquet V, Stranges S. Sleep duration and multimorbidity in Luxembourg: results from the European Health Examination Survey in Luxembourg, 2013-2015. BMJ Open. 2019;9(8):e026942.

42. Helbig AK, Stockl D, Heier M, Thorand B, Schulz H, Peters A, et al. Relationship between sleep disturbances and multimorbidity among community-dwelling men and women aged 65-93 years: results from the KORA Age Study. Sleep Med. 2017;33:151-9.

43. Han Y, Hu Y, Yu C, Guo Y, Pei P, Yang L, et al. Lifestyle, cardiometabolic disease, and multimorbidity in a prospective Chinese study. Eur Heart J. 2021;42(34):3374-84.

44. Beaglehole R, Bates C, Youdan B, Bonita R. Nicotine without smoke: fighting the tobacco epidemic with harm reduction. Lancet. 2019;394(10200):718-20.

45. Mohammad KA, Salomon JA. Smoking prevalence and attributable disease

For peer review only



For peer review only - http://bmjopen.bmj.com/site/about/guidelines.xhtml

burden in 195 countries and territories, 1990-2015: a systematic analysis from the Global Burden of Disease Study 2015 (vol 389, pg 1885, 2015). Lancet. 2017;390(10103):1644-.

46. Kivimaki M, Strandberg T, Pentti J, Nyberg ST, Frank P, Jokela M, et al. Body-mass index and risk of obesity-related complex multimorbidity: an observational multicohort study. Lancet Diabetes Endocrinol. 2022;10(4):253-63.

47. Huang CQ, Dong BR, Lu ZC, Yue JR, Liu QX. Chronic diseases and risk for depression in old age: A meta-analysis of published literature. Ageing Res Rev. 2010;9(2):131-41.

For peer review only



For peer review only - http://bmjopen.bmj.com/site/about/guidelines.xhtml

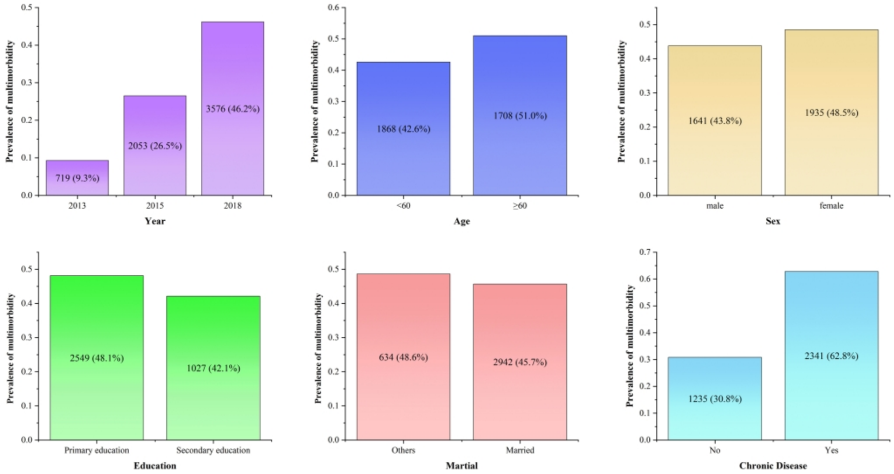


Figure 1. Prevalence of multimorbidity in the population.

338x179mm (300 x 300 DPI)

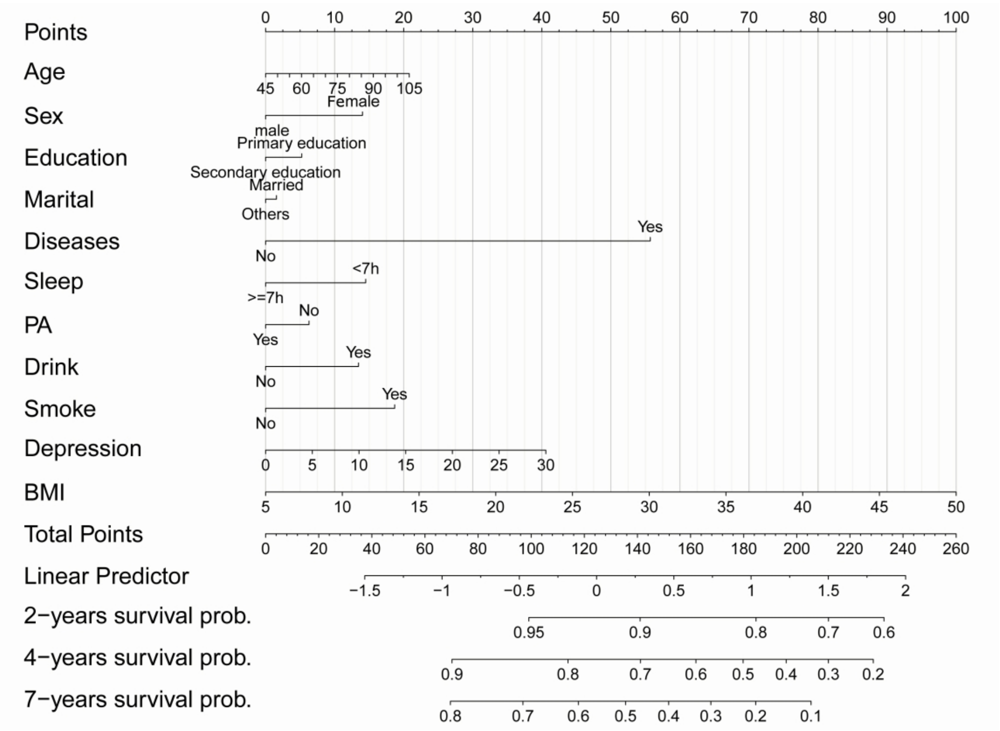


Figure 2. The nomogram for multimorbidity risk prediction. Note: Draw a line perpendicular from the corresponding axis of each risk factor until it reaches the line labeled “POINTS”. Sum up the number of points for all risk factors then draw a line descending from the labeled “TOTAL POINTS” until it intercepts each of the survival axes to determine 2-, 4-, and 7-year survival probabilities, multimorbidity probability=1- survival probability.

286x208mm (300 x 300 DPI)

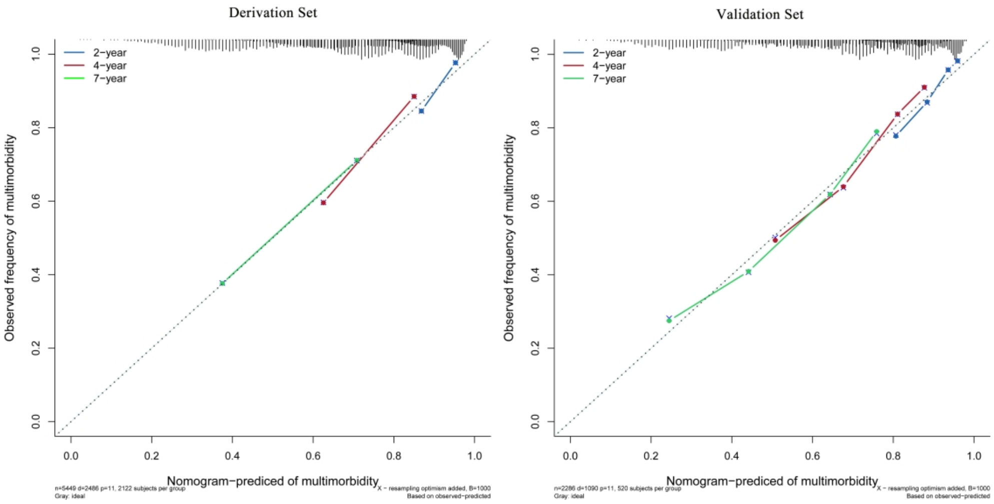


Figure 3. The predictive performance of the nomogram in estimating the risk of multimorbidity.

394x209mm (300 x 300 DPI)

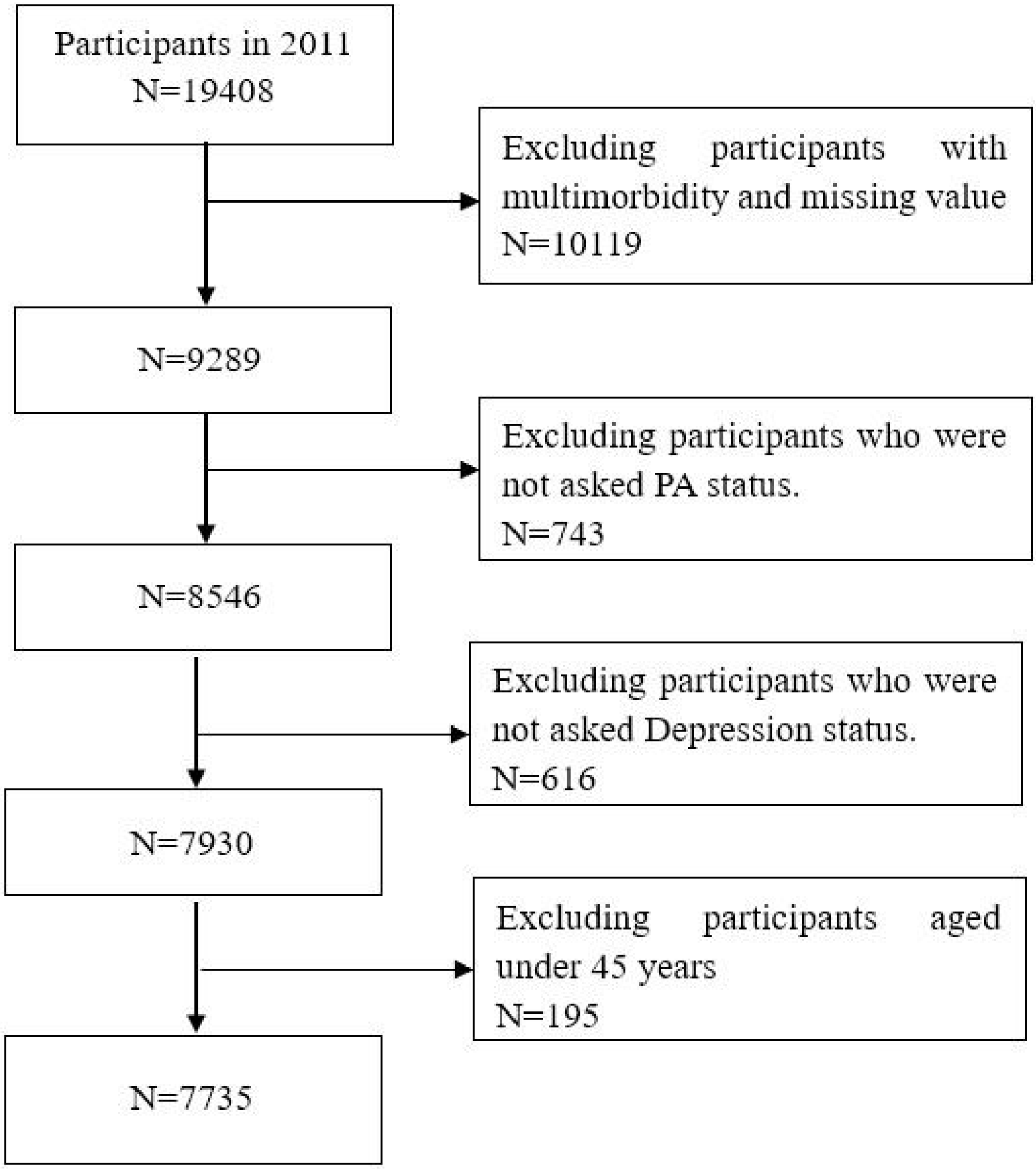


**Figure S1. Flow diagram of Sample selection**

**Table S1 Factors associated with the risk of multimorbidity (univariable cox proportional hazard models, N=7927)**

| **Characteristics** | | ***HR*** | ***95%CI*** | ***P*** |
|---|---|---|---|---|
| **Age (y)** | | 1.01 | 1.01-1.01 | <0.001 |
| **Sex** | Male* | | | |
| | Female | 1.16 | 1.08-1.24 | <0.001 |
| **Hukou** | Rural* | | | |
| | Others | 1.03 | 0.95-1.13 | 0.514 |
| **Education** | Primary* | | | |
| | Secondary | 0.83 | 0.77-0.90 | <0.001 |
| **Marial** | Others* | | | |
| | Married | 0.91 | 0.83-0.99 | 0.027 |
| **Chronic Diseases** | No* | | | |
| | Yes | 2.85 | 2.66-3.06 | <0.001 |
| **Sleep (h)** | <7* | | | |
| | >=7 | 0.70 | 0.66-0.75 | <0.001 |
| **Social Activity** | No* | | | |
| | Yes | 0.96 | 0.9-1.02 | 0.215 |
| **Regular PA** | No* | | | |
| | Yes | 0.83 | 0.77-0.88 | <0.001 |
| **Eating** | No* | | | |
| | Yes | 0.97 | 0.88-1.07 | 0.594 |
| **Smoke** | No* | | | |
| | Yes | 1.46 | 1.361.56 | <0.001 |
| **Drink** | No* | | | |
| | Yes | 1.46 | 1.361.56 | <0.001 |
| **BMI** | | 1.04 | 1.03-1.05 | <0.001 |
| **Depression** | | 1.04 | 1.03-1.05 | <0.001 |

For peer review only

For peer review only

**Table S2 Index for predicting mortality risk**

| Risk Factor | | Risk Score |
|---|---|---|
| 1. | **Age (years):** | |
| | 45-55 | 0-3.0 |
| | 56-65 | 3.3-6.0 |
| | 66-75 | 6.3-9.0 |
| | 76-85 | 9.3-12.0 |
| | 86-95 | 12.3-15.0 |
| | 96-105 | 15.3-18.0 |
| 2. | **Sex:** | |
| | Male | 0 |
| | Female | 14.3 |
| 3. | **Education** | |
| | Primary education | 5.3 |
| | Secondary education | 0 |
| 4. | **Marital** | |
| | Others | 0 |
| | Married | 1.6 |
| 5. | **Do you have chronic disease** | |
| | No | 0 |
| | Yes | 56.9 |
| 6. | **Sleep time** | |
| | <7h | 14.8 |
| | >=7h | 0 |
| 7. | **Do you have regular PA** | |
| | No | 6.4 |

| | | |
|---|---|---|
| | Yes | 0 |
| 8. | **Are you a current drinker** | |
| | No | 0 |
| | Yes | 13.7 |
| 9. | **Are you a current smoker** | |
| | No | 0 |
| | Yes | 19.1 |
| 10. | **BMI score** | 0-100 |
| | **Depression score** | |
| | 0-30 | 0-42.0 |
| | **Total Risk Score:** | |

For peer review only

BMJ Open

# Development and Validation of a multivariable prediction model for Multimorbidity Risk in middle-aged and older adults: A Nomogram approach



| | |
|---|---|
| Journal: | *BMJ Open* |
| Manuscript ID | bmjopen-2023-077573.R1 |
| Article Type: | Original research |
| Date Submitted by the Author: | 19-Sep-2023 |
| Complete List of Authors: | Zheng, Xiao; Shunde Hospital, Southern Medical University (The First People's Hospital of Shunde, Foshan), Department of Health Management<br>Xue, Benli; Southern Medical University<br>Xiao, Shujuan; Southern Medical University<br>Li, Xinru; Southern Medical University<br>Chen, Yimin; Southern Medical University<br>Shi, Lei; Southern Medical University<br>Liang, Xiaoyan; Shunde Hospital, Southern Medical University (The First People's Hospital of Shunde, Foshan), Department of Health Management<br>Tian, Feng; Shunde Hospital, Southern Medical University (The First People's Hospital of Shunde, Foshan), Department of Health Management<br>Zhang, Chichen; Southern Medical University |
| <b>Primary Subject Heading</b>: | Health services research |
| Secondary Subject Heading: | General practice / Family practice |
| Keywords: | Aging, Behavior, Chronic Disease |
| | |



SCHOLARONE™
Manuscripts

*I, the Submitting Author has the right to grant and does grant on behalf of all authors of the Work (as defined in the below author licence), an exclusive licence and/or a non-exclusive licence for contributions from authors who are: i) UK Crown employees; ii) where BMJ has agreed a CC-BY licence shall apply, and/or iii) in accordance with the terms applicable for US Federal Government officers or employees acting as part of their official duties; on a worldwide, perpetual, irrevocable, royalty-free basis to BMJ Publishing Group Ltd ("BMJ") its licensees and where the relevant Journal is co-owned by BMJ to the co-owners of the Journal, to publish the Work in this journal and any other BMJ products and to exploit all rights, as set out in our licence.*

*The Submitting Author accepts and understands that any supply made under these terms is made by BMJ to the Submitting Author unless you are acting as an employee on behalf of your employer or a postgraduate student of an affiliated institution which is paying any applicable article publishing charge ("APC") for Open Access articles. Where the Submitting Author wishes to make the Work available on an Open Access basis (and intends to pay the relevant APC), the terms of reuse of such Open Access shall be governed by a Creative Commons licence – details of these licences and which Creative Commons licence will apply to this Work are set out in our licence referred to above.*

*Other than as permitted in any relevant BMJ Author's Self Archiving Policies, I confirm this Work has not been accepted for publication elsewhere, is not being considered for publication elsewhere and does not duplicate material already published. I confirm all authors consent to publication of this Work and authorise the granting of this licence.*

For peer review only

# Development and Validation of a multivariable prediction model for Multimorbidity Risk in middle-aged and older adults: A Nomogram approach

Xiao Zheng[a,c], Benli Xue[b,c], Shujuan Xiao[b,c], Xinru Li[b,c], Yimin Chen[b,c], Lei Shi[b,c], Xiaoyan Liang[a,c], Feng Tian[a,c] *, Chichen Zhang[b,a,c]*

[a] Department of Health Management, Shunde Hospital, Southern Medical University (The First People's Hospital of Shunde, Foshan), Foshan, China

[b] School of Health Management, Southern Medical University, Guangzhou, China

[c] Key Laboratory of Philosophy and Social Sciences of Guangdong Higher Education Institutions for Collaborative Innovation of Health Management Policy and Precision Health Service

*Correspondence to:

Chichen Zhang, zhangchichen@sina.com, School of Health Management, Southern Medical University, Guangzhou, China.

Feng Tian, Department of Health Management, Shunde Hospital, Southern Medical University (The First People's Hospital of Shunde, Foshan), Foshan, China

For peer review only



For peer review only - http://bmjopen.bmj.com/site/about/guidelines.xhtml

**Abstract**

**Background:** The aim of this study is to establish a self-simple-to-use nomogram to predict the risk of multimorbidity among middle-aged and older adults. **Methods:** We used data from the Chinese Longitudinal Healthy Longevity Survey, including 7735 samples. Samples' demographic characteristics, modifiable lifestyles, and depression were collected. Cox proportional hazard models and nomogram model were used to estimate the risk factors of multimorbidity.

**Results:** A total of 3576 (46.2%) participants have multimorbidity. The result showed that age, female (HR 1.23, 95%CI 1.10-1.36), chronic disease (HR 2.59, 95%CI 2.38-2.82), sleep time (HR 0.78, 95%CI 0.72-0.85), regular PA (HR 0.88, 95%CI 0.81-0.95), drinking (HR 1.27 95%CI 1.16-1.39), smoking (HR 1.40, 95%CI 1.26-1.53), BMI (HR 1.04, 95%CI 1.03-1.05) and depression scores (HR 1.02, 95%CI 1.01-1.03) were associated with multimorbidity. The C-index of nomogram models for derivation and validation sets were 0.70 (95%CI 0.69-0.71, *P*=0.006) and 0.71 (95%CI 0.70-0.73, *P*=0.008), respectively.

**Conclusions:** We have crafted a user-friendly nomogram model for predicting multimorbidity risk among middle-aged and older adults. This model integrates readily available and routinely assessed risk factors, enabling the early identification of high-risk individuals and offering tailored preventive and intervention strategies.

**Keywords**

Multimorbidity, Health risk appraisal, behaviors, Nomogram model, Health management, Longitudinal study



For peer review only - http://bmjopen.bmj.com/site/about/guidelines.xhtml

**STRENGTHS AND LIMITATIONS OF THIS STUDY**

1. We developed a user-friendly nomogram model to predict the risk of multimorbidity in middle-aged and older adults.

2. The model was constructed based on behavioral and household-level risk factors.

3. We have used a culling method to deal with the missing values, this may lead to sample bias and the extrapolation of the model needs to be careful.

For peer review only

## Background

Multimorbidity, commonly defined as the co-occurrence of two or more chronic conditions(1, 2), has emerged as a significant public health concern and poses challenges for healthcare systems. Extensive evidence(3) has shown that multimorbidity is associated with an increased risk of mortality(4), reduced quality of life, heightened healthcare utilization, and elevated health costs(5, 6). Thus, the prevention of multimorbidity has become a crucial focus for public health interventions. Consequently, it is essential to understand the prevalence trends of multimorbidity and the contributing factors within populations. This understanding will enable individuals to estimate and modify their personal risk of developing multimorbidity.

Although age is undeniably a well-established risk factor for multimorbidity(7), the multifaceted nature of this phenomenon demands a more comprehensive understanding that goes beyond age-related associations. Existing research has indeed



For peer review only - http://bmjopen.bmj.com/site/about/guidelines.xhtml

confirmed the higher prevalence of multimorbidity among older adults, with systematic reviews reporting rates ranging from 55% to 98% in the elderly population(8). Additionally, demographic factors such as female gender, and lower socioeconomic status have consistently been associated with an increased risk of multimorbidity. However, while these factors provide valuable insights, they represent only a fraction of the complex web of variables contributing to multimorbidity.

Previous studies have explored associations between sociodemographic factors, physical characteristics, and social networks with multimorbidity(9, 10). For example, numerous studies have linked poor sleep quality to an elevated risk of multimorbidity(11-14). Furthermore, the impact of body mass index (BMI) and smoking on multimorbidity prevalence underscores the intricate connections among these factors(15). Additionally, the well-documented relationship between depression and common chronic diseases has been established(16, 17), with longitudinal cohort studies demonstrating bidirectional associations between depression and multimorbidity(18).

Despite the substantial body of evidence regarding the associations among sociodemographic factors, social networks, lifestyle factors, depression, and the risk of developing multimorbidity, there remains a notable gap in the literature. This gap pertains to the absence of a comprehensive multivariable prediction model that integrates all these factors, providing a holistic assessment of multimorbidity risk. Our study seeks to address this gap by developing and validating a novel risk assessment



For peer review only - http://bmjopen.bmj.com/site/about/guidelines.xhtml

model that encompasses a broad spectrum of variables, including those mentioned above. Our aim is to equip individuals with a more accurate and personalized estimate of their risk of developing multimorbidity, contributing to a deeper understanding of this multifaceted health issue.

Wider determinants of health (WDHs) encompass a multitude of social, economic, political, and environmental factors that exert influence on health outcomes across an individual's lifespan. This influential model of health determinants places constitutional factors such as sex, age, and genetics at its core, surrounded by concentric layers that encompass individual lifestyle factors, followed by the broader determinants(19). While the core attributes remain relatively fixed, the determinants become more modifiable as the layers extend outward. Existing research has identified that individual lifestyle factors significantly contribute to multimorbidity among older adults. Chudasama also found that adopting a healthier lifestyle was associated with longer life expectancy for middle-aged adults, regardless of the presence of multimorbidity.(4).

In the context of the WDHs framework, individual behaviors constitute the innermost layer, presenting opportunities for modification, particularly through self-health management(20-22). The accurate assessment of one's risk of multimorbidity and the identification of potential risk factors represent critical initial steps in the journey of self-management. Therefore, the development of a user-friendly tool to assist individuals in estimating their risk of multimorbidity is of paramount significance.

A nomogram is a health risk appraisal model that offers individualized, evidence-



For peer review only
For peer review only - http://bmjopen.bmj.com/site/about/guidelines.xhtml

based, and highly accurate risk estimation (23, 24). It is easy to use and can facilitate self-management-related decision making. It is a user-friendly tool that facilitates decision-making related to self-management. In this study, we have developed the first nomogram for predicting the risk of multimorbidity among middle-aged and older adults.

## Methods

### Study population

We utilized data from the China Health and Retirement Longitudinal Study (CHARLS), a nationally representative survey of Chinese residents aged 45 years and above. The baseline survey was conducted in 2011 using a multistage probability sampling strategy and probability-proportional-to-size (PPS) sampling technique to ensure national representativeness. Follow-up waves were conducted in 2013, 2015, and 2018. Detailed information regarding the purpose, design, sample, and questionnaires used in CHARLS can be found in other studies(6, 25, 26). For this study, participants below the age of 45 and those with missing values in any variables were excluded from the analysis. The selection process is outlined in Additional file-Figure S1.

### Measurements

### Multimorbidity

In this study, multimorbidity was defined as the presence of two or more chronic non-communicable diseases, whether physical or psychological(6, 27). We assessed



For peer review only - http://bmjopen.bmj.com/site/about/guidelines.xhtml

multimorbidity by examining the presence of 14 specific non-communicable diseases. Physical chronic non-communicable diseases encompassed diagnosed conditions such as hypertension, dyslipidemia, diabetes, cancer, chronic lung disease, liver disease, heart disease, stroke, kidney disease, digestive disease, asthma, and arthritis. Psychological chronic non-communicable diseases included diagnosed emotional, nervous, or psychiatric problems, as well as memory-related diseases (all diseases were self-reported and diagnosed chronic conditions). To identify individuals with multimorbidity, we calculated the number of chronic diseases present for each participant. The outcome was the time to multimorbidity.

**The modifiable lifestyles**

This study included four well-known healthy lifestyle factors(4): physical activity (PA), smoking, alcohol consumption and diet behavior. Besides, sleep and social activity were included in this study.

The physical activity questionnaire used in CHARLS closely resembled the short version of the International Physical Activity Questionnaire (IPAQ)(28). However, some differences existed between CHARLS and IPAQ, such as assessing PA for a "usual week" instead of "the last 7 days" and lacking information on sedentariness. Additionally, instead of continuous values, four discrete time durations ("< 30 minutes" "≥30 minutes" "< 4 hours" and "≥ 4 hours") were collected(29). We calculated the median score for each intensity level and summed the number of different intensity levels using the metabolic equivalent (MET) as a reference. The weight of each intensity level was derived from the IPAQ scoring protocol. Detailed



For peer review only - http://bmjopen.bmj.com/site/about/guidelines.xhtml

information on PA and its calculation process can be found in the study by Li(26). The total weekly PA (MET-minutes/week) was calculated by multiplying the frequency, duration, and MET values. According to IPAQ, a minimum total PA of at least 600 MET-minutes/week was defined as regular PA, while <600 MET-minutes/week indicated a lack of regular PA.

Smoking was categorized as No (not current smoker) and Yes (current smoker) at the time of assessment. Alcohol consumption status was divided into 2 groups: No (Did not drink in the past 12 months or drinking frequency is less than weekly) and Yes (others). Regular eating behavior was determined based on the frequency of meals per day, with having three meals on time considered as regular eating.

Based on studies conducted in developed countries, respondents' total sleep duration was classified into five categories: <6 hours, 6 to <7 hours, 7 to <8 hours, 8 to <9 hours, and ≥9 hours(30, 31). According to the Healthy China initiative (2019-2030), the length of night-time sleep ≥ 7h was defined as enough sleep in this study (32). Social activity was categorized as "No" and "Yes" based on engagement in social activities within the past 12 months.

**Demographic characteristics**

Demographic characteristics included age, sex (male and female), marital status (others and married), residency (others and rural), education (primary education and below, secondary education and above), and BMI scores.

The covariates, including demographic characteristics and modifiable lifestyles factors were gathered by baseline questionnaire.



For peer review only - http://bmjopen.bmj.com/site/about/guidelines.xhtml

**Depression**

Depression was assessed using the Chinese version of the Center for Epidemiological Studies Depression scale (CES-D-10)(33). The CES-D-10 contains of 10 items with 4 response options: rare, some days (1–2 days), occasionally (3–4 days) and most of the time (5–7 days)(25). The scales for each of the 10 items were adjusted to 0, 1, 2 and 3, resulting in a CES-D-10 score ranging from 0 to 30, with higher scores indicating more negative feelings during the past week(34, 35).

**Statistical analysis**

The participants were randomly divided into a derivation set and a validation set at a ratio of 7:3. Participant characteristics, such as age and BMI, were summarized as mean ± SD and counts with proportions for categorical features. Cox proportional hazard models were used to estimate the associations between modifiable lifestyles (including physical activity, smoking, alcohol consumption, and diet behavior), depression, and other identified risk factors with the development of multimorbidity in middle-aged and older adults. Hazard ratios (HR) and 95% confidence intervals (95% CI) were reported for the total population. Factors with a significant level of less than 0.05 in the univariable regression model were entered into the multivariable Cox proportional hazard model for adjustment.

A nomogram was developed based on the results of the multivariable cox proportional hazard model in the derivation set. The nomogram assigns risk points to each variable by proportionally converting regression coefficients to a 0–100-point scale. The variable with the highest absolute value of the β coefficient is assigned 100 points.



For peer review only - http://bmjopen.bmj.com/site/about/guidelines.xhtml

The risk points for other variables are calculated based on the ratio of risk points to the $\beta$ coefficient of the highest variable. A prognostic index (PI) was calculated by summing the risk points corresponding to each weighted covariate. The nomogram was validated using the concordance index (C-index) calculated through 1000-fold bootstrap resampling to reduce overfit bias. The developed nomogram was then applied to the validation set. Model performance was further evaluated using a calibration curve, which superimposes both datasets for visual comparison of discrimination. All analyses were performed using R, version 3.0, $P<0.05$ was considered to indicate statistical significance.

**Patient and public involvement:** The public were not involved in the design, or conduct, or reporting, or dissemination plans of this research.

## Results

### Baseline characteristics

A total of 7,735 participants were included in this study, with 5,449 individuals in the derivation set and 2,286 in the validation set. The baseline characteristics of the study sample are presented in Table 1. The average age of participants in both datasets was 59.0±9.2 years, and in 2011, 3,726 individuals (48.2%) had at least one chronic disease.

**Table 1 Characteristics of the sample on the baseline-2011**

| Characteristics | *N (%) /S*$\pm\bar{X}$ | | |
|---|---|---|---|
| | **All** | **Derivation Set** | **Validation Set** |



For peer review only - http://bmjopen.bmj.com/site/about/guidelines.xhtml

| | | | |
|---|---|---|---|
| **Age (y)** | | | |
| <60 | 4387(56.7) | 3039(55.8) | 1348(59.0) |
| ≥60 | 3348(43.3) | 2410(44.2) | 938(41.0) |
| **Sex** | | | |
| Male | 3744(48.4) | 2684(49.3) | 1060(46.4) |
| Female | 3991(51.6) | 2765(50.7) | 1226(53.6) |
| **Hukou** | | | |
| Others | 6423(83.0) | 4504(82.7) | 1919(83.9) |
| Rural | 1312(17.0) | 945(17.3) | 367(16.1) |
| **Education** | | | |
| Primary education and below | 5296(68.5) | 3720(68.3) | 1576(68.9) |
| Secondary education and above | 2439(31.5) | 1729(31.7) | 710(31.1) |
| **Martial** | | | |
| Others | 1304(16.9) | 910(16.7) | 394(17.2) |
| Married | 6431(83.1) | 4539(83.3) | 1892(82.8) |
| **Chronic Disease** | | | |
| No | 4009(51.8) | 2817(51.7) | 1192(52.1) |
| Yes | 3726(48.2) | 2632(48.3) | 1094(47.9) |
| **Sleep (h)** | | | |
| <7 | 3803(49.2) | 2701(49.6) | 1102(48.2) |
| ≥7 | 3932(50.8) | 2748(50.4) | 1184(51.8) |
| **Social Activity** | | | |
| No | 3864(50.0) | 2747(50.4) | 1117(48.9) |
| Yes | 3871(50.0) | 2702(49.6) | 1169(51.1) |
| **Regular PA** | | | |
| No | 3422(44.2) | 2407(44.2) | 1015(44.4) |
| Yes | 4313(55.8) | 3042(55.8) | 1271(55.6) |
| **Regular Eating** | | | |



For peer review only - http://bmjopen.bmj.com/site/about/guidelines.xhtml

| | | | |
|---|---|---|---|
| No | 1009(13.0) | 707(13.0) | 302(13.2) |
| Yes | 6726(87.0) | 4742(87.0) | 1984(86.8) |
| **Smoke** | | | |
| No | 3379(43.7) | 2390(43.9) | 989(43.3) |
| Yes | 4356(56.3) | 3059(56.1) | 1297(56.7) |
| **Drink** | | | |
| No | 3354(43.4) | 2387(43.8) | 967(42.3) |
| Yes | 4381(56.6) | 3062(56.2) | 1319(57.7) |
| BMI | 23.32±3.69 | 23.31±3.72 | 23.35±3.62 |
| **Depression** | 9.13±4.57 | 9.10±4.56 | 9.21±4.60 |

**Prevalence of multimorbidity**

In 2018, a total of 3,576 participants (46.2%) were found to have multimorbidity. Among these individuals, the prevalence of multimorbidity was higher among older adults compared to those under the age of 60 (51.0% vs. 42.6%). Additionally, females exhibited a higher prevalence of multimorbidity compared to males (48.5% vs. 43.8%). Moreover, married individuals (45.7% vs. 48.6%) and those with higher education (42.1% vs. 48.1%) had a lower prevalence of multimorbidity compared to others (Figure 1).

**Figure 1. Prevalence of multimorbidity in the population**

**Predictor factors of multimorbidity**

The results of the univariable Cox proportional hazard model for the entire dataset (Additional file-Table S1) indicated that several factors were significantly associated with an increased risk of multimorbidity. These factors included older age, being female (HR 1.16, 95% CI 1.08-1.24), having existing chronic disease (HR 2.85,



For peer review only - http://bmjopen.bmj.com/site/about/guidelines.xhtml

For peer review only

95%CI 2.66-3.06), and higher depression scores (HR 1.04, 95%CI 1.03-1.05). Conversely, a higher level of education (HR 0.83, 95%CI 0.77-0.90), marital status (HR 0.91, 95%CI 0.83-0.99), higher BMI scores (HR 1.04, 95%CI 1.03-1.05), smoking (HR 1.46, 95%CI 1.36-1.56), drinking (1.46, 95%CI 1.36-1.56), getting at least 7 hours of sleep (HR 0.70, 95%CI 0.66-0.75), engaging in regular PA (HR 0.83, 95%CI 0.77-0.88) were associated with a decreased risk of multimorbidity.

The multivariable Cox proportional hazard models incorporated eleven significant variables identified through univariate analysis. Variable selection was performed using the backward selection method. In comparison to individuals under the age of 50, those in the age groups of 50-59 (HR 1.18, 95%CI 1.04-1.33), 60-69 (HR 1.44, 95%CI 1.27-1.64), and 70-79 (HR 1.23, 95%CI 1.04-1.45) exhibit a higher risk of multimorbidity. Furthermore, being female (HR 1.23, 95%CI 1.10-1.36), having a history of chronic disease (HR 2.59, 95%CI 2.38-2.82), getting at least 7 hours of sleep (HR 0.78, 95%CI 0.72-0.85), engaging in regular PA (HR 0.88, 95%CI 0.81-0.95), drinking (HR 1.27 95%CI 1.16-1.39), smoking (HR 1.40, 95%CI 1.26-1.53), higher BMI (HR 1.04, 95%CI 1.03-1.05) and increased depression scores (HR 1.02, 95%CI 1.01-1.03) were all found to be associated with multimorbidity in derivation set (Table 2).

**Table 2 Factors associated with the risk of multimorbidity (multivariable cox proportional hazard model)**

| Factors | Derivation Set | | | Validation Set | | |
|---|---|---|---|---|---|---|
| | *HR* | *95%CI* | *P* | *HR* | *95%CI* | *P* |



For peer review only - http://bmjopen.bmj.com/site/about/guidelines.xhtml

| | | | | | | |
|---|---|---|---|---|---|---|
| Age(<50) | | | | | | |
| Age(50-59) | 1.18 | 1.04-1.33 | 0.008 | 1.20 | 1.01-1.43 | 0.049 |
| Age(60-69) | 1.44 | 1.27-1.64 | <0.001 | 1.26 | 1.04-1.52 | 0.018 |
| Age(70-79) | 1.23 | 1.04-1.45 | 0.014 | 1.24 | 0.99-1.56 | 0.060 |
| Age(>=80) | 0.92 | 0.70-1.23 | 0.579 | 1.01 | 0.66-1.55 | 0.964 |
| Sex (Male) | | | | | | |
| Female | 1.23 | 1.10-1.36 | <0.001 | 1.43 | 1.22-1.67 | <0.001 |
| Chronic disease (No) | | | | | | |
| Yes | 2.59 | 2.38-2.82 | <0.001 | 2.70 | 2.37-3.06 | <0.001 |
| Sleep (<7h) | | | | | | |
| >=7h | 0.78 | 0.72-0.85 | <0.001 | 0.74 | 0.65-0.83 | <0.001 |
| Regular PA (No) | | | | | | |
| Yes | 0.88 | 0.81-0.95 | 0.001 | 0.88 | 0.77-0.99 | 0.039 |
| Drink (No) | | | | | | |
| Yes | 1.27 | 1.16-1.39 | <0.001 | 1.35 | 1.17-1.56 | <0.001 |
| Smoke (No) | | | | | | |
| Yes | 1.40 | 1.26-1.53 | <0.001 | 1.57 | 1.34-1.84 | <0.001 |
| BMI | 1.04 | 1.03-1.05 | <0.001 | 1.02 | 1.01-1.03 | 0.001 |
| Depression | 1.02 | 1.01-1.03 | <0.001 | 1.02 | 1.01-1.04 | 0.001 |

For peer review only

**Development and validation of an multimorbidity -predicting nomogram**

The PI was calculated based on the hazard ratio associated with the identified risk factors for multimorbidity. The nomogram was constructed using these results, with the BMI variable assigned a total scale of 100 and a range of 5 to 50. The risk score for BMI was determined to be 2.2. The risk scores for the other risk factors of multimorbidity were calculated proportionally to the β coefficient of BMI. So the PI=(0.3*I[age-45])+(14.3*I[female]) +(56.9*I[chronic disease])+(14.8*I[1-



For peer review only - http://bmjopen.bmj.com/site/about/guidelines.xhtml

sleep>=7h)+(6.4*I[1-regular PA])+(13.7*I[drink])+(19.1*I[smoke])+(2.2*BMI)+(1.4*depression), where I[ ]denotes the indicator function equal to 1 if the condition in parenthesis is met and 0 otherwise, except age. Based upon these findings, a nomogram was configured (Figure 2).

**Figure 2. The nomogram for multimorbidity risk prediction. Note: Draw a line perpendicular from the corresponding axis of each risk factor until it reaches the line labeled "POINTS". Sum up the number of points for all risk factors then draw a line descending from the labeled "TOTAL POINTS" until it intercepts each of the survival axes to determine 2-, 4-, and 7-year survival probabilities, multimorbidity probability=1- survival probability.**

The resulting nomogram was internally validated using the bootstrap validation method, and it demonstrated good accuracy in estimating the risk of multimorbidity, with a bootstrap-corrected C-index of 0.70 (95%CI 0.69-0.71, *P*=0.006) in the derivation set. Calibration plots also indicated good agreement between the risk estimation by the nomogram and the diagnosis of doctors, as depicted in Figure 2. When the estimates from the derivation set were applied to the validation set, a similar bootstrap-corrected C-index of 0.71 (95%CI 0.70-0.73, *P*=0.008) was obtained, along with a well-calibrated risk estimation curve (Figure 3).

**Figure 3. The predictive performance of the nomogram in estimating the risk of multimorbidity**



For peer review only - http://bmjopen.bmj.com/site/about/guidelines.xhtml

## Discussion

In this study, we observed a prevalence of multimorbidity of 46.2% among middle-aged and older adults. The prevalence of multimorbidity was higher among individuals with one chronic disease (62.8%) compared to those without chronic diseases (30.8%) at baseline. This result is consistent with the probability range of multimorbidity reported in a systematic study of the elderly(8). Although there were some differences in the estimation of multimorbidity compared to other studies, such as the number of included chronic diseases(36, 37), and the methods used to collect information(38). Our results confirmed the significant impact of multimorbidity on middle-aged and older adults, particularly among those with one chronic disease.

Our findings provide sufficient evidence for the association between older age and multimorbidity, which is consistent with similar trends observed in countries such as Singapore(39), Ireland, and Scotland(27). The prevalence of multimorbidity among females is higher than males. Additionally, we found a higher prevalence of multimorbidity among females compared to males, which is supported by studies from various countries indicating that older men have a lower risk of multimorbidity than their female counterparts(27, 38). However, a study by Lian found that the onset of multimorbidity occurs at an earlier age in males than in females(39). This is also a new idea to understand the sex-differ in multimorbidity, but our study only included middle-aged and older adults aged over 45 years, we cannot evaluate this result of Lian. Future research could explore the prevalence and different combinations of chronic conditions in people with multimorbidity across various age and sex groups.



For peer review only - http://bmjopen.bmj.com/site/about/guidelines.xhtml

Existing studies have established a link between short sleep duration and multimorbidity. Experimental evidence confirms the deleterious effects of sleep deprivation on endocrine, immune, neurovitality and inflammatory pathways(40). For instance, Maria Ruiz-Castell found an association between short sleep duration and the number of chronic conditions(41). Helbig also observed a significant positive relationship between short sleep duration and multimorbidity among women(42). High-risk lifestyles, such as smoking, excessive alcohol consumption, poor diet, physical inactivity, and unhealthy body shape, have also been confirmed as contributing factors to multimorbidity(43). Smoking and excessive alcohol consumption remain leading risk factors for early death and disability globally(44, 45). In our study, we found that smoking and drinking increased the risk of multimorbidity, with the highest risk index among all unhealthy behaviors. The expansion of tobacco and alcohol control measures remains a significant public health priority worldwide. Mika conducted an observational study using data from two Finnish cohort studies comprising 614,014 adults, and the results showed that obesity is a significant factor in multimorbidity(46). Similarly, our study found that higher BMI was associated with an increased risk of multimorbidity among middle-aged and older adults. There exists a bidirectional association between depression and multimorbidity(16, 47). Depression increases the risk of multimorbidity, while having multimorbidity also raises the risk of depression. Our study found the risk of multimorbidity for middle-aged and older adults with higher depression scores.

Based on our results, we developed a user-friendly nomogram model for predicting



For peer review only - http://bmjopen.bmj.com/site/about/guidelines.xhtml

the risk of multimorbidity. One of the most appealing aspects of our nomogram model is its home applicability and ease of use by individuals. For example, a 50-year-old married male with primary education, one chronic disease, a history of smoking and excessive alcohol consumption, irregular physical activity, 8 hours of sleep, a depression score of 16, and a BMI of 24.9 would have a total risk score of 219.3 points. This corresponds to a 2-, 4-, and 7-year probability of multimorbidity of 32%, 71%, and <10%, respectively. Based on the calculated results, individuals can develop self-management strategies to reduce their risk of multimorbidity.

**Limitation**

Our study had several limitations. First, the model was constructed based on behavioral and household-level risk factors, limiting its applicability to clinical prediction. Additionally, in this study, there is a large proportion of missing data for some important variables, so we have used a culling method to deal with the missing values. As a result, this may lead to sample bias and the extrapolation of the model needs to be careful. We also need to validate the model using external data.

## Conclusions

This study confirms the severity of multimorbidity among middle-aged and older adults, particularly among those who already have one chronic disease. Age showed a significant correlation with multimorbidity, and the prevalence of multimorbidity was higher in females compared to males. In addition, insufficient sleep, smoking, drinking, obesity, and depressive symptoms were also associated with multimorbidity.



For peer review only - http://bmjopen.bmj.com/site/about/guidelines.xhtml

For peer review only

Based on these findings, we developed a user-friendly nomogram model to predict the risk of multimorbidity in middle-aged and older adults. Our research not only builds upon the existing body of knowledge but also introduces a novel and comprehensive approach to assessing multimorbidity risk, which is of significant clinical and public health relevance. The multivariable prediction model provides valuable tools for healthcare professionals to manage multimorbidity.

For peer review only

## Ethics statements

### Patient consent for publication

Not applicable.

### Ethics approval

The Biomedical Ethics Review Committee of Peking University approved CHARLS, and all participants were required to provide written informed consent. The ethical approval number was IRB00001052-11015.

## Supplementary materials

Additional file.

## Footnotes

**Contributors:** ZCC and TF conceived the study. ZX and ZCC were major contributors in writing the manuscript. XBL, XSJ, LXR, analyzed and interpreted the patient data. CYM SL and LXY edited and contributed content to the final draft. All



For peer review only - http://bmjopen.bmj.com/site/about/guidelines.xhtml

authors read and approved the final manuscript.

**Funding:** This study was funded by the Guangdong Basic and Applied Basic Research Foundation (No.2022A1515110295, No.2022A1515011591), National Nature Science Foundation of China (No.72274091), China Postdoctoral Science Foundation (No. 2022M721539), Guangdong Philosophy and Social Science Foundation (No. GD23CGL06).

**Competing interests:** The authors declare that they have no competing interests.

**Availability of data and materials:** The datasets analysed during the current study are available from the corresponding author on reasonable request.

## References

1. Fortin M, Bravo G, Hudon C, Vanasse A, Lapointe L. Prevalence of multimorbidity among adults seen in family practice. Ann Fam Med. 2005;3(3):223-8.

2. Jiao DD, Watanabe K, Sawada Y, Tanaka E, Watanabe T, Tomisaki E, et al. Multimorbidity and functional limitation: the role of social relationships. Arch Gerontol Geriat. 2021;92.

3. Nicholson K, Griffith LE, Sohel N, Raina P. Examining early and late onset of multimorbidity in the Canadian Longitudinal Study on Aging. J Am Geriatr Soc. 2021.

4. Chudasama YV, Khunti K, Gillies CL, Dhalwani NN, Davies MJ, Yates T, et al. Healthy lifestyle and life expectancy in people with multimorbidity in the UK



For peer review only - http://bmjopen.bmj.com/site/about/guidelines.xhtml

Biobank: A longitudinal cohort study. PLoS Med. 2020;17(9):e1003332.

5. Ding D, Lawson KD, Kolbe-Alexander TL, Finkelstein EA, Katzmarzyk PT, van Mechelen W, et al. The economic burden of physical inactivity: a global analysis of major non-communicable diseases. Lancet. 2016;388(10051):1311-24.

6. Zhao Y, Atun R, Oldenburg B, McPake B, Tang S, Mercer SW, et al. Physical multimorbidity, health service use, and catastrophic health expenditure by socioeconomic groups in China: an analysis of population-based panel data. The Lancet Global Health. 2020;8(6):e840-e9.

7. Zheng X, Cui Y, Xue Y, Shi L, Guo Y, Dong F, Zhang C. Adverse childhood experiences in depression and the mediating role of multimorbidity in mid-late life: A nationwide longitudinal study. J Affect Disord. 2022 Mar 15;301:217-224.

8. Marengoni A, Angleman S, Melis R, Mangialasche F, Karp A, Garmen A, et al. Aging with multimorbidity: A systematic review of the literature. Ageing Res Rev. 2011;10(4):430-9.

9. Xue B, Xue Y, Dong F, Zheng X, Shi L, Xiao S, Zhang J, Ou W, Wang Q, Zhang C. The impact of socioeconomic status and sleep quality on the prevalence of multimorbidity in older adults. Front Public Health. 2022 Sep 26;10:959700.

10. Olaya B, Domenech-Abella J, Moneta MV, Lara E, Caballero FF, Rico-Uribed LA, et al. All-cause mortality and multimorbidity in older adults: The role of social support and loneliness. Exp Gerontol. 2017;99:120-6.

11. Bertuol C, Tozetto WR, Streb AR, Del Duca GF. Combined relationship of physical inactivity and sedentary behaviour with the prevalence of noncommunicable



For peer review only - http://bmjopen.bmj.com/site/about/guidelines.xhtml

chronic diseases: data from 52,675 Brazilian adults and elderly. Eur J Sport Sci. 2021.

12. Lindstrom J, Uusitupa M. Lifestyle intervention, diabetes, and cardiovascular disease. Lancet. 2008;371(9626):1731-3.

13. Elwood P, Galante J, Pickering J, Palmer S, Bayer A, Ben-Shlomo Y, et al. Healthy Lifestyles Reduce the Incidence of Chronic Diseases and Dementia: Evidence from the Caerphilly Cohort Study. Plos One. 2013;8(12).

14. Hu FB, Liu Y, Willett WC. Preventing chronic diseases by promoting healthy diet and lifestyle: public policy implications for China. Obes Rev. 2011;12(7):552-9.

15. Fortin M, Haggerty J, Almirall J, Bouhali T, Sasseville M, Lemieux M. Lifestyle factors and multimorbidity: a cross sectional study. BMC Public Health. 2014;14:686.

16. Birk JL, Kronish IM, Moise N, Falzon L, Yoon S, Davidson KW. Depression and Multimorbidity: Considering Temporal Characteristics of the Associations Between Depression and Multiple Chronic Diseases. Health Psychology. 2019;38(9):802-11.

17. Meng L, Chen DM, Yang Y, Zheng Y, Hui RT. Depression increases the risk of hypertension incidence: a meta-analysis of prospective cohort studies. J Hypertens. 2012;30(5):842-51.

18. Qiao YN, Liu SY, Zhang YX, Wu Y, Shen YP, Ke CF. Bidirectional association between depression and multimorbidity in middle-aged and elderly Chinese adults: a longitudinal cohort study. Aging & Mental Health. 2021.

19. Bambra C, Gibson M, Sowden A, Wright K, Whitehead M, Petticrew M. Tackling the wider social determinants of health and health inequalities: evidence from systematic reviews. J Epidemiol Commun H. 2010;64(4):284-91.



For peer review only

20. Chodosh J, Morton SC, Mojica W, Maglione M, Suttorp MJ, Hilton L, et al. Meta-analysis: Chronic disease self-management programs for older adults. Ann Intern Med. 2005;143(6):427-38.

21. Warsi A, Wang PS, LaValley MP, Avorn J, Solomon DH. Self-management education programs in chronic disease - A systematic review and methodological critique of the literature. Arch Intern Med. 2004;164(15):1641-9.

22. Li WY, Chiu FC, Zeng JK, Li YW, Huang SH, Yeh HC, et al. Mobile Health App With Social Media to Support Self-Management for Patients With Chronic Kidney Disease: Prospective Randomized Controlled Study. Journal of Medical Internet Research. 2020;22(12).

23. Lei Z, Li J, Wu D, Xia Y, Wang Q, Si A, et al. Nomogram for Preoperative Estimation of Microvascular Invasion Risk in Hepatitis B Virus-Related Hepatocellular Carcinoma Within the Milan Criteria. JAMA Surg. 2016;151(4):356-63.

24. B OH, Gransar H, Callister T, Shaw LJ, Schulman-Marcus J, Stuijfzand WJ, et al. Development and Validation of a Simple-to-Use Nomogram for Predicting 5-, 10-, and 15-Year Survival in Asymptomatic Adults Undergoing Coronary Artery Calcium Scoring. JACC Cardiovasc Imaging. 2018;11(3):450-8.

25. Jing R, Xu T, Rong H, Lai X, Fang H. Longitudinal Association Between Sleep Duration and Depressive Symptoms in Chinese Elderly. Nat Sci Sleep. 2020;12:737-47.

26. Li X, Zhang W, Zhang W, Tao K, Ni W, Wang K, et al. Level of physical activity



For peer review only - http://bmjopen.bmj.com/site/about/guidelines.xhtml

among middle-aged and older Chinese people: evidence from the China health and retirement longitudinal study. BMC Public Health. 2020;20(1):1682.

27. Barnett K, Mercer SW, Norbury M, Watt G, Wyke S, Guthrie B. Epidemiology of multimorbidity and implications for health care, research, and medical education: a cross-sectional study. Lancet. 2012;380(9836):37-43.

28. Craig CL, Marshall AL, Sjostrom M, Bauman AE, Booth ML, Ainsworth BE, et al. International physical activity questionnaire: 12-country reliability and validity. Med Sci Sports Exerc. 2003;35(8):1381-95.

29. Zhao Y, Hu Y, Smith JP, Strauss J, Yang G. Cohort profile: the China Health and Retirement Longitudinal Study (CHARLS). Int J Epidemiol. 2014;43(1):61-8.

30. Yang L, Yang H, He M, Pan A, Li X, Min X, et al. Longer Sleep Duration and Midday Napping Are Associated with a Higher Risk of CHD Incidence in Middle-Aged and Older Chinese: the Dongfeng-Tongji Cohort Study. Sleep. 2016;39(3):645-52.

31. Paudel M, Taylor BC, Ancoli-Israel S, Blackwell T, Maglione JE, Stone K, et al. Sleep Disturbances and Risk of Depression in Older Men. Sleep. 2013;36(7):1033-40.

32. China TPsRo. Healthy China initiative (2019-2030) 2019 [Available from: http://www.gov.cn/xinwen/2019-07/15/content_5409694.htm.

33. Jin Y, Luo Y, He P. Hypertension, socioeconomic status and depressive symptoms in Chinese middle-aged and older adults: Findings from the China health and retirement longitudinal study. J Affect Disord. 2019;252:237-44.

34. Wang RX, Chen Z, Zhou YJ, Shen LN, Zhang Z, Wu X. Melancholy or



For peer review only - http://bmjopen.bmj.com/site/about/guidelines.xhtml

mahjong? Diversity, frequency, type, and rural-urban divide of social participation and depression in middle- and old-aged Chinese: A fixed-effects analysis. Soc Sci Med. 2019;238.

35. Chen Z, Ying J, Ingles J, Zhang D, Rajbhandari-Thapa J, Wang R, et al. Gender differential impact of bereavement on health outcomes: evidence from the China Health and Retirement Longitudinal Study, 2011-2015. BMC Psychiatry. 2020;20(1):514.

36. Diederichs C, Berger K, Bartels DB. The Measurement of Multiple Chronic Diseases-A Systematic Review on Existing Multimorbidity Indices. J Gerontol a-Biol. 2011;66(3):301-11.

37. Guisado-Clavero M, Roso-Llorach A, Lopez-Jimenez T, Pons-Vigues M, Foguet-Boreu Q, Munoz MA, et al. Multimorbidity patterns in the elderly: a prospective cohort study with cluster analysis. Bmc Geriatrics. 2018;18.

38. Hewitt J, McCormack C, Tay HS, Greig M, Law J, Tay A, et al. Prevalence of multimorbidity and its association with outcomes in older emergency general surgical patients: an observational study. Bmj Open. 2016;6(3).

39. Low LL, Kwan YH, Ko MSM, Yeam CT, Lee VSY, Tan WB, et al. Epidemiologic Characteristics of Multimorbidity and Sociodemographic Factors Associated With Multimorbidity in a Rapidly Aging Asian Country. JAMA Netw Open. 2019;2(11):e1915245.

40. Ferrie JE, Kivimaki M, Akbaraly TN, Singh-Manoux A, Miller MA, Gimeno D, et al. Associations Between Change in Sleep Duration and Inflammation: Findings on



For peer review only - http://bmjopen.bmj.com/site/about/guidelines.xhtml

C-reactive Protein and Interleukin 6 in the Whitehall II Study. American Journal of Epidemiology. 2013;178(6):956-61.

41. Ruiz-Castell M, Makovski TT, Bocquet V, Stranges S. Sleep duration and multimorbidity in Luxembourg: results from the European Health Examination Survey in Luxembourg, 2013-2015. BMJ Open. 2019;9(8):e026942.

42. Helbig AK, Stockl D, Heier M, Thorand B, Schulz H, Peters A, et al. Relationship between sleep disturbances and multimorbidity among community-dwelling men and women aged 65-93 years: results from the KORA Age Study. Sleep Med. 2017;33:151-9.

43. Han Y, Hu Y, Yu C, Guo Y, Pei P, Yang L, et al. Lifestyle, cardiometabolic disease, and multimorbidity in a prospective Chinese study. Eur Heart J. 2021;42(34):3374-84.

44. Beaglehole R, Bates C, Youdan B, Bonita R. Nicotine without smoke: fighting the tobacco epidemic with harm reduction. Lancet. 2019;394(10200):718-20.

45. Mohammad KA, Salomon JA. Smoking prevalence and attributable disease burden in 195 countries and territories, 1990-2015: a systematic analysis from the Global Burden of Disease Study 2015 (vol 389, pg 1885, 2015). Lancet. 2017;390(10103):1644-.

46. Kivimaki M, Strandberg T, Pentti J, Nyberg ST, Frank P, Jokela M, et al. Body-mass index and risk of obesity-related complex multimorbidity: an observational multicohort study. Lancet Diabetes Endocrinol. 2022;10(4):253-63.

47. Huang CQ, Dong BR, Lu ZC, Yue JR, Liu QX. Chronic diseases and risk for



For peer review only - http://bmjopen.bmj.com/site/about/guidelines.xhtml

depression in old age: A meta-analysis of published literature. Ageing Res Rev. 2010;9(2):131-41.

For peer review only



For peer review only - http://bmjopen.bmj.com/site/about/guidelines.xhtml

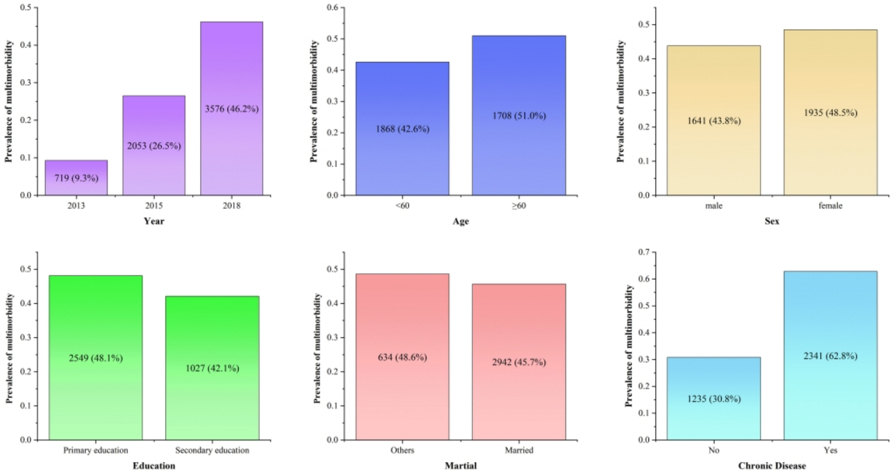


Figure 1. Prevalence of multimorbidity in the population.

338x179mm (300 x 300 DPI)

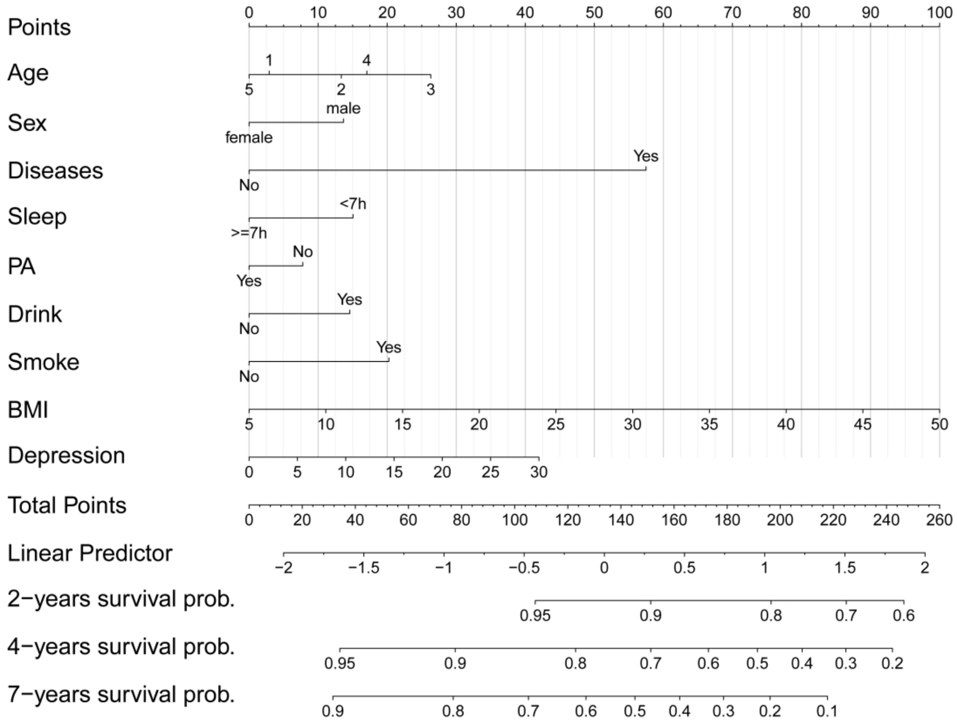


Figure 2. The nomogram for multimorbidity risk prediction. Note: Draw a line perpendicular from the corresponding axis of each risk factor until it reaches the line labeled "POINTS". Sum up the number of points for all risk factors then draw a line descending from the labeled "TOTAL POINTS" until it intercepts each of the survival axes to determine 2-, 4-, and 7-year survival probabilities, multimorbidity probability=1- survival probability.

728x529mm (118 x 118 DPI)

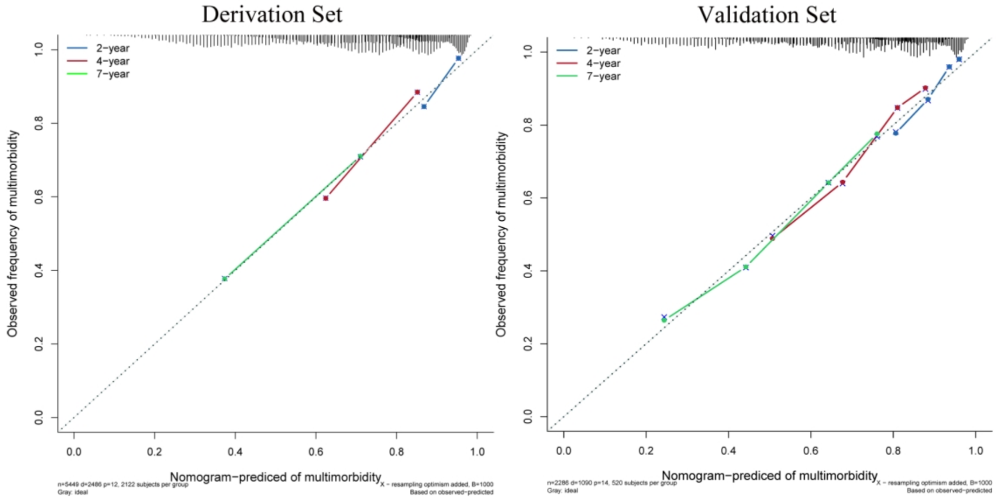


Figure 3. The predictive performance of the nomogram in estimating the risk of multimorbidity

406x210mm (118 x 118 DPI)

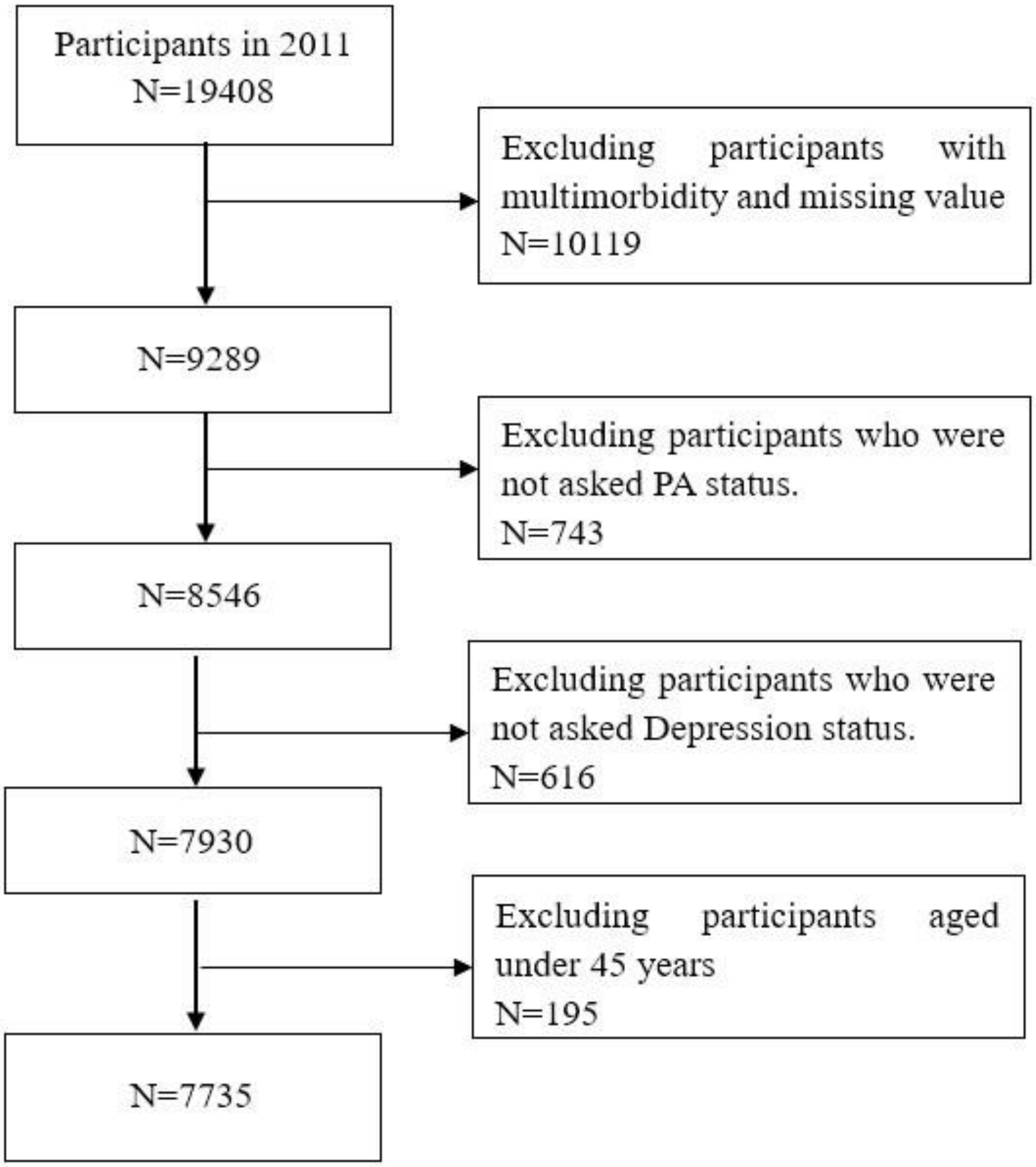


**Figure S1. Flow diagram of Sample selection**

**Table S1 Factors associated with the risk of multimorbidity (univariable cox proportional hazard models, N=7735)**

| **Characteristics** | | ***HR*** | ***95%CI*** | ***P*** |
|---|---|---|---|---|
| **Age (y)** | <50* | | | |
| | 50-69 | 1.25 | 1.13-1.39 | <0.001 |
| | 60-69 | 1.58 | 1.42-1.74 | <0.001 |
| | 70-79 | 1.41 | 1.25-1.60 | <0.001 |
| | >=80 | 1.12 | 0.90-1.41 | 0.311 |
| **Sex** | Male* | | | |
| | Female | 1.16 | 1.08-1.24 | <0.001 |
| **Hukou** | Rural* | | | |
| | Others | 1.03 | 0.95-1.13 | 0.452 |
| **Education** | Primary* | | | |
| | Secondary | 0.83 | 0.77-0.90 | <0.001 |
| **Marial** | Others* | | | |
| | Married | 0.91 | 0.83-0.99 | 0.027 |
| **Chronic Diseases** | No* | | | |
| | Yes | 2.85 | 2.66-3.06 | <0.001 |
| **Sleep (h)** | <7* | | | |
| | >=7 | 0.70 | 0.66-0.75 | <0.001 |
| **Social Activity** | No* | | | |
| | Yes | 0.96 | 0.9-1.02 | 0.215 |
| **Regular PA** | No* | | | |
| | Yes | 0.83 | 0.77-0.88 | <0.001 |
| **Eating** | No* | | | |
| | Yes | 0.97 | 0.88-1.07 | 0.594 |
| **Smoke** | No* | | | |
| | Yes | 1.46 | 1.36-1.56 | <0.001 |
| **Drink** | No* | | | |
| | Yes | 1.46 | 1.36-1.56 | <0.001 |
| **BMI** | | 1.04 | 1.03-1.05 | <0.001 |
| **Depression** | | 1.04 | 1.03-1.05 | <0.001 |

For peer review only

# TRIPOD Checklist: Prediction Model Development and Validation

| Section/Topic | Item | | Checklist Item | Page |
|---|---|---|---|---|
| **Title and abstract** | | | | |
| Title | 1 | D;V | Identify the study as developing and/or validating a multivariable prediction model, the target population, and the outcome to be predicted. | 1 |
| Abstract | 2 | D;V | Provide a summary of objectives, study design, setting, participants, sample size, predictors, outcome, statistical analysis, results, and conclusions. | 2 |
| **Introduction** | | | | |
| Background and objectives | 3a | D;V | Explain the medical context (including whether diagnostic or prognostic) and rationale for developing or validating the multivariable prediction model, including references to existing models. | 3-5 |
| | 3b | D;V | Specify the objectives, including whether the study describes the development or validation of the model or both. | 5-6 |
| **Methods** | | | | |
| Source of data | 4a | D;V | Describe the study design or source of data (e.g., randomized trial, cohort, or registry data), separately for the development and validation data sets, if applicable. | 6 |
| | 4b | D;V | Specify the key study dates, including start of accrual; end of accrual; and, if applicable, end of follow-up. | 6 |
| S1Participants | 5a | D;V | Specify key elements of the study setting (e.g., primary care, secondary care, general population) including number and location of centres. | 6 |
| | 5b | D;V | Describe eligibility criteria for participants. | 6, Figure S1 |
| | 5c | D;V | Give details of treatments received, if relevant. | |
| Outcome | 6a | D;V | Clearly define the outcome that is predicted by the prediction model, including how and when assessed. | 6-7 |
| | 6b | D;V | Report any actions to blind assessment of the outcome to be predicted. | |
| Predictors | 7a | D;V | Clearly define all predictors used in developing or validating the multivariable prediction model, including how and when they were measured. | 7-9 |
| | 7b | D;V | Report any actions to blind assessment of predictors for the outcome and other predictors. | |
| Sample size | 8 | D;V | Explain how the study size was arrived at. | 6, Figure S1 |
| Missing data | 9 | D;V | Describe how missing data were handled (e.g., complete-case analysis, single imputation, multiple imputation) with details of any imputation method. | 6, Figure S1 |
| Statistical analysis methods | 10a | D | Describe how predictors were handled in the analyses. | 7-9 |
| | 10b | D | Specify type of model, all model-building procedures (including any predictor selection), and method for internal validation. | 9-10 |
| | 10c | V | For validation, describe how the predictions were calculated. | 9-10 |
| | 10d | D;V | Specify all measures used to assess model performance and, if relevant, to compare multiple models. | 9-10 |
| | 10e | V | Describe any model updating (e.g., recalibration) arising from the validation, if done. | 9-10 |
| Risk groups | 11 | D;V | Provide details on how risk groups were created, if done. | |
| Development vs. validation | 12 | V | For validation, identify any differences from the development data in setting, eligibility criteria, outcome, and predictors. | 9-10 |
| **Results** | | | | |
| Participants | 13a | D;V | Describe the flow of participants through the study, including the number of participants with and without the outcome and, if applicable, a summary of the follow-up time. A diagram may be helpful. | Figure S1 |
| | 13b | D;V | Describe the characteristics of the participants (basic demographics, clinical features, available predictors), including the number of participants with missing data for predictors and outcome. | Table 1 |
| | 13c | V | For validation, show a comparison with the development data of the distribution of important variables (demographics, predictors and outcome). | Table 1 |
| Model development | 14a | D | Specify the number of participants and outcome events in each analysis. | 11-12 |
| | 14b | D | If done, report the unadjusted association between each candidate predictor and outcome. | 12-13, Table S1 |
| Model specification | 15a | D | Present the full prediction model to allow predictions for individuals (i.e., all regression coefficients, and model intercept or baseline survival at a given time point). | 13-14, Table 2 |
| | 15b | D | Explain how to the use the prediction model. | 14, Figure 2 |
| Model performance | 16 | D;V | Report performance measures (with CIs) for the prediction model. | 15 |
| Model-updating | 17 | V | If done, report the results from any model updating (i.e., model specification, model performance). | |
| **Discussion** | | | | |
| Limitations | 18 | D;V | Discuss any limitations of the study (such as nonrepresentative sample, few events per predictor, missing data). | 18 |
| Interpretation | 19a | V | For validation, discuss the results with reference to performance in the development data, and any other validation data. | 15-17 |
| | 19b | D;V | Give an overall interpretation of the results, considering objectives, limitations, results from similar studies, and other relevant evidence. | 15-17 |
| Implications | 20 | D;V | Discuss the potential clinical use of the model and implications for future research. | 17-19 |

For peer review only

TRIPOD Checklist: Prediction Model Development and Validation

| Other information | | | | |
|---|---|---|---|---|
| Supplementary information | 21 | D;V | Provide information about the availability of supplementary resources, such as study protocol, Web calculator, and data sets. | |
| Funding | 22 | D;V | Give the source of funding and the role of the funders for the present study. | 19 |

*Items relevant only to the development of a prediction model are denoted by D, items relating solely to a validation of a prediction model are denoted by V, and items relating to both are denoted D;V. We recommend using the TRIPOD Checklist in conjunction with the TRIPOD Explanation and Elaboration document.

For peer review only

BMJ Open

# Development and validation of a prediction nomogram for multimorbidity risk in middle-aged and older adults: a retrospective cohort study

| | |
|---|---|
| Journal: | *BMJ Open* |
| Manuscript ID | bmjopen-2023-077573.R2 |
| Article Type: | Original research |
| Date Submitted by the Author: | 16-Oct-2023 |
| Complete List of Authors: | Zheng, Xiao; Shunde Hospital, Southern Medical University (The First People's Hospital of Shunde, Foshan), Department of Health Management<br>Xue, Benli; Southern Medical University<br>Xiao, Shujuan; Southern Medical University<br>Li, Xinru; Southern Medical University<br>Chen, Yimin; Southern Medical University<br>Shi, Lei; Southern Medical University<br>Liang, Xiaoyan; Shunde Hospital, Southern Medical University (The First People's Hospital of Shunde, Foshan), Department of Health Management<br>Tian, Feng; Shunde Hospital, Southern Medical University (The First People's Hospital of Shunde, Foshan), Department of Health Management<br>Zhang, Chichen; Southern Medical University |
| <b>Primary Subject Heading</b>: | Health services research |
| Secondary Subject Heading: | General practice / Family practice |
| Keywords: | Aging, China, Chronic Disease |
| | |

SCHOLARONE™
Manuscripts

*I, the Submitting Author has the right to grant and does grant on behalf of all authors of the Work (as defined in the below author licence), an exclusive licence and/or a non-exclusive licence for contributions from authors who are: i) UK Crown employees; ii) where BMJ has agreed a CC-BY licence shall apply, and/or iii) in accordance with the terms applicable for US Federal Government officers or employees acting as part of their official duties; on a worldwide, perpetual, irrevocable, royalty-free basis to BMJ Publishing Group Ltd ("BMJ") its licensees and where the relevant Journal is co-owned by BMJ to the co-owners of the Journal, to publish the Work in this journal and any other BMJ products and to exploit all rights, as set out in our licence.*

*The Submitting Author accepts and understands that any supply made under these terms is made by BMJ to the Submitting Author unless you are acting as an employee on behalf of your employer or a postgraduate student of an affiliated institution which is paying any applicable article publishing charge ("APC") for Open Access articles. Where the Submitting Author wishes to make the Work available on an Open Access basis (and intends to pay the relevant APC), the terms of reuse of such Open Access shall be governed by a Creative Commons licence – details of these licences and which Creative Commons licence will apply to this Work are set out in our licence referred to above.*

*Other than as permitted in any relevant BMJ Author's Self Archiving Policies, I confirm this Work has not been accepted for publication elsewhere, is not being considered for publication elsewhere and does not duplicate material already published. I confirm all authors consent to publication of this Work and authorise the granting of this licence.*

For peer review only

# Development and validation of a prediction nomogram for multimorbidity risk in middle-aged and older adults: a retrospective cohort study

Xiao Zheng[a,c], Benli Xue[b,c], Shujuan Xiao[b,c], Xinru Li[b,c], Yimin Chen[b,c], Lei Shi[b,c], Xiaoyan Liang[a,c], Feng Tian[a,c] *, Chichen Zhang[b,a,c]*

[a] Department of Health Management, Shunde Hospital, Southern Medical University (The First People's Hospital of Shunde, Foshan), Foshan, China

[b] School of Health Management, Southern Medical University, Guangzhou, China

[c] Key Laboratory of Philosophy and Social Sciences of Guangdong Higher Education Institutions for Collaborative Innovation of Health Management Policy and Precision Health Service

*Correspondence to:

Chichen Zhang, zhangchichen@sina.com, School of Health Management, Southern Medical University, Guangzhou, China.

Feng Tian, Department of Health Management, Shunde Hospital, Southern Medical University (The First People's Hospital of Shunde, Foshan), Foshan, China

For peer review only



For peer review only - http://bmjopen.bmj.com/site/about/guidelines.xhtml

**Abstract**

**Objectives:** The aim of this study is to establish a self-simple-to-use nomogram to predict the risk of multimorbidity among middle-aged and older adults.

**Design:** A retrospective cohort study.

**Participants:** We used data from the Chinese Longitudinal Healthy Longevity Survey, including 7735 samples.

**Main outcome measures:** Samples' demographic characteristics, modifiable lifestyles, and depression were collected. Cox proportional hazard models and nomogram model were used to estimate the risk factors of multimorbidity.

**Results:** A total of 3576 (46.2%) participants have multimorbidity. The result showed that age, female (HR 1.23, 95%CI 1.10-1.36), chronic disease (HR 2.59, 95%CI 2.38-2.82), sleep time (HR 0.78, 95%CI 0.72-0.85), regular PA (HR 0.88, 95%CI 0.81-0.95), drinking (HR 1.27 95%CI1.16-1.39), smoking (HR 1.40, 95%CI 1.26-1.53), BMI (HR 1.04, 95%CI 1.03-1.05) and depression scores (HR 1.02, 95%CI 1.01-1.03) were associated with multimorbidity. The C-index of nomogram models for derivation and validation sets were 0.70 (95%CI 0.69-0.71, *P*=0.006) and 0.71 (95%CI 0.70-0.73, *P*=0.008), respectively.

**Conclusions:** We have crafted a user-friendly nomogram model for predicting multimorbidity risk among middle-aged and older adults. This model integrates readily available and routinely assessed risk factors, enabling the early identification of high-risk individuals and offering tailored preventive and intervention strategies.


**Keywords**



For peer review only

For peer review only - http://bmjopen.bmj.com/site/about/guidelines.xhtml

Multimorbidity, Health risk appraisal, behaviors, Nomogram model, Health management, Longitudinal study

**STRENGTHS AND LIMITATIONS OF THIS STUDY**

1. We developed a user-friendly nomogram model to predict the risk of multimorbidity in middle-aged and older adults.

2. The model was constructed based on behavioral and household-level risk factors.

3. This is the first study to develop and validate a prediction nomogram for multimorbidity risk in middle-aged and older adults.

4. We have used a culling method to deal with the missing values, this may lead to sample bias and the extrapolation of the model needs to be careful.

5. We could not compare the performance of the nomogram model with different models.

## Background

Multimorbidity, commonly defined as the co-occurrence of two or more chronic conditions(1, 2), has emerged as a significant public health concern and poses challenges for healthcare systems. Extensive evidence(3) has shown that multimorbidity is associated with an increased risk of mortality(4), reduced quality of life, heightened healthcare utilization, and elevated health costs(5, 6). Thus, the prevention of multimorbidity has become a crucial focus for public health interventions. Consequently, it is essential to understand the prevalence trends of multimorbidity and the contributing factors within populations. This understanding will enable individuals



For peer review only - http://bmjopen.bmj.com/site/about/guidelines.xhtml

to estimate and modify their personal risk of developing multimorbidity.

Although age is undeniably a well-established risk factor for multimorbidity(7), the multifaceted nature of this phenomenon demands a more comprehensive understanding that goes beyond age-related associations. Existing research has indeed confirmed the higher prevalence of multimorbidity among older adults, with systematic reviews reporting rates ranging from 55% to 98% in the elderly population(8). Additionally, demographic factors such as female gender, and lower socioeconomic status have consistently been associated with an increased risk of multimorbidity. However, while these factors provide valuable insights, they represent only a fraction of the complex web of variables contributing to multimorbidity.

Previous studies have explored associations between sociodemographic factors, physical characteristics, and social networks with multimorbidity(9, 10). For example, numerous studies have linked poor sleep quality to an elevated risk of multimorbidity(11-14). Furthermore, the impact of body mass index (BMI) and smoking on multimorbidity prevalence underscores the intricate connections among these factors(15). Additionally, the well-documented relationship between depression and common chronic diseases has been established(16, 17), with longitudinal cohort studies demonstrating bidirectional associations between depression and multimorbidity(18).

Despite the substantial body of evidence regarding the associations among sociodemographic factors, social networks, lifestyle factors, depression, and the risk of developing multimorbidity, there remains a notable gap in the literature. This gap



For peer review only - http://bmjopen.bmj.com/site/about/guidelines.xhtml

pertains to the absence of a comprehensive multivariable prediction model that integrates all these factors, providing a holistic assessment of multimorbidity risk. Our study seeks to address this gap by developing and validating a novel risk assessment model that encompasses a broad spectrum of variables, including those mentioned above. Our aim is to equip individuals with a more accurate and personalized estimate of their risk of developing multimorbidity, contributing to a deeper understanding of this multifaceted health issue.

Wider determinants of health (WDHs) encompass a multitude of social, economic, political, and environmental factors that exert influence on health outcomes across an individual's lifespan. This influential model of health determinants places constitutional factors such as sex, age, and genetics at its core, surrounded by concentric layers that encompass individual lifestyle factors, followed by the broader determinants(19). While the core attributes remain relatively fixed, the determinants become more modifiable as the layers extend outward. Existing research has identified that individual lifestyle factors significantly contribute to multimorbidity among older adults. Chudasama also found that adopting a healthier lifestyle was associated with longer life expectancy for middle-aged adults, regardless of the presence of multimorbidity.(4).

In the context of the WDHs framework, individual behaviors constitute the innermost layer, presenting opportunities for modification, particularly through self-health management(20-22). The accurate assessment of one's risk of multimorbidity and the identification of potential risk factors represent critical initial steps in the journey of self-management. Therefore, the development of a user-friendly tool to assist



For peer review only - http://bmjopen.bmj.com/site/about/guidelines.xhtml

individuals in estimating their risk of multimorbidity is of paramount significance.

A nomogram is a health risk appraisal model that offers individualized, evidence-based, and highly accurate risk estimation (23, 24). It is easy to use and can facilitate self-management-related decision making. It is a user-friendly tool that facilitates decision-making related to self-management. In this study, we have developed the first nomogram for predicting the risk of multimorbidity among middle-aged and older adults.

## Methods

### Study population

We utilized data from the China Health and Retirement Longitudinal Study (CHARLS), a nationally representative survey of Chinese residents aged 45 years and above. The baseline survey was conducted in 2011 using a multistage probability sampling strategy and probability-proportional-to-size (PPS) sampling technique to ensure national representativeness. Follow-up waves were conducted in 2013, 2015, and 2018. Detailed information regarding the purpose, design, sample, and questionnaires used in CHARLS can be found in other studies(6, 25, 26). For this study, participants below the age of 45 and those with missing values in any variables were excluded from the analysis. The selection process is outlined in Additional file-Figure S1.

### Measurements

### Multimorbidity

In this study, multimorbidity was defined as the presence of two or more chronic non-



For peer review only - http://bmjopen.bmj.com/site/about/guidelines.xhtml

communicable diseases, whether physical or psychological(6, 27). We assessed multimorbidity by examining the presence of 14 specific non-communicable diseases. Physical chronic non-communicable diseases encompassed diagnosed conditions such as hypertension, dyslipidemia, diabetes, cancer, chronic lung disease, liver disease, heart disease, stroke, kidney disease, digestive disease, asthma, and arthritis. Psychological chronic non-communicable diseases included diagnosed emotional, nervous, or psychiatric problems, as well as memory-related diseases (all diseases were self-reported and diagnosed chronic conditions). To identify individuals with multimorbidity, we calculated the number of chronic diseases present for each participant. The outcome was the time to multimorbidity.

**The modifiable lifestyles**

This study included four well-known healthy lifestyle factors(4): physical activity (PA), smoking, alcohol consumption and diet behavior. Besides, sleep and social activity were included in this study.

The physical activity questionnaire used in CHARLS closely resembled the short version of the International Physical Activity Questionnaire (IPAQ)(28). However, some differences existed between CHARLS and IPAQ, such as assessing PA for a "usual week" instead of "the last 7 days" and lacking information on sedentariness. Additionally, instead of continuous values, four discrete time durations ("< 30 minutes" "≥30 minutes" "< 4 hours" and "≥ 4 hours") were collected(29). We calculated the median score for each intensity level and summed the number of different intensity levels using the metabolic equivalent (MET) as a reference. The weight of each



For peer review only - http://bmjopen.bmj.com/site/about/guidelines.xhtml

intensity level was derived from the IPAQ scoring protocol. Detailed information on PA and its calculation process can be found in the study by Li(26). The total weekly PA (MET-minutes/week) was calculated by multiplying the frequency, duration, and MET values. According to IPAQ, a minimum total PA of at least 600 MET-minutes/week was defined as regular PA, while <600 MET-minutes/week indicated a lack of regular PA.

Smoking was categorized as No (not current smoker) and Yes (current smoker) at the time of assessment. Alcohol consumption status was divided into 2 groups: No (Did not drink in the past 12 months or drinking frequency is less than weekly) and Yes (others). Regular eating behavior was determined based on the frequency of meals per day, with having three meals on time considered as regular eating.

Based on studies conducted in developed countries, respondents' total sleep duration was classified into five categories: <6 hours, 6 to <7 hours, 7 to <8 hours, 8 to <9 hours, and ≥9 hours(30, 31). According to the Healthy China initiative (2019-2030), the length of night-time sleep ≥ 7h was defined as enough sleep in this study (32). Social activity was categorized as "No" and "Yes" based on engagement in social activities within the past 12 months.

**Demographic characteristics**

Demographic characteristics included age, sex (male and female), marital status (others and married), residency (others and rural), education (primary education and below, secondary education and above), and BMI scores.

The covariates, including demographic characteristics and modifiable lifestyles factors



For peer review only - http://bmjopen.bmj.com/site/about/guidelines.xhtml

were gathered by baseline questionnaire.

**Depression**

Depression was assessed using the Chinese version of the Center for Epidemiological Studies Depression scale (CES-D-10)(33). The CES-D-10 contains of 10 items with 4 response options: rare, some days (1–2 days), occasionally (3–4 days) and most of the time (5–7 days)(25). The scales for each of the 10 items were adjusted to 0, 1, 2 and 3, resulting in a CES-D-10 score ranging from 0 to 30, with higher scores indicating more negative feelings during the past week(34, 35).

**Statistical analysis**

The participants were randomly divided into a derivation set and a validation set at a ratio of 7:3. Participant characteristics, such as age and BMI, were summarized as mean ± SD and counts with proportions for categorical features. Cox proportional hazard models were used to estimate the associations between modifiable lifestyles (including physical activity, smoking, alcohol consumption, and diet behavior), depression, and other identified risk factors with the development of multimorbidity in middle-aged and older adults. Hazard ratios (HR) and 95% confidence intervals (95% CI) were reported for the total population. Factors with a significant level of less than 0.05 in the univariable regression model were entered into the multivariable Cox proportional hazard model for adjustment.

A nomogram was developed based on the results of the multivariable cox proportional hazard model in the derivation set. The nomogram assigns risk points to each variable by proportionally converting regression coefficients to a 0–100-point scale. The



For peer review only - http://bmjopen.bmj.com/site/about/guidelines.xhtml

variable with the highest absolute value of the β coefficient is assigned 100 points. The risk points for other variables are calculated based on the ratio of risk points to the *β* coefficient of the highest variable. A prognostic index (PI) was calculated by summing the risk points corresponding to each weighted covariate. The nomogram was validated using the concordance index (C-index) calculated through 1000-fold bootstrap resampling to reduce overfit bias. The developed nomogram was then applied to the validation set. Model performance was further evaluated using a calibration curve, which superimposes both datasets for visual comparison of discrimination. All analyses were performed using R, version 3.0, $P<0.05$ was considered to indicate statistical significance.

**Patient and public involvement:** The public were not involved in the design, or conduct, or reporting, or dissemination plans of this research.

## Results

### Baseline characteristics

A total of 7,735 participants were included in this study, with 5,449 individuals in the derivation set and 2,286 in the validation set. The baseline characteristics of the study sample are presented in Table 1. The average age of participants in both datasets was 59.0±9.2 years, and in 2011, 3,726 individuals (48.2%) had at least one chronic disease.

**Table 1 Characteristics of the sample on the baseline-2011**

| Characteristics | $N$ (%) /$S\pm\bar{X}$ | | |
|---|---|---|---|
| | **All** | **Derivation Set** | **Validation Set** |



For peer review only - http://bmjopen.bmj.com/site/about/guidelines.xhtml

| | | | |
|---|---|---|---|
| **Age (y)** | | | |
| <60 | 4387(56.7) | 3039(55.8) | 1348(59.0) |
| ≥60 | 3348(43.3) | 2410(44.2) | 938(41.0) |
| **Sex** | | | |
| Male | 3744(48.4) | 2684(49.3) | 1060(46.4) |
| Female | 3991(51.6) | 2765(50.7) | 1226(53.6) |
| **Hukou** | | | |
| Others | 6423(83.0) | 4504(82.7) | 1919(83.9) |
| Rural | 1312(17.0) | 945(17.3) | 367(16.1) |
| **Education** | | | |
| Primary education and below | 5296(68.5) | 3720(68.3) | 1576(68.9) |
| Secondary education and above | 2439(31.5) | 1729(31.7) | 710(31.1) |
| **Martial** | | | |
| Others | 1304(16.9) | 910(16.7) | 394(17.2) |
| Married | 6431(83.1) | 4539(83.3) | 1892(82.8) |
| **Chronic Disease** | | | |
| No | 4009(51.8) | 2817(51.7) | 1192(52.1) |
| Yes | 3726(48.2) | 2632(48.3) | 1094(47.9) |
| **Sleep (h)** | | | |
| <7 | 3803(49.2) | 2701(49.6) | 1102(48.2) |
| ≥7 | 3932(50.8) | 2748(50.4) | 1184(51.8) |
| **Social Activity** | | | |
| No | 3864(50.0) | 2747(50.4) | 1117(48.9) |
| Yes | 3871(50.0) | 2702(49.6) | 1169(51.1) |
| **Regular PA** | | | |
| No | 3422(44.2) | 2407(44.2) | 1015(44.4) |
| Yes | 4313(55.8) | 3042(55.8) | 1271(55.6) |
| **Regular Eating** | | | |



For peer review only - http://bmjopen.bmj.com/site/about/guidelines.xhtml

For peer review only

| | | | |
|---|---|---|---|
| No | 1009(13.0) | 707(13.0) | 302(13.2) |
| Yes | 6726(87.0) | 4742(87.0) | 1984(86.8) |
| **Smoke** | | | |
| No | 3379(43.7) | 2390(43.9) | 989(43.3) |
| Yes | 4356(56.3) | 3059(56.1) | 1297(56.7) |
| **Drink** | | | |
| No | 3354(43.4) | 2387(43.8) | 967(42.3) |
| Yes | 4381(56.6) | 3062(56.2) | 1319(57.7) |
| BMI | 23.32±3.69 | 23.31±3.72 | 23.35±3.62 |
| **Depression** | 9.13±4.57 | 9.10±4.56 | 9.21±4.60 |

## Prevalence of multimorbidity

In 2018, a total of 3,576 participants (46.2%) were found to have multimorbidity. Among these individuals, the prevalence of multimorbidity was higher among older adults compared to those under the age of 60 (51.0% vs. 42.6%). Additionally, females exhibited a higher prevalence of multimorbidity compared to males (48.5% vs. 43.8%). Moreover, married individuals (45.7% vs. 48.6%) and those with higher education (42.1% vs. 48.1%) had a lower prevalence of multimorbidity compared to others (Figure 1).

**Figure 1. Prevalence of multimorbidity in the population**

## Predictor factors of multimorbidity

The results of the univariable Cox proportional hazard model for the entire dataset (Additional file-Table S1) indicated that several factors were significantly associated with an increased risk of multimorbidity. These factors included older age, being female (HR 1.16, 95% CI 1.08-1.24), having existing chronic disease (HR 2.85, 95%CI 2.66-



For peer review only - http://bmjopen.bmj.com/site/about/guidelines.xhtml

3.06), and higher depression scores (HR 1.04, 95%CI 1.03-1.05). Conversely, a higher level of education (HR 0.83, 95%CI 0.77-0.90), marital status (HR 0.91, 95%CI 0.83-0.99), higher BMI scores (HR 1.04, 95%CI 1.03-1.05), smoking (HR 1.46, 95%CI 1.36-1.56), drinking (1.46, 95%CI 1.36-1.56), getting at least 7 hours of sleep (HR 0.70, 95%CI 0.66-0.75), engaging in regular PA (HR 0.83, 95%CI 0.77-0.88) were associated with a decreased risk of multimorbidity.

The multivariable Cox proportional hazard models incorporated eleven significant variables identified through univariate analysis. Variable selection was performed using the backward selection method. In comparison to individuals under the age of 50, those in the age groups of 50-59 (HR 1.18, 95%CI 1.04-1.33), 60-69 (HR 1.44, 95%CI 1.27-1.64), and 70-79 (HR 1.23, 95%CI 1.04-1.45) exhibit a higher risk of multimorbidity. Furthermore, being female (HR 1.23, 95%CI 1.10-1.36), having a history of chronic disease (HR 2.59, 95%CI 2.38-2.82), getting at least 7 hours of sleep (HR 0.78, 95%CI 0.72-0.85), engaging in regular PA (HR 0.88, 95%CI 0.81-0.95), drinking (HR 1.27 95%CI 1.16-1.39), smoking (HR 1.40, 95%CI 1.26-1.53), higher BMI (HR 1.04, 95%CI 1.03-1.05) and increased depression scores (HR 1.02, 95%CI 1.01-1.03) were all found to be associated with multimorbidity in derivation set (Table 2).

**Table 2 Factors associated with the risk of multimorbidity (multivariable cox proportional hazard model)**

| Factors | Derivation Set | | | Validation Set | | |
|---|---|---|---|---|---|---|
| | *HR* | *95%CI* | *P* | *HR* | *95%CI* | *P* |
| Age(<50) | | | | | | |
| Age(50-59) | 1.18 | 1.04-1.33 | 0.008 | 1.20 | 1.01-1.43 | 0.049 |



For peer review only - http://bmjopen.bmj.com/site/about/guidelines.xhtml

| | | | | | | |
|---|---|---|---|---|---|---|
| Age(60-69) | 1.44 | 1.27-1.64 | <0.001 | 1.26 | 1.04-1.52 | 0.018 |
| Age(70-79) | 1.23 | 1.04-1.45 | 0.014 | 1.24 | 0.99-1.56 | 0.060 |
| Age(>=80) | 0.92 | 0.70-1.23 | 0.579 | 1.01 | 0.66-1.55 | 0.964 |
| Sex (Male) | | | | | | |
| Female | 1.23 | 1.10-1.36 | <0.001 | 1.43 | 1.22-1.67 | <0.001 |
| Chronic disease (No) | | | | | | |
| Yes | 2.59 | 2.38-2.82 | <0.001 | 2.70 | 2.37-3.06 | <0.001 |
| Sleep (<7h) | | | | | | |
| >=7h | 0.78 | 0.72-0.85 | <0.001 | 0.74 | 0.65-0.83 | <0.001 |
| Regular PA (No) | | | | | | |
| Yes | 0.88 | 0.81-0.95 | 0.001 | 0.88 | 0.77-0.99 | 0.039 |
| Drink (No) | | | | | | |
| Yes | 1.27 | 1.16-1.39 | <0.001 | 1.35 | 1.17-1.56 | <0.001 |
| Smoke (No) | | | | | | |
| Yes | 1.40 | 1.26-1.53 | <0.001 | 1.57 | 1.34-1.84 | <0.001 |
| BMI | 1.04 | 1.03-1.05 | <0.001 | 1.02 | 1.01-1.03 | 0.001 |
| Depression | 1.02 | 1.01-1.03 | <0.001 | 1.02 | 1.01-1.04 | 0.001 |

**Development and validation of an multimorbidity -predicting nomogram**

The PI was calculated based on the hazard ratio associated with the identified risk factors for multimorbidity. The nomogram was constructed using these results, with the BMI variable assigned a total scale of 100 and a range of 5 to 50. The risk score for BMI was determined to be 2.2. The risk scores for the other risk factors of multimorbidity were calculated proportionally to the β coefficient of BMI. So the PI=(0.3*I[age-45])+(14.3*I[female]) +(56.9*I[chronic disease])+(14.8*I[1-sleep>=7h)+(6.4*I[1-regular PA])+(13.7*I[drink])+(19.1*I[smoke])+(2.2*BMI)+

For peer review only

(1.4*depression), where I[ ]denotes the indicator function equal to 1 if the condition in parenthesis is met and 0 otherwise, except age. Based upon these findings, a nomogram was configured (Figure 2).

**Figure 2. The nomogram for multimorbidity risk prediction. Note: Draw a line perpendicular from the corresponding axis of each risk factor until it reaches the line labeled "POINTS". Sum up the number of points for all risk factors then draw a line descending from the labeled "TOTAL POINTS" until it intercepts each of the survival axes to determine 2-, 4-, and 7-year survival probabilities, multimorbidity probability=1- survival probability.**

The resulting nomogram was internally validated using the bootstrap validation method, and it demonstrated good accuracy in estimating the risk of multimorbidity, with a bootstrap-corrected C-index of 0.70 (95%CI 0.69-0.71, $P$=0.006) in the derivation set. Calibration plots also indicated good agreement between the risk estimation by the nomogram and the diagnosis of doctors, as depicted in Figure 2. When the estimates from the derivation set were applied to the validation set, a similar bootstrap-corrected C-index of 0.71 (95%CI 0.70-0.73, $P$=0.008) was obtained, along with a well-calibrated risk estimation curve (Figure 3).

**Figure 3. The predictive performance of the nomogram in estimating the risk of multimorbidity**

## Discussion



For peer review only - http://bmjopen.bmj.com/site/about/guidelines.xhtml

In this study, we observed a prevalence of multimorbidity of 46.2% among middle-aged and older adults. The prevalence of multimorbidity was higher among individuals with one chronic disease (62.8%) compared to those without chronic diseases (30.8%) at baseline. This result is consistent with the probability range of multimorbidity reported in a systematic study of the elderly(8). Although there were some differences in the estimation of multimorbidity compared to other studies, such as the number of included chronic diseases(36, 37), and the methods used to collect information(38). Our results confirmed the significant impact of multimorbidity on middle-aged and older adults, particularly among those with one chronic disease.

Our findings provide sufficient evidence for the association between older age and multimorbidity, which is consistent with similar trends observed in countries such as Singapore(39), Ireland, and Scotland(27). The prevalence of multimorbidity among females is higher than males. Additionally, we found a higher prevalence of multimorbidity among females compared to males, which is supported by studies from various countries indicating that older men have a lower risk of multimorbidity than their female counterparts(27, 38). However, a study by Lian found that the onset of multimorbidity occurs at an earlier age in males than in females(39). This is also a new idea to understand the sex-differ in multimorbidity, but our study only included middle-aged and older adults aged over 45 years, we cannot evaluate this result of Lian. Future research could explore the prevalence and different combinations of chronic conditions in people with multimorbidity across various age and sex groups.

Existing studies have established a link between short sleep duration and



For peer review only - http://bmjopen.bmj.com/site/about/guidelines.xhtml

multimorbidity. Experimental evidence confirms the deleterious effects of sleep deprivation on endocrine, immune, neurovitality and inflammatory pathways(40). For instance, Maria Ruiz-Castell found an association between short sleep duration and the number of chronic conditions(41). Helbig also observed a significant positive relationship between short sleep duration and multimorbidity among women(42). High-risk lifestyles, such as smoking, excessive alcohol consumption, poor diet, physical inactivity, and unhealthy body shape, have also been confirmed as contributing factors to multimorbidity(43). Smoking and excessive alcohol consumption remain leading risk factors for early death and disability globally(44, 45). In our study, we found that smoking and drinking increased the risk of multimorbidity, with the highest risk index among all unhealthy behaviors. The expansion of tobacco and alcohol control measures remains a significant public health priority worldwide. Mika conducted an observational study using data from two Finnish cohort studies comprising 614,014 adults, and the results showed that obesity is a significant factor in multimorbidity(46). Similarly, our study found that higher BMI was associated with an increased risk of multimorbidity among middle-aged and older adults. There exists a bidirectional association between depression and multimorbidity(16, 47). Depression increases the risk of multimorbidity, while having multimorbidity also raises the risk of depression. Our study found the risk of multimorbidity for middle-aged and older adults with higher depression scores.

Based on our results, we developed a user-friendly nomogram model for predicting the risk of multimorbidity. One of the most appealing aspects of our nomogram model is



For peer review only - http://bmjopen.bmj.com/site/about/guidelines.xhtml

its home applicability and ease of use by individuals. For example, a 50-year-old married male with primary education, one chronic disease, a history of smoking and excessive alcohol consumption, irregular physical activity, 8 hours of sleep, a depression score of 16, and a BMI of 24.9 would have a total risk score of 219.3 points. This corresponds to a 2-, 4-, and 7-year probability of multimorbidity of 32%, 71%, and <10%, respectively. Based on the calculated results, individuals can develop self-management strategies to reduce their risk of multimorbidity.

**Limitation**

Our study had several limitations. First, the model was constructed based on behavioral and household-level risk factors, limiting its applicability to clinical prediction. Additionally, in this study, there is a large proportion of missing data for some important variables, so we have used a culling method to deal with the missing values. As a result, this may lead to sample bias and the extrapolation of the model needs to be careful. We also need to validate the model using external data.

## Conclusions

This study confirms the severity of multimorbidity among middle-aged and older adults, particularly among those who already have one chronic disease. Age showed a significant correlation with multimorbidity, and the prevalence of multimorbidity was higher in females compared to males. In addition, insufficient sleep, smoking, drinking, obesity, and depressive symptoms were also associated with multimorbidity. Based on these findings, we developed a user-friendly nomogram model to predict the risk of



For peer review only - http://bmjopen.bmj.com/site/about/guidelines.xhtml

multimorbidity in middle-aged and older adults. Our research not only builds upon the existing body of knowledge but also introduces a novel and comprehensive approach to assessing multimorbidity risk, which is of significant clinical and public health relevance. The multivariable prediction model provides valuable tools for healthcare professionals to manage multimorbidity.

## Ethics statements

### Patient consent for publication

Not applicable.

### Ethics approval

The Biomedical Ethics Review Committee of Peking University approved CHARLS, and all participants were required to provide written informed consent. The ethical approval number was IRB00001052-11015.

## Supplementary materials

Additional file.

## Footnotes

**Contributors:** ZCC and TF conceived the study. ZX and ZCC were major contributors in writing the manuscript. XBL, XSJ, LXR, analyzed and interpreted the patient data. CYM SL and LXY edited and contributed content to the final draft. All authors read and approved the final manuscript.



For peer review only

For peer review only - http://bmjopen.bmj.com/site/about/guidelines.xhtml

**Funding:** This study was funded by the Guangdong Basic and Applied Basic Research Foundation (No.2022A1515110295, No.2022A1515011591), National Nature Science Foundation of China (No.72274091), China Postdoctoral Science Foundation (No. 2022M721539), Guangdong Philosophy and Social Science Foundation (No. GD23CGL06).

**Competing interests:** The authors declare that they have no competing interests.

**Availability of data and materials:** The datasets analysed during the current study are available from the corresponding author on reasonable request.

## References

1. Fortin M, Bravo G, Hudon C, Vanasse A, Lapointe L. Prevalence of multimorbidity among adults seen in family practice. Ann Fam Med. 2005;3(3):223-8.

2. Jiao DD, Watanabe K, Sawada Y, Tanaka E, Watanabe T, Tomisaki E, et al. Multimorbidity and functional limitation: the role of social relationships. Arch Gerontol Geriat. 2021;92.

3. Nicholson K, Griffith LE, Sohel N, Raina P. Examining early and late onset of multimorbidity in the Canadian Longitudinal Study on Aging. J Am Geriatr Soc. 2021.

4. Chudasama YV, Khunti K, Gillies CL, Dhalwani NN, Davies MJ, Yates T, et al. Healthy lifestyle and life expectancy in people with multimorbidity in the UK Biobank: A longitudinal cohort study. PLoS Med. 2020;17(9):e1003332.

5. Ding D, Lawson KD, Kolbe-Alexander TL, Finkelstein EA, Katzmarzyk PT, van Mechelen W, et al. The economic burden of physical inactivity: a global analysis of



For peer review only - http://bmjopen.bmj.com/site/about/guidelines.xhtml

major non-communicable diseases. Lancet. 2016;388(10051):1311-24.

6. Zhao Y, Atun R, Oldenburg B, McPake B, Tang S, Mercer SW, et al. Physical multimorbidity, health service use, and catastrophic health expenditure by socioeconomic groups in China: an analysis of population-based panel data. The Lancet Global Health. 2020;8(6):e840-e9.

7. Zheng X, Cui Y, Xue Y, Shi L, Guo Y, Dong F, Zhang C. Adverse childhood experiences in depression and the mediating role of multimorbidity in mid-late life: A nationwide longitudinal study. J Affect Disord. 2022 Mar 15;301:217-224.

8. Marengoni A, Angleman S, Melis R, Mangialasche F, Karp A, Garmen A, et al. Aging with multimorbidity: A systematic review of the literature. Ageing Res Rev. 2011;10(4):430-9.

9. Xue B, Xue Y, Dong F, Zheng X, Shi L, Xiao S, Zhang J, Ou W, Wang Q, Zhang C. The impact of socioeconomic status and sleep quality on the prevalence of multimorbidity in older adults. Front Public Health. 2022 Sep 26;10:959700.

10. Olaya B, Domenech-Abella J, Moneta MV, Lara E, Caballero FF, Rico-Uribed LA, et al. All-cause mortality and multimorbidity in older adults: The role of social support and loneliness. Exp Gerontol. 2017;99:120-6.

11. Bertuol C, Tozetto WR, Streb AR, Del Duca GF. Combined relationship of physical inactivity and sedentary behaviour with the prevalence of noncommunicable chronic diseases: data from 52,675 Brazilian adults and elderly. Eur J Sport Sci. 2021.

12. Lindstrom J, Uusitupa M. Lifestyle intervention, diabetes, and cardiovascular disease. Lancet. 2008;371(9626):1731-3.



For peer review only - http://bmjopen.bmj.com/site/about/guidelines.xhtml

13. Elwood P, Galante J, Pickering J, Palmer S, Bayer A, Ben-Shlomo Y, et al. Healthy Lifestyles Reduce the Incidence of Chronic Diseases and Dementia: Evidence from the Caerphilly Cohort Study. Plos One. 2013;8(12).

14. Hu FB, Liu Y, Willett WC. Preventing chronic diseases by promoting healthy diet and lifestyle: public policy implications for China. Obes Rev. 2011;12(7):552-9.

15. Fortin M, Haggerty J, Almirall J, Bouhali T, Sasseville M, Lemieux M. Lifestyle factors and multimorbidity: a cross sectional study. BMC Public Health. 2014;14:686.

16. Birk JL, Kronish IM, Moise N, Falzon L, Yoon S, Davidson KW. Depression and Multimorbidity: Considering Temporal Characteristics of the Associations Between Depression and Multiple Chronic Diseases. Health Psychology. 2019;38(9):802-11.

17. Meng L, Chen DM, Yang Y, Zheng Y, Hui RT. Depression increases the risk of hypertension incidence: a meta-analysis of prospective cohort studies. J Hypertens. 2012;30(5):842-51.

18. Qiao YN, Liu SY, Zhang YX, Wu Y, Shen YP, Ke CF. Bidirectional association between depression and multimorbidity in middle-aged and elderly Chinese adults: a longitudinal cohort study. Aging & Mental Health. 2021.

19. Bambra C, Gibson M, Sowden A, Wright K, Whitehead M, Petticrew M. Tackling the wider social determinants of health and health inequalities: evidence from systematic reviews. J Epidemiol Commun H. 2010;64(4):284-91.

20. Chodosh J, Morton SC, Mojica W, Maglione M, Suttorp MJ, Hilton L, et al. Meta-analysis: Chronic disease self-management programs for older adults. Ann Intern Med. 2005;143(6):427-38.



For peer review only

For peer review only - http://bmjopen.bmj.com/site/about/guidelines.xhtml

21. Warsi A, Wang PS, LaValley MP, Avorn J, Solomon DH. Self-management education programs in chronic disease - A systematic review and methodological critique of the literature. Arch Intern Med. 2004;164(15):1641-9.

22. Li WY, Chiu FC, Zeng JK, Li YW, Huang SH, Yeh HC, et al. Mobile Health App With Social Media to Support Self-Management for Patients With Chronic Kidney Disease: Prospective Randomized Controlled Study. Journal of Medical Internet Research. 2020;22(12).

23. Lei Z, Li J, Wu D, Xia Y, Wang Q, Si A, et al. Nomogram for Preoperative Estimation of Microvascular Invasion Risk in Hepatitis B Virus-Related Hepatocellular Carcinoma Within the Milan Criteria. JAMA Surg. 2016;151(4):356-63.

24. B OH, Gransar H, Callister T, Shaw LJ, Schulman-Marcus J, Stuijfzand WJ, et al. Development and Validation of a Simple-to-Use Nomogram for Predicting 5-, 10-, and 15-Year Survival in Asymptomatic Adults Undergoing Coronary Artery Calcium Scoring. JACC Cardiovasc Imaging. 2018;11(3):450-8.

25. Jing R, Xu T, Rong H, Lai X, Fang H. Longitudinal Association Between Sleep Duration and Depressive Symptoms in Chinese Elderly. Nat Sci Sleep. 2020;12:737-47.

26. Li X, Zhang W, Zhang W, Tao K, Ni W, Wang K, et al. Level of physical activity among middle-aged and older Chinese people: evidence from the China health and retirement longitudinal study. BMC Public Health. 2020;20(1):1682.

27. Barnett K, Mercer SW, Norbury M, Watt G, Wyke S, Guthrie B. Epidemiology of multimorbidity and implications for health care, research, and medical education: a



For peer review only

For peer review only - http://bmjopen.bmj.com/site/about/guidelines.xhtml

cross-sectional study. Lancet. 2012;380(9836):37-43.

28. Craig CL, Marshall AL, Sjostrom M, Bauman AE, Booth ML, Ainsworth BE, et al. International physical activity questionnaire: 12-country reliability and validity. Med Sci Sports Exerc. 2003;35(8):1381-95.

29. Zhao Y, Hu Y, Smith JP, Strauss J, Yang G. Cohort profile: the China Health and Retirement Longitudinal Study (CHARLS). Int J Epidemiol. 2014;43(1):61-8.

30. Yang L, Yang H, He M, Pan A, Li X, Min X, et al. Longer Sleep Duration and Midday Napping Are Associated with a Higher Risk of CHD Incidence in Middle-Aged and Older Chinese: the Dongfeng-Tongji Cohort Study. Sleep. 2016;39(3):645-52.

31. Paudel M, Taylor BC, Ancoli-Israel S, Blackwell T, Maglione JE, Stone K, et al. Sleep Disturbances and Risk of Depression in Older Men. Sleep. 2013;36(7):1033-40.

32. China TPsRo. Healthy China initiative (2019-2030) 2019 [Available from: http://www.gov.cn/xinwen/2019-07/15/content_5409694.htm.

33. Jin Y, Luo Y, He P. Hypertension, socioeconomic status and depressive symptoms in Chinese middle-aged and older adults: Findings from the China health and retirement longitudinal study. J Affect Disord. 2019;252:237-44.

34. Wang RX, Chen Z, Zhou YJ, Shen LN, Zhang Z, Wu X. Melancholy or mahjong? Diversity, frequency, type, and rural-urban divide of social participation and depression in middle- and old-aged Chinese: A fixed-effects analysis. Soc Sci Med. 2019;238.

35. Chen Z, Ying J, Ingles J, Zhang D, Rajbhandari-Thapa J, Wang R, et al. Gender differential impact of bereavement on health outcomes: evidence from the China Health and Retirement Longitudinal Study, 2011-2015. BMC Psychiatry. 2020;20(1):514.



For peer review only - http://bmjopen.bmj.com/site/about/guidelines.xhtml

36. Diederichs C, Berger K, Bartels DB. The Measurement of Multiple Chronic Diseases-A Systematic Review on Existing Multimorbidity Indices. J Gerontol a-Biol. 2011;66(3):301-11.

37. Guisado-Clavero M, Roso-Llorach A, Lopez-Jimenez T, Pons-Vigues M, Foguet-Boreu Q, Munoz MA, et al. Multimorbidity patterns in the elderly: a prospective cohort study with cluster analysis. Bmc Geriatrics. 2018;18.

38. Hewitt J, McCormack C, Tay HS, Greig M, Law J, Tay A, et al. Prevalence of multimorbidity and its association with outcomes in older emergency general surgical patients: an observational study. Bmj Open. 2016;6(3).

39. Low LL, Kwan YH, Ko MSM, Yeam CT, Lee VSY, Tan WB, et al. Epidemiologic Characteristics of Multimorbidity and Sociodemographic Factors Associated With Multimorbidity in a Rapidly Aging Asian Country. JAMA Netw Open. 2019;2(11):e1915245.

40. Ferrie JE, Kivimaki M, Akbaraly TN, Singh-Manoux A, Miller MA, Gimeno D, et al. Associations Between Change in Sleep Duration and Inflammation: Findings on C-reactive Protein and Interleukin 6 in the Whitehall II Study. American Journal of Epidemiology. 2013;178(6):956-61.

41. Ruiz-Castell M, Makovski TT, Bocquet V, Stranges S. Sleep duration and multimorbidity in Luxembourg: results from the European Health Examination Survey in Luxembourg, 2013-2015. BMJ Open. 2019;9(8):e026942.

42. Helbig AK, Stockl D, Heier M, Thorand B, Schulz H, Peters A, et al. Relationship between sleep disturbances and multimorbidity among community-dwelling men and



For peer review only - http://bmjopen.bmj.com/site/about/guidelines.xhtml

women aged 65-93 years: results from the KORA Age Study. Sleep Med. 2017;33:151-9.

43. Han Y, Hu Y, Yu C, Guo Y, Pei P, Yang L, et al. Lifestyle, cardiometabolic disease, and multimorbidity in a prospective Chinese study. Eur Heart J. 2021;42(34):3374-84.

44. Beaglehole R, Bates C, Youdan B, Bonita R. Nicotine without smoke: fighting the tobacco epidemic with harm reduction. Lancet. 2019;394(10200):718-20.

45. Mohammad KA, Salomon JA. Smoking prevalence and attributable disease burden in 195 countries and territories, 1990-2015: a systematic analysis from the Global Burden of Disease Study 2015 (vol 389, pg 1885, 2015). Lancet. 2017;390(10103):1644-.

46. Kivimaki M, Strandberg T, Pentti J, Nyberg ST, Frank P, Jokela M, et al. Body-mass index and risk of obesity-related complex multimorbidity: an observational multicohort study. Lancet Diabetes Endocrinol. 2022;10(4):253-63.

47. Huang CQ, Dong BR, Lu ZC, Yue JR, Liu QX. Chronic diseases and risk for depression in old age: A meta-analysis of published literature. Ageing Res Rev. 2010;9(2):131-41.

For peer review only



For peer review only - http://bmjopen.bmj.com/site/about/guidelines.xhtml

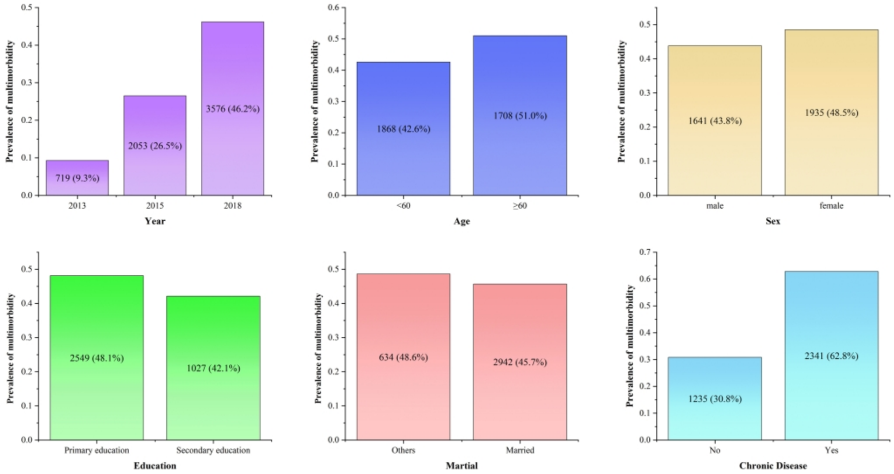


Figure 1. Prevalence of multimorbidity in the population.

338x179mm (300 x 300 DPI)

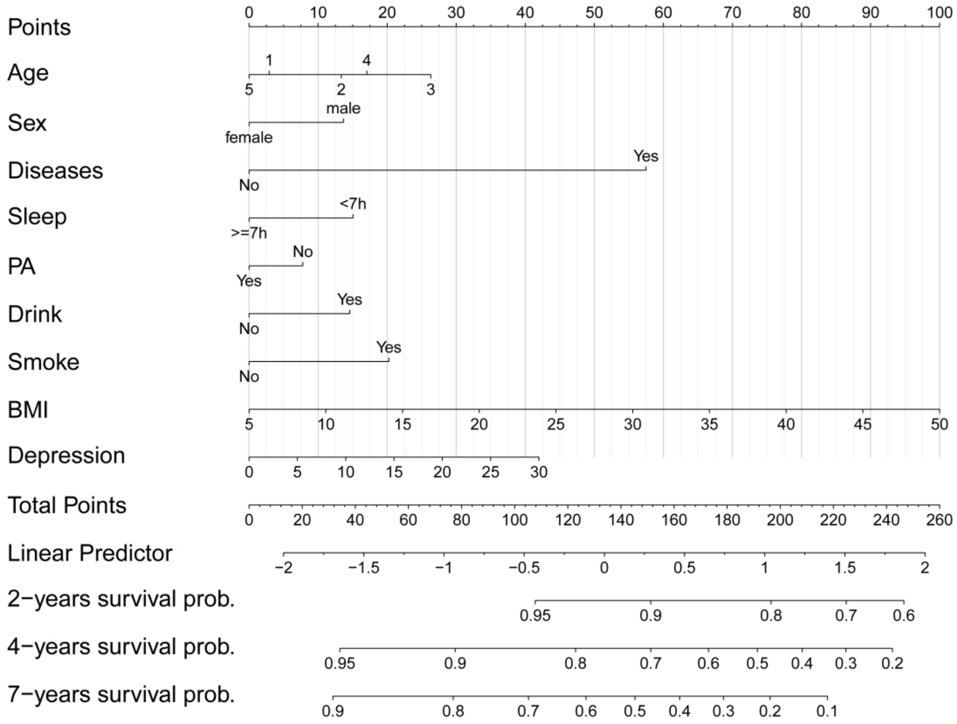


Figure 2. The nomogram for multimorbidity risk prediction. Note: Draw a line perpendicular from the corresponding axis of each risk factor until it reaches the line labeled “POINTS”. Sum up the number of points for all risk factors then draw a line descending from the labeled “TOTAL POINTS” until it intercepts each of the survival axes to determine 2-, 4-, and 7-year survival probabilities, multimorbidity probability=1- survival probability.

728x529mm (118 x 118 DPI)

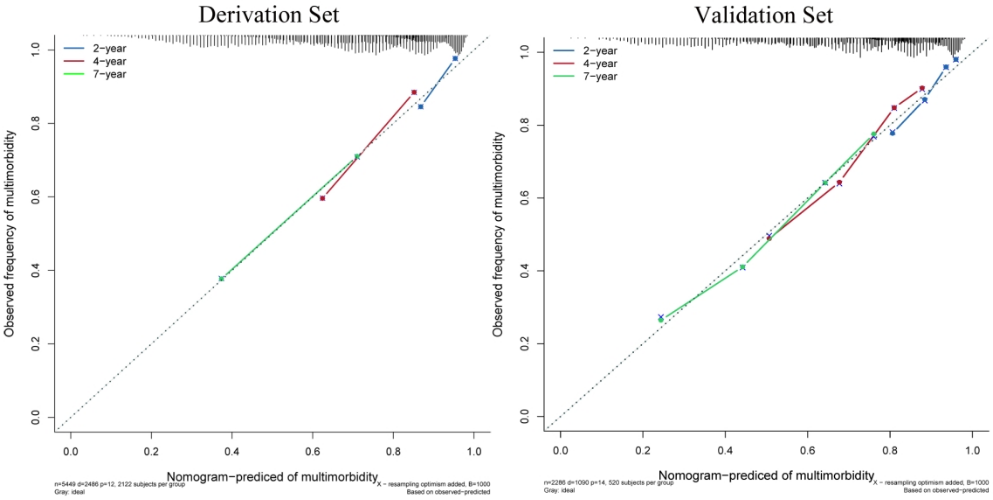


Figure 3. The predictive performance of the nomogram in estimating the risk of multimorbidity

406x210mm (118 x 118 DPI)

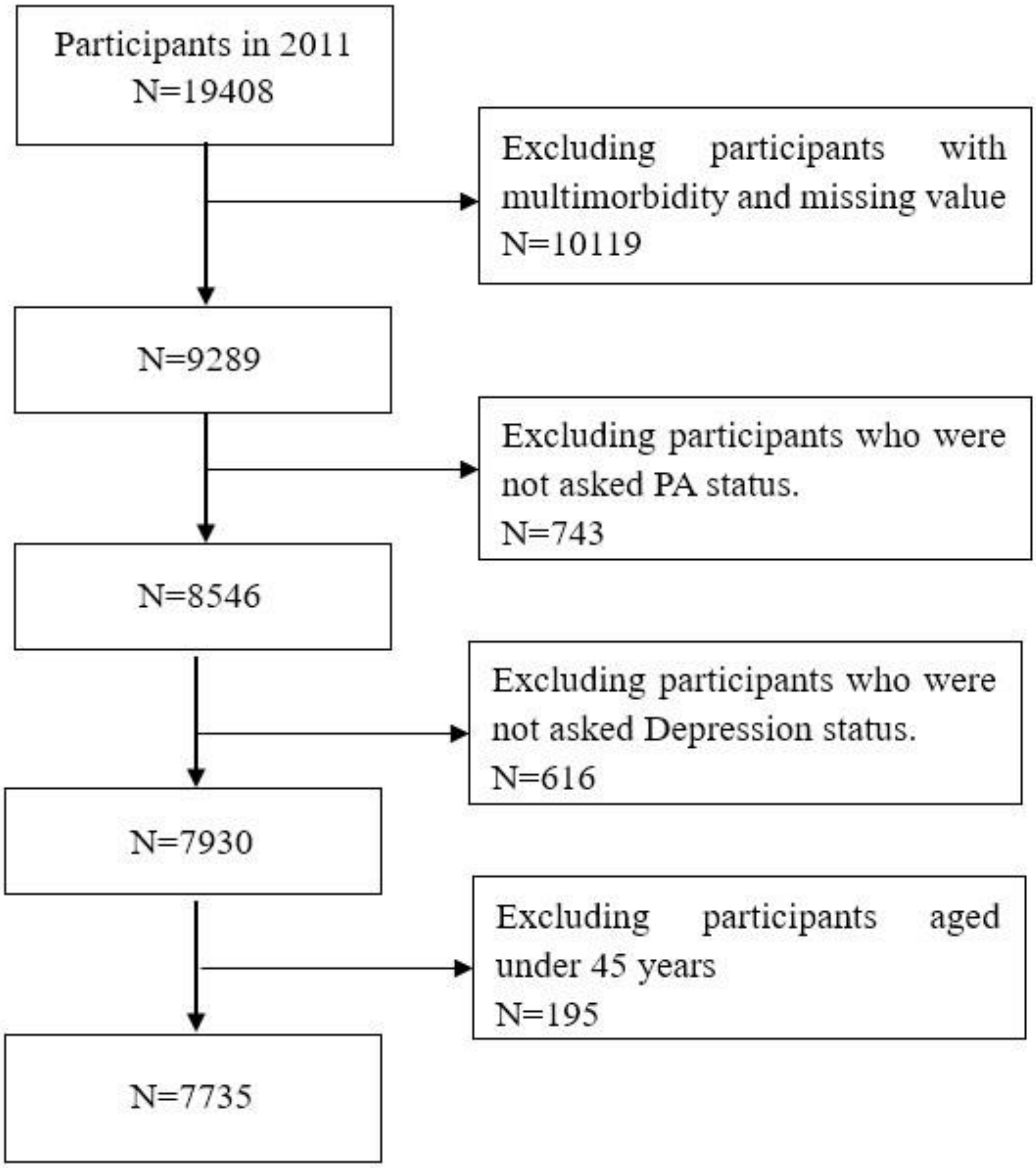


**Figure S1. Flow diagram of Sample selection**

**Table S1 Factors associated with the risk of multimorbidity (univariable cox proportional hazard models, N=7735)**

| Characteristics | | *HR* | *95%CI* | *P* |
|---|---|---|---|---|
| **Age (y)** | <50* | | | |
| | 50-69 | 1.25 | 1.13-1.39 | <0.001 |
| | 60-69 | 1.58 | 1.42-1.74 | <0.001 |
| | 70-79 | 1.41 | 1.25-1.60 | <0.001 |
| | >=80 | 1.12 | 0.90-1.41 | 0.311 |
| **Sex** | Male* | | | |
| | Female | 1.16 | 1.08-1.24 | <0.001 |
| **Hukou** | Rural* | | | |
| | Others | 1.03 | 0.95-1.13 | 0.452 |
| **Education** | Primary* | | | |
| | Secondary | 0.83 | 0.77-0.90 | <0.001 |
| **Marial** | Others* | | | |
| | Married | 0.91 | 0.83-0.99 | 0.027 |
| **Chronic Diseases** | No* | | | |
| | Yes | 2.85 | 2.66-3.06 | <0.001 |
| **Sleep (h)** | <7* | | | |
| | >=7 | 0.70 | 0.66-0.75 | <0.001 |
| **Social Activity** | No* | | | |
| | Yes | 0.96 | 0.9-1.02 | 0.215 |
| **Regular PA** | No* | | | |
| | Yes | 0.83 | 0.77-0.88 | <0.001 |
| **Eating** | No* | | | |
| | Yes | 0.97 | 0.88-1.07 | 0.594 |
| **Smoke** | No* | | | |
| | Yes | 1.46 | 1.36-1.56 | <0.001 |
| **Drink** | No* | | | |
| | Yes | 1.46 | 1.36-1.56 | <0.001 |
| **BMI** | | 1.04 | 1.03-1.05 | <0.001 |
| **Depression** | | 1.04 | 1.03-1.05 | <0.001 |

For peer review only

# TRIPOD Checklist: Prediction Model Development and Validation

| Section/Topic | Item | | Checklist Item | Page |
|---|---|---|---|---|
| **Title and abstract** | | | | |
| Title | 1 | D;V | Identify the study as developing and/or validating a multivariable prediction model, the target population, and the outcome to be predicted. | 1 |
| Abstract | 2 | D;V | Provide a summary of objectives, study design, setting, participants, sample size, predictors, outcome, statistical analysis, results, and conclusions. | 2 |
| **Introduction** | | | | |
| Background and objectives | 3a | D;V | Explain the medical context (including whether diagnostic or prognostic) and rationale for developing or validating the multivariable prediction model, including references to existing models. | 3-5 |
| | 3b | D;V | Specify the objectives, including whether the study describes the development or validation of the model or both. | 5-6 |
| **Methods** | | | | |
| Source of data | 4a | D;V | Describe the study design or source of data (e.g., randomized trial, cohort, or registry data), separately for the development and validation data sets, if applicable. | 6 |
| | 4b | D;V | Specify the key study dates, including start of accrual; end of accrual; and, if applicable, end of follow-up. | 6 |
| S1Participants | 5a | D;V | Specify key elements of the study setting (e.g., primary care, secondary care, general population) including number and location of centres. | 6 |
| | 5b | D;V | Describe eligibility criteria for participants. | 6, Figure S1 |
| | 5c | D;V | Give details of treatments received, if relevant. | |
| Outcome | 6a | D;V | Clearly define the outcome that is predicted by the prediction model, including how and when assessed. | 6-7 |
| | 6b | D;V | Report any actions to blind assessment of the outcome to be predicted. | |
| Predictors | 7a | D;V | Clearly define all predictors used in developing or validating the multivariable prediction model, including how and when they were measured. | 7-9 |
| | 7b | D;V | Report any actions to blind assessment of predictors for the outcome and other predictors. | |
| Sample size | 8 | D;V | Explain how the study size was arrived at. | 6, Figure S1 |
| Missing data | 9 | D;V | Describe how missing data were handled (e.g., complete-case analysis, single imputation, multiple imputation) with details of any imputation method. | 6, Figure S1 |
| Statistical analysis methods | 10a | D | Describe how predictors were handled in the analyses. | 7-9 |
| | 10b | D | Specify type of model, all model-building procedures (including any predictor selection), and method for internal validation. | 9-10 |
| | 10c | V | For validation, describe how the predictions were calculated. | 9-10 |
| | 10d | D;V | Specify all measures used to assess model performance and, if relevant, to compare multiple models. | 9-10 |
| | 10e | V | Describe any model updating (e.g., recalibration) arising from the validation, if done. | 9-10 |
| Risk groups | 11 | D;V | Provide details on how risk groups were created, if done. | |
| Development vs. validation | 12 | V | For validation, identify any differences from the development data in setting, eligibility criteria, outcome, and predictors. | 9-10 |
| **Results** | | | | |
| Participants | 13a | D;V | Describe the flow of participants through the study, including the number of participants with and without the outcome and, if applicable, a summary of the follow-up time. A diagram may be helpful. | Figure S1 |
| | 13b | D;V | Describe the characteristics of the participants (basic demographics, clinical features, available predictors), including the number of participants with missing data for predictors and outcome. | Table 1 |
| | 13c | V | For validation, show a comparison with the development data of the distribution of important variables (demographics, predictors and outcome). | Table 1 |
| Model development | 14a | D | Specify the number of participants and outcome events in each analysis. | 11-12 |
| | 14b | D | If done, report the unadjusted association between each candidate predictor and outcome. | 12-13, Table S1 |
| Model specification | 15a | D | Present the full prediction model to allow predictions for individuals (i.e., all regression coefficients, and model intercept or baseline survival at a given time point). | 13-14, Table 2 |
| | 15b | D | Explain how to the use the prediction model. | 14, Figure 2 |
| Model performance | 16 | D;V | Report performance measures (with CIs) for the prediction model. | 15 |
| Model-updating | 17 | V | If done, report the results from any model updating (i.e., model specification, model performance). | |
| **Discussion** | | | | |
| Limitations | 18 | D;V | Discuss any limitations of the study (such as nonrepresentative sample, few events per predictor, missing data). | 18 |
| Interpretation | 19a | V | For validation, discuss the results with reference to performance in the development data, and any other validation data. | 15-17 |
| | 19b | D;V | Give an overall interpretation of the results, considering objectives, limitations, results from similar studies, and other relevant evidence. | 15-17 |
| Implications | 20 | D;V | Discuss the potential clinical use of the model and implications for future research. | 17-19 |

For peer review only

TRIPOD Checklist: Prediction Model Development and Validation

| Other information | | | | |
|---|---|---|---|---|
| Supplementary information | 21 | D;V | Provide information about the availability of supplementary resources, such as study protocol, Web calculator, and data sets. | |
| Funding | 22 | D;V | Give the source of funding and the role of the funders for the present study. | 19 |

*Items relevant only to the development of a prediction model are denoted by D, items relating solely to a validation of a prediction model are denoted by V, and items relating to both are denoted D;V. We recommend using the TRIPOD Checklist in conjunction with the TRIPOD Explanation and Elaboration document.

For peer review only

BMJ Open

# Development and validation of a multimorbidity risk prediction nomogram among Chinese middle-aged and older adults: a retrospective cohort study

| | |
|---|---|
| Journal: | *BMJ Open* |
| Manuscript ID | bmjopen-2023-077573.R3 |
| Article Type: | Original research |
| Date Submitted by the Author: | 17-Oct-2023 |
| Complete List of Authors: | Zheng, Xiao; Shunde Hospital, Southern Medical University (The First People's Hospital of Shunde, Foshan), Department of Health Management<br>Xue, Benli; Southern Medical University<br>Xiao, Shujuan; Southern Medical University<br>Li, Xinru; Southern Medical University<br>Chen, Yimin; Southern Medical University<br>Shi, Lei; Southern Medical University<br>Liang, Xiaoyan; Shunde Hospital, Southern Medical University (The First People's Hospital of Shunde, Foshan), Department of Health Management<br>Tian, Feng; Shunde Hospital, Southern Medical University (The First People's Hospital of Shunde, Foshan), Department of Health Management<br>Zhang, Chichen; Southern Medical University |
| <b>Primary Subject Heading</b>: | Health services research |
| Secondary Subject Heading: | General practice / Family practice |
| Keywords: | Aging, China, Chronic Disease |
| | |

SCHOLARONE™
Manuscripts

*I, the Submitting Author has the right to grant and does grant on behalf of all authors of the Work (as defined in the below author licence), an exclusive licence and/or a non-exclusive licence for contributions from authors who are: i) UK Crown employees; ii) where BMJ has agreed a CC-BY licence shall apply, and/or iii) in accordance with the terms applicable for US Federal Government officers or employees acting as part of their official duties; on a worldwide, perpetual, irrevocable, royalty-free basis to BMJ Publishing Group Ltd ("BMJ") its licensees and where the relevant Journal is co-owned by BMJ to the co-owners of the Journal, to publish the Work in this journal and any other BMJ products and to exploit all rights, as set out in our licence.*

*The Submitting Author accepts and understands that any supply made under these terms is made by BMJ to the Submitting Author unless you are acting as an employee on behalf of your employer or a postgraduate student of an affiliated institution which is paying any applicable article publishing charge ("APC") for Open Access articles. Where the Submitting Author wishes to make the Work available on an Open Access basis (and intends to pay the relevant APC), the terms of reuse of such Open Access shall be governed by a Creative Commons licence – details of these licences and which Creative Commons licence will apply to this Work are set out in our licence referred to above.*

*Other than as permitted in any relevant BMJ Author's Self Archiving Policies, I confirm this Work has not been accepted for publication elsewhere, is not being considered for publication elsewhere and does not duplicate material already published. I confirm all authors consent to publication of this Work and authorise the granting of this licence.*

For peer review only

# Development and validation of a multimorbidity risk prediction nomogram among Chinese middle-aged and older adults: a retrospective cohort study

Xiao Zheng[a,c], Benli Xue[b,c], Shujuan Xiao[b,c], Xinru Li[b,c], Yimin Chen[b,c], Lei Shi[b,c], Xiaoyan Liang[a,c], Feng Tian[a,c] *, Chichen Zhang[b,a,c]*

[a] Department of Health Management, Shunde Hospital, Southern Medical University (The First People's Hospital of Shunde, Foshan), Foshan, China

[b] School of Health Management, Southern Medical University, Guangzhou, China

[c] Key Laboratory of Philosophy and Social Sciences of Guangdong Higher Education Institutions for Collaborative Innovation of Health Management Policy and Precision Health Service

*Correspondence to:

Chichen Zhang, zhangchichen@sina.com, School of Health Management, Southern Medical University, Guangzhou, China.

Feng Tian, Department of Health Management, Shunde Hospital, Southern Medical University (The First People's Hospital of Shunde, Foshan), Foshan, China

For peer review only



For peer review only - http://bmjopen.bmj.com/site/about/guidelines.xhtml

**Abstract**

**Objectives:** The aim of this study is to establish a self-simple-to-use nomogram to predict the risk of multimorbidity among middle-aged and older adults.

**Design:** A retrospective cohort study.

**Participants:** We used data from the Chinese Longitudinal Healthy Longevity Survey, including 7735 samples.

**Main outcome measures:** Samples' demographic characteristics, modifiable lifestyles, and depression were collected. Cox proportional hazard models and nomogram model were used to estimate the risk factors of multimorbidity.

**Results:** A total of 3576 (46.2%) participants have multimorbidity. The result showed that age, female (HR 1.23, 95%CI 1.10-1.36), chronic disease (HR 2.59, 95%CI 2.38-2.82), sleep time (HR 0.78, 95%CI 0.72-0.85), regular PA (HR 0.88, 95%CI 0.81-0.95), drinking (HR 1.27 95%CI1.16-1.39), smoking (HR 1.40, 95%CI 1.26-1.53), BMI (HR 1.04, 95%CI 1.03-1.05) and depression scores (HR 1.02, 95%CI 1.01-1.03) were associated with multimorbidity. The C-index of nomogram models for derivation and validation sets were 0.70 (95%CI 0.69-0.71, $P$=0.006) and 0.71 (95%CI 0.70-0.73, $P$=0.008), respectively.

**Conclusions:** We have crafted a user-friendly nomogram model for predicting multimorbidity risk among middle-aged and older adults. This model integrates readily available and routinely assessed risk factors, enabling the early identification of high-risk individuals and offering tailored preventive and intervention strategies.

**Keywords**



For peer review only - http://bmjopen.bmj.com/site/about/guidelines.xhtml

Multimorbidity, Health risk appraisal, behaviors, Nomogram model, Health management, Longitudinal study

**STRENGTHS AND LIMITATIONS OF THIS STUDY**

1. The model was constructed based on behavioral and household-level risk factors.

2. We have used a culling method to deal with the missing values, this may lead to sample bias and the extrapolation of the model needs to be careful.

3. We could not compare the performance of the nomogram model with different models.

## Background

Multimorbidity, commonly defined as the co-occurrence of two or more chronic conditions(1, 2), has emerged as a significant public health concern and poses challenges for healthcare systems. Extensive evidence(3) has shown that multimorbidity is associated with an increased risk of mortality(4), reduced quality of life, heightened healthcare utilization, and elevated health costs(5, 6). Thus, the prevention of multimorbidity has become a crucial focus for public health interventions. Consequently, it is essential to understand the prevalence trends of multimorbidity and the contributing factors within populations. This understanding will enable individuals to estimate and modify their personal risk of developing multimorbidity.

Although age is undeniably a well-established risk factor for multimorbidity(7), the multifaceted nature of this phenomenon demands a more comprehensive understanding that goes beyond age-related associations. Existing research has indeed confirmed the



For peer review only - http://bmjopen.bmj.com/site/about/guidelines.xhtml

For peer review only

higher prevalence of multimorbidity among older adults, with systematic reviews reporting rates ranging from 55% to 98% in the elderly population(8). Additionally, demographic factors such as female gender, and lower socioeconomic status have consistently been associated with an increased risk of multimorbidity. However, while these factors provide valuable insights, they represent only a fraction of the complex web of variables contributing to multimorbidity.

Previous studies have explored associations between sociodemographic factors, physical characteristics, and social networks with multimorbidity(9, 10). For example, numerous studies have linked poor sleep quality to an elevated risk of multimorbidity(11-14). Furthermore, the impact of body mass index (BMI) and smoking on multimorbidity prevalence underscores the intricate connections among these factors(15). Additionally, the well-documented relationship between depression and common chronic diseases has been established(16, 17), with longitudinal cohort studies demonstrating bidirectional associations between depression and multimorbidity(18).

Despite the substantial body of evidence regarding the associations among sociodemographic factors, social networks, lifestyle factors, depression, and the risk of developing multimorbidity, there remains a notable gap in the literature. This gap pertains to the absence of a comprehensive multivariable prediction model that integrates all these factors, providing a holistic assessment of multimorbidity risk. Our study seeks to address this gap by developing and validating a novel risk assessment model that encompasses a broad spectrum of variables, including those mentioned



For peer review only - http://bmjopen.bmj.com/site/about/guidelines.xhtml

For peer review only

above. Our aim is to equip individuals with a more accurate and personalized estimate of their risk of developing multimorbidity, contributing to a deeper understanding of this multifaceted health issue.

Wider determinants of health (WDHs) encompass a multitude of social, economic, political, and environmental factors that exert influence on health outcomes across an individual's lifespan. This influential model of health determinants places constitutional factors such as sex, age, and genetics at its core, surrounded by concentric layers that encompass individual lifestyle factors, followed by the broader determinants(19). While the core attributes remain relatively fixed, the determinants become more modifiable as the layers extend outward. Existing research has identified that individual lifestyle factors significantly contribute to multimorbidity among older adults. Chudasama also found that adopting a healthier lifestyle was associated with longer life expectancy for middle-aged adults, regardless of the presence of multimorbidity.(4).

In the context of the WDHs framework, individual behaviors constitute the innermost layer, presenting opportunities for modification, particularly through self-health management(20-22). The accurate assessment of one's risk of multimorbidity and the identification of potential risk factors represent critical initial steps in the journey of self-management. Therefore, the development of a user-friendly tool to assist individuals in estimating their risk of multimorbidity is of paramount significance.

A nomogram is a health risk appraisal model that offers individualized, evidence-based, and highly accurate risk estimation (23, 24). It is easy to use and can facilitate self-management-related decision making. It is a user-friendly tool that facilitates decision-



For peer review only - http://bmjopen.bmj.com/site/about/guidelines.xhtml

making related to self-management. In this study, we have developed the first nomogram for predicting the risk of multimorbidity among middle-aged and older adults.

## Methods

### Study population

We utilized data from the China Health and Retirement Longitudinal Study (CHARLS), a nationally representative survey of Chinese residents aged 45 years and above. The baseline survey was conducted in 2011 using a multistage probability sampling strategy and probability-proportional-to-size (PPS) sampling technique to ensure national representativeness. Follow-up waves were conducted in 2013, 2015, and 2018. Detailed information regarding the purpose, design, sample, and questionnaires used in CHARLS can be found in other studies(6, 25, 26). For this study, participants below the age of 45 and those with missing values in any variables were excluded from the analysis. The selection process is outlined in Additional file-Figure S1.

### Measurements

### Multimorbidity

In this study, multimorbidity was defined as the presence of two or more chronic non-communicable diseases, whether physical or psychological(6, 27). We assessed multimorbidity by examining the presence of 14 specific non-communicable diseases. Physical chronic non-communicable diseases encompassed diagnosed conditions such as hypertension, dyslipidemia, diabetes, cancer, chronic lung disease, liver disease,



For peer review only - http://bmjopen.bmj.com/site/about/guidelines.xhtml

heart disease, stroke, kidney disease, digestive disease, asthma, and arthritis. Psychological chronic non-communicable diseases included diagnosed emotional, nervous, or psychiatric problems, as well as memory-related diseases (all diseases were self-reported and diagnosed chronic conditions). To identify individuals with multimorbidity, we calculated the number of chronic diseases present for each participant. The outcome was the time to multimorbidity.

**The modifiable lifestyles**

This study included four well-known healthy lifestyle factors(4): physical activity (PA), smoking, alcohol consumption and diet behavior. Besides, sleep and social activity were included in this study.

The physical activity questionnaire used in CHARLS closely resembled the short version of the International Physical Activity Questionnaire (IPAQ)(28). However, some differences existed between CHARLS and IPAQ, such as assessing PA for a "usual week" instead of "the last 7 days" and lacking information on sedentariness. Additionally, instead of continuous values, four discrete time durations ("< 30 minutes" "≥30 minutes" "< 4 hours" and "≥ 4 hours") were collected(29). We calculated the median score for each intensity level and summed the number of different intensity levels using the metabolic equivalent (MET) as a reference. The weight of each intensity level was derived from the IPAQ scoring protocol. Detailed information on PA and its calculation process can be found in the study by Li(26). The total weekly PA (MET-minutes/week) was calculated by multiplying the frequency, duration, and MET values. According to IPAQ, a minimum total PA of at least 600 MET-



For peer review only

For peer review only - http://bmjopen.bmj.com/site/about/guidelines.xhtml

minutes/week was defined as regular PA, while <600 MET-minutes/week indicated a lack of regular PA.

Smoking was categorized as No (not current smoker) and Yes (current smoker) at the time of assessment. Alcohol consumption status was divided into 2 groups: No (Did not drink in the past 12 months or drinking frequency is less than weekly) and Yes (others). Regular eating behavior was determined based on the frequency of meals per day, with having three meals on time considered as regular eating.

Based on studies conducted in developed countries, respondents' total sleep duration was classified into five categories: <6 hours, 6 to <7 hours, 7 to <8 hours, 8 to <9 hours, and ≥9 hours(30, 31). According to the Healthy China initiative (2019-2030), the length of night-time sleep ≥ 7h was defined as enough sleep in this study (32). Social activity was categorized as "No" and "Yes" based on engagement in social activities within the past 12 months.

**Demographic characteristics**

Demographic characteristics included age, sex (male and female), marital status (others and married), residency (others and rural), education (primary education and below, secondary education and above), and BMI scores.

The covariates, including demographic characteristics and modifiable lifestyles factors were gathered by baseline questionnaire.

**Depression**

Depression was assessed using the Chinese version of the Center for Epidemiological Studies Depression scale (CES-D-10)(33). The CES-D-10 contains of 10 items with 4



For peer review only - http://bmjopen.bmj.com/site/about/guidelines.xhtml

response options: rare, some days (1–2 days), occasionally (3–4 days) and most of the time (5–7 days)(25). The scales for each of the 10 items were adjusted to 0, 1, 2 and 3, resulting in a CES-D-10 score ranging from 0 to 30, with higher scores indicating more negative feelings during the past week(34, 35).

**Statistical analysis**

The participants were randomly divided into a derivation set and a validation set at a ratio of 7:3. Participant characteristics, such as age and BMI, were summarized as mean ± SD and counts with proportions for categorical features. Cox proportional hazard models were used to estimate the associations between modifiable lifestyles (including physical activity, smoking, alcohol consumption, and diet behavior), depression, and other identified risk factors with the development of multimorbidity in middle-aged and older adults. Hazard ratios (HR) and 95% confidence intervals (95% CI) were reported for the total population. Factors with a significant level of less than 0.05 in the univariable regression model were entered into the multivariable Cox proportional hazard model for adjustment.

A nomogram was developed based on the results of the multivariable cox proportional hazard model in the derivation set. The nomogram assigns risk points to each variable by proportionally converting regression coefficients to a 0–100-point scale. The variable with the highest absolute value of the β coefficient is assigned 100 points. The risk points for other variables are calculated based on the ratio of risk points to the $\beta$ coefficient of the highest variable. A prognostic index (PI) was calculated by summing the risk points corresponding to each weighted covariate. The nomogram was validated



For peer review only - http://bmjopen.bmj.com/site/about/guidelines.xhtml

using the concordance index (C-index) calculated through 1000-fold bootstrap resampling to reduce overfit bias. The developed nomogram was then applied to the validation set. Model performance was further evaluated using a calibration curve, which superimposes both datasets for visual comparison of discrimination. All analyses were performed using R, version 3.0, $P<0.05$ was considered to indicate statistical significance.

**Patient and public involvement:** The public were not involved in the design, or conduct, or reporting, or dissemination plans of this research.

## Results

### Baseline characteristics

A total of 7,735 participants were included in this study, with 5,449 individuals in the derivation set and 2,286 in the validation set. The baseline characteristics of the study sample are presented in Table 1. The average age of participants in both datasets was 59.0±9.2 years, and in 2011, 3,726 individuals (48.2%) had at least one chronic disease.

**Table 1 Characteristics of the sample on the baseline-2011**

| Characteristics | *N (%) /S±$\bar{X}$* | | |
|---|---|---|---|
| | **All** | **Derivation Set** | **Validation Set** |
| **Age (y)** | | | |
| <60 | 4387(56.7) | 3039(55.8) | 1348(59.0) |
| ≥60 | 3348(43.3) | 2410(44.2) | 938(41.0) |
| **Sex** | | | |
| Male | 3744(48.4) | 2684(49.3) | 1060(46.4) |
| Female | 3991(51.6) | 2765(50.7) | 1226(53.6) |



For peer review only - http://bmjopen.bmj.com/site/about/guidelines.xhtml

| | | | |
|---|---|---|---|
| **Hukou** | | | |
| Others | 6423(83.0) | 4504(82.7) | 1919(83.9) |
| Rural | 1312(17.0) | 945(17.3) | 367(16.1) |
| **Education** | | | |
| Primary education and below | 5296(68.5) | 3720(68.3) | 1576(68.9) |
| Secondary education and above | 2439(31.5) | 1729(31.7) | 710(31.1) |
| **Martial** | | | |
| Others | 1304(16.9) | 910(16.7) | 394(17.2) |
| Married | 6431(83.1) | 4539(83.3) | 1892(82.8) |
| **Chronic Disease** | | | |
| No | 4009(51.8) | 2817(51.7) | 1192(52.1) |
| Yes | 3726(48.2) | 2632(48.3) | 1094(47.9) |
| **Sleep (h)** | | | |
| <7 | 3803(49.2) | 2701(49.6) | 1102(48.2) |
| ≥7 | 3932(50.8) | 2748(50.4) | 1184(51.8) |
| **Social Activity** | | | |
| No | 3864(50.0) | 2747(50.4) | 1117(48.9) |
| Yes | 3871(50.0) | 2702(49.6) | 1169(51.1) |
| **Regular PA** | | | |
| No | 3422(44.2) | 2407(44.2) | 1015(44.4) |
| Yes | 4313(55.8) | 3042(55.8) | 1271(55.6) |
| **Regular Eating** | | | |
| No | 1009(13.0) | 707(13.0) | 302(13.2) |
| Yes | 6726(87.0) | 4742(87.0) | 1984(86.8) |
| **Smoke** | | | |
| No | 3379(43.7) | 2390(43.9) | 989(43.3) |
| Yes | 4356(56.3) | 3059(56.1) | 1297(56.7) |
| **Drink** | | | |



For peer review only

For peer review only - http://bmjopen.bmj.com/site/about/guidelines.xhtml

| | | | |
|---|---|---|---|
| No | 3354(43.4) | 2387(43.8) | 967(42.3) |
| Yes | 4381(56.6) | 3062(56.2) | 1319(57.7) |
| BMI | 23.32±3.69 | 23.31±3.72 | 23.35±3.62 |
| **Depression** | 9.13±4.57 | 9.10±4.56 | 9.21±4.60 |

## Prevalence of multimorbidity

In 2018, a total of 3,576 participants (46.2%) were found to have multimorbidity. Among these individuals, the prevalence of multimorbidity was higher among older adults compared to those under the age of 60 (51.0% vs. 42.6%). Additionally, females exhibited a higher prevalence of multimorbidity compared to males (48.5% vs. 43.8%). Moreover, married individuals (45.7% vs. 48.6%) and those with higher education (42.1% vs. 48.1%) had a lower prevalence of multimorbidity compared to others (Figure 1).

**Figure 1. Prevalence of multimorbidity in the population**

## Predictor factors of multimorbidity

The results of the univariable Cox proportional hazard model for the entire dataset (Additional file-Table S1) indicated that several factors were significantly associated with an increased risk of multimorbidity. These factors included older age, being female (HR 1.16, 95% CI 1.08-1.24), having existing chronic disease (HR 2.85, 95%CI 2.66-3.06), and higher depression scores (HR 1.04, 95%CI 1.03-1.05). Conversely, a higher level of education (HR 0.83, 95%CI 0.77-0.90), marital status (HR 0.91, 95%CI 0.83-0.99), higher BMI scores (HR 1.04, 95%CI 1.03-1.05), smoking (HR 1.46, 95%CI 1.36-1.56), drinking (1.46, 95%CI 1.36-1.56), getting at least 7 hours of sleep (HR 0.70,



For peer review only - http://bmjopen.bmj.com/site/about/guidelines.xhtml

95%CI 0.66-0.75), engaging in regular PA (HR 0.83, 95%CI 0.77-0.88) were associated with a decreased risk of multimorbidity.

The multivariable Cox proportional hazard models incorporated eleven significant variables identified through univariate analysis. Variable selection was performed using the backward selection method. In comparison to individuals under the age of 50, those in the age groups of 50-59 (HR 1.18, 95%CI 1.04-1.33), 60-69 (HR 1.44, 95%CI 1.27-1.64), and 70-79 (HR 1.23, 95%CI 1.04-1.45) exhibit a higher risk of multimorbidity. Furthermore, being female (HR 1.23, 95%CI 1.10-1.36), having a history of chronic disease (HR 2.59, 95%CI 2.38-2.82), getting at least 7 hours of sleep (HR 0.78, 95%CI 0.72-0.85), engaging in regular PA (HR 0.88, 95%CI 0.81-0.95), drinking (HR 1.27 95%CI 1.16-1.39), smoking (HR 1.40, 95%CI 1.26-1.53), higher BMI (HR 1.04, 95%CI 1.03-1.05) and increased depression scores (HR 1.02, 95%CI 1.01-1.03) were all found to be associated with multimorbidity in derivation set (Table 2).

**Table 2 Factors associated with the risk of multimorbidity (multivariable cox proportional hazard model)**

| Factors | Derivation Set | | | Validation Set | | |
|---|---|---|---|---|---|---|
| | *HR* | *95%CI* | *P* | *HR* | *95%CI* | *P* |
| Age(<50) | | | | | | |
| Age(50-59) | 1.18 | 1.04-1.33 | 0.008 | 1.20 | 1.01-1.43 | 0.049 |
| Age(60-69) | 1.44 | 1.27-1.64 | <0.001 | 1.26 | 1.04-1.52 | 0.018 |
| Age(70-79) | 1.23 | 1.04-1.45 | 0.014 | 1.24 | 0.99-1.56 | 0.060 |
| Age(>=80) | 0.92 | 0.70-1.23 | 0.579 | 1.01 | 0.66-1.55 | 0.964 |
| Sex (Male) | | | | | | |
| Female | 1.23 | 1.10-1.36 | <0.001 | 1.43 | 1.22-1.67 | <0.001 |



For peer review only - http://bmjopen.bmj.com/site/about/guidelines.xhtml

For peer review only

| | | | | | | |
|---|---|---|---|---|---|---|
| Chronic disease (No) | | | | | | |
| Yes | 2.59 | 2.38-2.82 | <0.001 | 2.70 | 2.37-3.06 | <0.001 |
| Sleep (<7h) | | | | | | |
| >=7h | 0.78 | 0.72-0.85 | <0.001 | 0.74 | 0.65-0.83 | <0.001 |
| Regular PA (No) | | | | | | |
| Yes | 0.88 | 0.81-0.95 | 0.001 | 0.88 | 0.77-0.99 | 0.039 |
| Drink (No) | | | | | | |
| Yes | 1.27 | 1.16-1.39 | <0.001 | 1.35 | 1.17-1.56 | <0.001 |
| Smoke (No) | | | | | | |
| Yes | 1.40 | 1.26-1.53 | <0.001 | 1.57 | 1.34-1.84 | <0.001 |
| BMI | 1.04 | 1.03-1.05 | <0.001 | 1.02 | 1.01-1.03 | 0.001 |
| Depression | 1.02 | 1.01-1.03 | <0.001 | 1.02 | 1.01-1.04 | 0.001 |

**Development and validation of an multimorbidity -predicting nomogram**

The PI was calculated based on the hazard ratio associated with the identified risk factors for multimorbidity. The nomogram was constructed using these results, with the BMI variable assigned a total scale of 100 and a range of 5 to 50. The risk score for BMI was determined to be 2.2. The risk scores for the other risk factors of multimorbidity were calculated proportionally to the β coefficient of BMI. So the PI=(0.3*I[age-45])+(14.3*I[female]) +(56.9*I[chronic disease])+(14.8*I[1-sleep>=7h)+(6.4*I[1-regular PA])+(13.7*I[drink])+(19.1*I[smoke])+(2.2*BMI)+(1.4*depression), where I[ ]denotes the indicator function equal to 1 if the condition in parenthesis is met and 0 otherwise, except age. Based upon these findings, a nomogram was configured (Figure 2).

For peer review only



For peer review only - http://bmjopen.bmj.com/site/about/guidelines.xhtml

**Figure 2. The nomogram for multimorbidity risk prediction. Note: Draw a line perpendicular from the corresponding axis of each risk factor until it reaches the line labeled "POINTS". Sum up the number of points for all risk factors then draw a line descending from the labeled "TOTAL POINTS" until it intercepts each of the survival axes to determine 2-, 4-, and 7-year survival probabilities, multimorbidity probability=1- survival probability.**

The resulting nomogram was internally validated using the bootstrap validation method, and it demonstrated good accuracy in estimating the risk of multimorbidity, with a bootstrap-corrected C-index of 0.70 (95%CI 0.69-0.71, *P*=0.006) in the derivation set. Calibration plots also indicated good agreement between the risk estimation by the nomogram and the diagnosis of doctors, as depicted in Figure 2. When the estimates from the derivation set were applied to the validation set, a similar bootstrap-corrected C-index of 0.71 (95%CI 0.70-0.73, *P*=0.008) was obtained, along with a well-calibrated risk estimation curve (Figure 3).

**Figure 3. The predictive performance of the nomogram in estimating the risk of multimorbidity**

## Discussion

In this study, we observed a prevalence of multimorbidity of 46.2% among middle-aged and older adults. The prevalence of multimorbidity was higher among individuals with one chronic disease (62.8%) compared to those without chronic diseases (30.8%) at baseline. This result is consistent with the probability range of multimorbidity



For peer review only - http://bmjopen.bmj.com/site/about/guidelines.xhtml

reported in a systematic study of the elderly(8). Although there were some differences in the estimation of multimorbidity compared to other studies, such as the number of included chronic diseases(36, 37), and the methods used to collect information(38). Our results confirmed the significant impact of multimorbidity on middle-aged and older adults, particularly among those with one chronic disease.

Our findings provide sufficient evidence for the association between older age and multimorbidity, which is consistent with similar trends observed in countries such as Singapore(39), Ireland, and Scotland(27). The prevalence of multimorbidity among females is higher than males. Additionally, we found a higher prevalence of multimorbidity among females compared to males, which is supported by studies from various countries indicating that older men have a lower risk of multimorbidity than their female counterparts(27, 38). However, a study by Lian found that the onset of multimorbidity occurs at an earlier age in males than in females(39). This is also a new idea to understand the sex-differ in multimorbidity, but our study only included middle-aged and older adults aged over 45 years, we cannot evaluate this result of Lian. Future research could explore the prevalence and different combinations of chronic conditions in people with multimorbidity across various age and sex groups.

Existing studies have established a link between short sleep duration and multimorbidity. Experimental evidence confirms the deleterious effects of sleep deprivation on endocrine, immune, neurovitality and inflammatory pathways(40). For instance, Maria Ruiz-Castell found an association between short sleep duration and the number of chronic conditions(41). Helbig also observed a significant positive



For peer review only - http://bmjopen.bmj.com/site/about/guidelines.xhtml

relationship between short sleep duration and multimorbidity among women(42). High-risk lifestyles, such as smoking, excessive alcohol consumption, poor diet, physical inactivity, and unhealthy body shape, have also been confirmed as contributing factors to multimorbidity(43). Smoking and excessive alcohol consumption remain leading risk factors for early death and disability globally(44, 45). In our study, we found that smoking and drinking increased the risk of multimorbidity, with the highest risk index among all unhealthy behaviors. The expansion of tobacco and alcohol control measures remains a significant public health priority worldwide. Mika conducted an observational study using data from two Finnish cohort studies comprising 614,014 adults, and the results showed that obesity is a significant factor in multimorbidity(46). Similarly, our study found that higher BMI was associated with an increased risk of multimorbidity among middle-aged and older adults. There exists a bidirectional association between depression and multimorbidity(16, 47). Depression increases the risk of multimorbidity, while having multimorbidity also raises the risk of depression. Our study found the risk of multimorbidity for middle-aged and older adults with higher depression scores.

Based on our results, we developed a user-friendly nomogram model for predicting the risk of multimorbidity. One of the most appealing aspects of our nomogram model is its home applicability and ease of use by individuals. For example, a 50-year-old married male with primary education, one chronic disease, a history of smoking and excessive alcohol consumption, irregular physical activity, 8 hours of sleep, a depression score of 16, and a BMI of 24.9 would have a total risk score of 219.3 points.



For peer review only - http://bmjopen.bmj.com/site/about/guidelines.xhtml

This corresponds to a 2-, 4-, and 7-year probability of multimorbidity of 32%, 71%, and <10%, respectively. Based on the calculated results, individuals can develop self-management strategies to reduce their risk of multimorbidity.

**Limitation**

Our study had several limitations. First, the model was constructed based on behavioral and household-level risk factors, limiting its applicability to clinical prediction. Additionally, in this study, there is a large proportion of missing data for some important variables, so we have used a culling method to deal with the missing values. As a result, this may lead to sample bias and the extrapolation of the model needs to be careful. We also need to validate the model using external data.

## Conclusions

This study confirms the severity of multimorbidity among middle-aged and older adults, particularly among those who already have one chronic disease. Age showed a significant correlation with multimorbidity, and the prevalence of multimorbidity was higher in females compared to males. In addition, insufficient sleep, smoking, drinking, obesity, and depressive symptoms were also associated with multimorbidity. Based on these findings, we developed a user-friendly nomogram model to predict the risk of multimorbidity in middle-aged and older adults. Our research not only builds upon the existing body of knowledge but also introduces a novel and comprehensive approach to assessing multimorbidity risk, which is of significant clinical and public health relevance. The multivariable prediction model provides valuable tools for healthcare



For peer review only - http://bmjopen.bmj.com/site/about/guidelines.xhtml

professionals to manage multimorbidity.

## Ethics statements

### Patient consent for publication

Not applicable.

### Ethics approval

The Biomedical Ethics Review Committee of Peking University approved CHARLS, and all participants were required to provide written informed consent. The ethical approval number was IRB00001052-11015.

## Supplementary materials

Additional file.

## Footnotes

**Contributors:** ZCC and TF conceived the study. ZX and ZCC were major contributors in writing the manuscript. XBL, XSJ, LXR, analyzed and interpreted the patient data. CYM SL and LXY edited and contributed content to the final draft. All authors read and approved the final manuscript.

**Funding:** This study was funded by the Guangdong Basic and Applied Basic Research Foundation (No.2022A1515110295, No.2022A1515011591), National Nature Science Foundation of China (No.72274091), China Postdoctoral Science Foundation (No. 2022M721539), Guangdong Philosophy and Social Science Foundation (No.



For peer review only - http://bmjopen.bmj.com/site/about/guidelines.xhtml

GD23CGL06).

**Competing interests:** The authors declare that they have no competing interests.

**Availability of data and materials:** The datasets analysed during the current study are available from the corresponding author on reasonable request.

## References

1. Fortin M, Bravo G, Hudon C, Vanasse A, Lapointe L. Prevalence of multimorbidity among adults seen in family practice. Ann Fam Med. 2005;3(3):223-8.

2. Jiao DD, Watanabe K, Sawada Y, Tanaka E, Watanabe T, Tomisaki E, et al. Multimorbidity and functional limitation: the role of social relationships. Arch Gerontol Geriat. 2021;92.

3. Nicholson K, Griffith LE, Sohel N, Raina P. Examining early and late onset of multimorbidity in the Canadian Longitudinal Study on Aging. J Am Geriatr Soc. 2021.

4. Chudasama YV, Khunti K, Gillies CL, Dhalwani NN, Davies MJ, Yates T, et al. Healthy lifestyle and life expectancy in people with multimorbidity in the UK Biobank: A longitudinal cohort study. PLoS Med. 2020;17(9):e1003332.

5. Ding D, Lawson KD, Kolbe-Alexander TL, Finkelstein EA, Katzmarzyk PT, van Mechelen W, et al. The economic burden of physical inactivity: a global analysis of major non-communicable diseases. Lancet. 2016;388(10051):1311-24.

6. Zhao Y, Atun R, Oldenburg B, McPake B, Tang S, Mercer SW, et al. Physical multimorbidity, health service use, and catastrophic health expenditure by socioeconomic groups in China: an analysis of population-based panel data. The Lancet

For peer review only



For peer review only - http://bmjopen.bmj.com/site/about/guidelines.xhtml

Global Health. 2020;8(6):e840-e9.

7. Zheng X, Cui Y, Xue Y, Shi L, Guo Y, Dong F, Zhang C. Adverse childhood experiences in depression and the mediating role of multimorbidity in mid-late life: A nationwide longitudinal study. J Affect Disord. 2022 Mar 15;301:217-224.

8. Marengoni A, Angleman S, Melis R, Mangialasche F, Karp A, Garmen A, et al. Aging with multimorbidity: A systematic review of the literature. Ageing Res Rev. 2011;10(4):430-9.

9. Xue B, Xue Y, Dong F, Zheng X, Shi L, Xiao S, Zhang J, Ou W, Wang Q, Zhang C. The impact of socioeconomic status and sleep quality on the prevalence of multimorbidity in older adults. Front Public Health. 2022 Sep 26;10:959700.

10. Olaya B, Domenech-Abella J, Moneta MV, Lara E, Caballero FF, Rico-Uribed LA, et al. All-cause mortality and multimorbidity in older adults: The role of social support and loneliness. Exp Gerontol. 2017;99:120-6.

11. Bertuol C, Tozetto WR, Streb AR, Del Duca GF. Combined relationship of physical inactivity and sedentary behaviour with the prevalence of noncommunicable chronic diseases: data from 52,675 Brazilian adults and elderly. Eur J Sport Sci. 2021.

12. Lindstrom J, Uusitupa M. Lifestyle intervention, diabetes, and cardiovascular disease. Lancet. 2008;371(9626):1731-3.

13. Elwood P, Galante J, Pickering J, Palmer S, Bayer A, Ben-Shlomo Y, et al. Healthy Lifestyles Reduce the Incidence of Chronic Diseases and Dementia: Evidence from the Caerphilly Cohort Study. Plos One. 2013;8(12).

14. Hu FB, Liu Y, Willett WC. Preventing chronic diseases by promoting healthy diet



For peer review only - http://bmjopen.bmj.com/site/about/guidelines.xhtml

and lifestyle: public policy implications for China. Obes Rev. 2011;12(7):552-9.

15. Fortin M, Haggerty J, Almirall J, Bouhali T, Sasseville M, Lemieux M. Lifestyle factors and multimorbidity: a cross sectional study. BMC Public Health. 2014;14:686.

16. Birk JL, Kronish IM, Moise N, Falzon L, Yoon S, Davidson KW. Depression and Multimorbidity: Considering Temporal Characteristics of the Associations Between Depression and Multiple Chronic Diseases. Health Psychology. 2019;38(9):802-11.

17. Meng L, Chen DM, Yang Y, Zheng Y, Hui RT. Depression increases the risk of hypertension incidence: a meta-analysis of prospective cohort studies. J Hypertens. 2012;30(5):842-51.

18. Qiao YN, Liu SY, Zhang YX, Wu Y, Shen YP, Ke CF. Bidirectional association between depression and multimorbidity in middle-aged and elderly Chinese adults: a longitudinal cohort study. Aging & Mental Health. 2021.

19. Bambra C, Gibson M, Sowden A, Wright K, Whitehead M, Petticrew M. Tackling the wider social determinants of health and health inequalities: evidence from systematic reviews. J Epidemiol Commun H. 2010;64(4):284-91.

20. Chodosh J, Morton SC, Mojica W, Maglione M, Suttorp MJ, Hilton L, et al. Meta-analysis: Chronic disease self-management programs for older adults. Ann Intern Med. 2005;143(6):427-38.

21. Warsi A, Wang PS, LaValley MP, Avorn J, Solomon DH. Self-management education programs in chronic disease - A systematic review and methodological critique of the literature. Arch Intern Med. 2004;164(15):1641-9.

22. Li WY, Chiu FC, Zeng JK, Li YW, Huang SH, Yeh HC, et al. Mobile Health App



For peer review only - http://bmjopen.bmj.com/site/about/guidelines.xhtml

With Social Media to Support Self-Management for Patients With Chronic Kidney Disease: Prospective Randomized Controlled Study. Journal of Medical Internet Research. 2020;22(12).

23. Lei Z, Li J, Wu D, Xia Y, Wang Q, Si A, et al. Nomogram for Preoperative Estimation of Microvascular Invasion Risk in Hepatitis B Virus-Related Hepatocellular Carcinoma Within the Milan Criteria. JAMA Surg. 2016;151(4):356-63.

24. B OH, Gransar H, Callister T, Shaw LJ, Schulman-Marcus J, Stuijfzand WJ, et al. Development and Validation of a Simple-to-Use Nomogram for Predicting 5-, 10-, and 15-Year Survival in Asymptomatic Adults Undergoing Coronary Artery Calcium Scoring. JACC Cardiovasc Imaging. 2018;11(3):450-8.

25. Jing R, Xu T, Rong H, Lai X, Fang H. Longitudinal Association Between Sleep Duration and Depressive Symptoms in Chinese Elderly. Nat Sci Sleep. 2020;12:737-47.

26. Li X, Zhang W, Zhang W, Tao K, Ni W, Wang K, et al. Level of physical activity among middle-aged and older Chinese people: evidence from the China health and retirement longitudinal study. BMC Public Health. 2020;20(1):1682.

27. Barnett K, Mercer SW, Norbury M, Watt G, Wyke S, Guthrie B. Epidemiology of multimorbidity and implications for health care, research, and medical education: a cross-sectional study. Lancet. 2012;380(9836):37-43.

28. Craig CL, Marshall AL, Sjostrom M, Bauman AE, Booth ML, Ainsworth BE, et al. International physical activity questionnaire: 12-country reliability and validity. Med Sci Sports Exerc. 2003;35(8):1381-95.



For peer review only - http://bmjopen.bmj.com/site/about/guidelines.xhtml

29. Zhao Y, Hu Y, Smith JP, Strauss J, Yang G. Cohort profile: the China Health and Retirement Longitudinal Study (CHARLS). Int J Epidemiol. 2014;43(1):61-8.

30. Yang L, Yang H, He M, Pan A, Li X, Min X, et al. Longer Sleep Duration and Midday Napping Are Associated with a Higher Risk of CHD Incidence in Middle-Aged and Older Chinese: the Dongfeng-Tongji Cohort Study. Sleep. 2016;39(3):645-52.

31. Paudel M, Taylor BC, Ancoli-Israel S, Blackwell T, Maglione JE, Stone K, et al. Sleep Disturbances and Risk of Depression in Older Men. Sleep. 2013;36(7):1033-40.

32. China TPsRo. Healthy China initiative (2019-2030) 2019 [Available from: http://www.gov.cn/xinwen/2019-07/15/content_5409694.htm.

33. Jin Y, Luo Y, He P. Hypertension, socioeconomic status and depressive symptoms in Chinese middle-aged and older adults: Findings from the China health and retirement longitudinal study. J Affect Disord. 2019;252:237-44.

34. Wang RX, Chen Z, Zhou YJ, Shen LN, Zhang Z, Wu X. Melancholy or mahjong? Diversity, frequency, type, and rural-urban divide of social participation and depression in middle- and old-aged Chinese: A fixed-effects analysis. Soc Sci Med. 2019;238.

35. Chen Z, Ying J, Ingles J, Zhang D, Rajbhandari-Thapa J, Wang R, et al. Gender differential impact of bereavement on health outcomes: evidence from the China Health and Retirement Longitudinal Study, 2011-2015. BMC Psychiatry. 2020;20(1):514.

36. Diederichs C, Berger K, Bartels DB. The Measurement of Multiple Chronic Diseases-A Systematic Review on Existing Multimorbidity Indices. J Gerontol a-Biol. 2011;66(3):301-11.

37. Guisado-Clavero M, Roso-Llorach A, Lopez-Jimenez T, Pons-Vigues M, Foguet-



For peer review only - http://bmjopen.bmj.com/site/about/guidelines.xhtml

Boreu Q, Munoz MA, et al. Multimorbidity patterns in the elderly: a prospective cohort study with cluster analysis. Bmc Geriatrics. 2018;18.

38. Hewitt J, McCormack C, Tay HS, Greig M, Law J, Tay A, et al. Prevalence of multimorbidity and its association with outcomes in older emergency general surgical patients: an observational study. Bmj Open. 2016;6(3).

39. Low LL, Kwan YH, Ko MSM, Yeam CT, Lee VSY, Tan WB, et al. Epidemiologic Characteristics of Multimorbidity and Sociodemographic Factors Associated With Multimorbidity in a Rapidly Aging Asian Country. JAMA Netw Open. 2019;2(11):e1915245.

40. Ferrie JE, Kivimaki M, Akbaraly TN, Singh-Manoux A, Miller MA, Gimeno D, et al. Associations Between Change in Sleep Duration and Inflammation: Findings on C-reactive Protein and Interleukin 6 in the Whitehall II Study. American Journal of Epidemiology. 2013;178(6):956-61.

41. Ruiz-Castell M, Makovski TT, Bocquet V, Stranges S. Sleep duration and multimorbidity in Luxembourg: results from the European Health Examination Survey in Luxembourg, 2013-2015. BMJ Open. 2019;9(8):e026942.

42. Helbig AK, Stockl D, Heier M, Thorand B, Schulz H, Peters A, et al. Relationship between sleep disturbances and multimorbidity among community-dwelling men and women aged 65-93 years: results from the KORA Age Study. Sleep Med. 2017;33:151-9.

43. Han Y, Hu Y, Yu C, Guo Y, Pei P, Yang L, et al. Lifestyle, cardiometabolic disease, and multimorbidity in a prospective Chinese study. Eur Heart J. 2021;42(34):3374-84.



For peer review only - http://bmjopen.bmj.com/site/about/guidelines.xhtml

44. Beaglehole R, Bates C, Youdan B, Bonita R. Nicotine without smoke: fighting the tobacco epidemic with harm reduction. Lancet. 2019;394(10200):718-20.

45. Mohammad KA, Salomon JA. Smoking prevalence and attributable disease burden in 195 countries and territories, 1990-2015: a systematic analysis from the Global Burden of Disease Study 2015 (vol 389, pg 1885, 2015). Lancet. 2017;390(10103):1644-.

46. Kivimaki M, Strandberg T, Pentti J, Nyberg ST, Frank P, Jokela M, et al. Body-mass index and risk of obesity-related complex multimorbidity: an observational multicohort study. Lancet Diabetes Endocrinol. 2022;10(4):253-63.

47. Huang CQ, Dong BR, Lu ZC, Yue JR, Liu QX. Chronic diseases and risk for depression in old age: A meta-analysis of published literature. Ageing Res Rev. 2010;9(2):131-41.



For peer review only - http://bmjopen.bmj.com/site/about/guidelines.xhtml

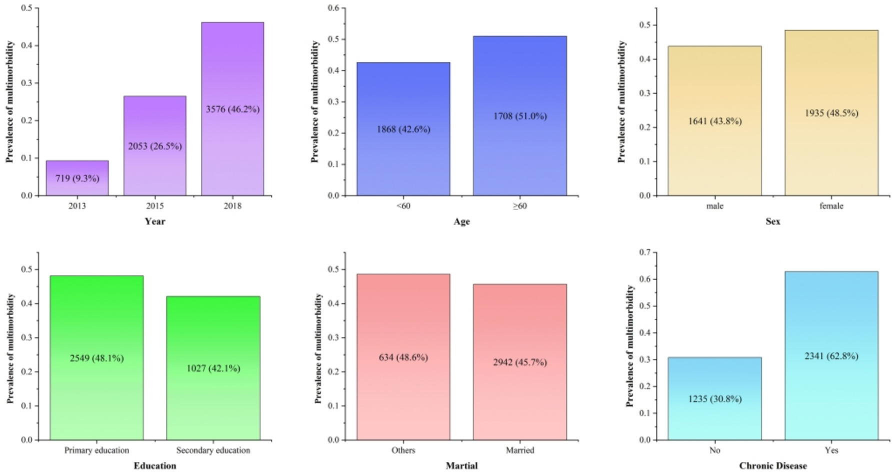


Figure 1. Prevalence of multimorbidity in the population.

338x179mm (300 x 300 DPI)

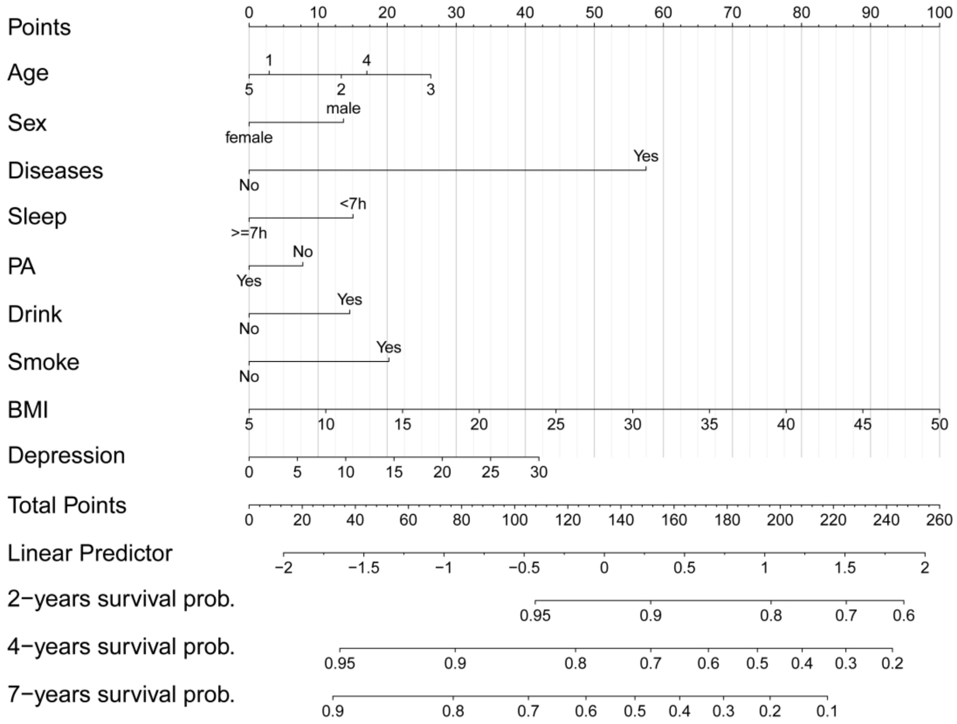


Figure 2. The nomogram for multimorbidity risk prediction. Note: Draw a line perpendicular from the corresponding axis of each risk factor until it reaches the line labeled “POINTS”. Sum up the number of points for all risk factors then draw a line descending from the labeled “TOTAL POINTS” until it intercepts each of the survival axes to determine 2-, 4-, and 7-year survival probabilities, multimorbidity probability=1- survival probability.

728x529mm (118 x 118 DPI)

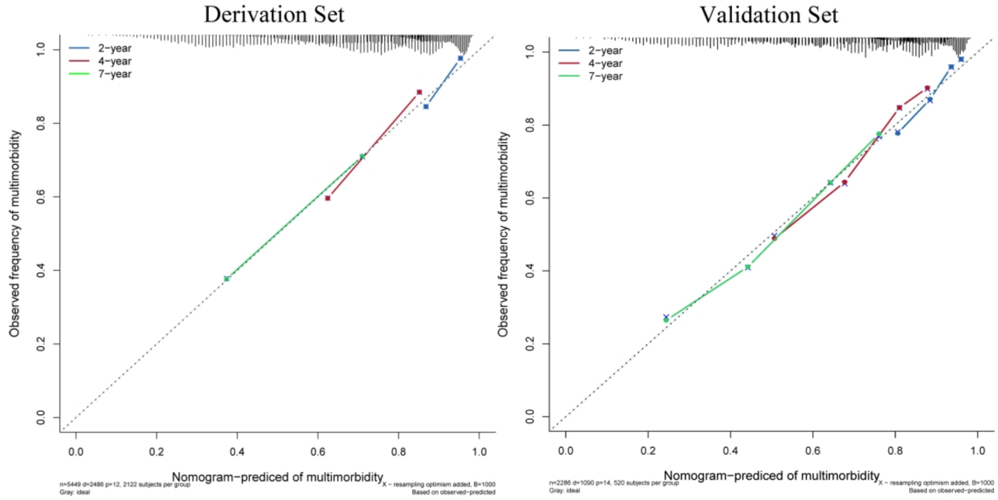


Figure 3. The predictive performance of the nomogram in estimating the risk of multimorbidity

406x210mm (118 x 118 DPI)

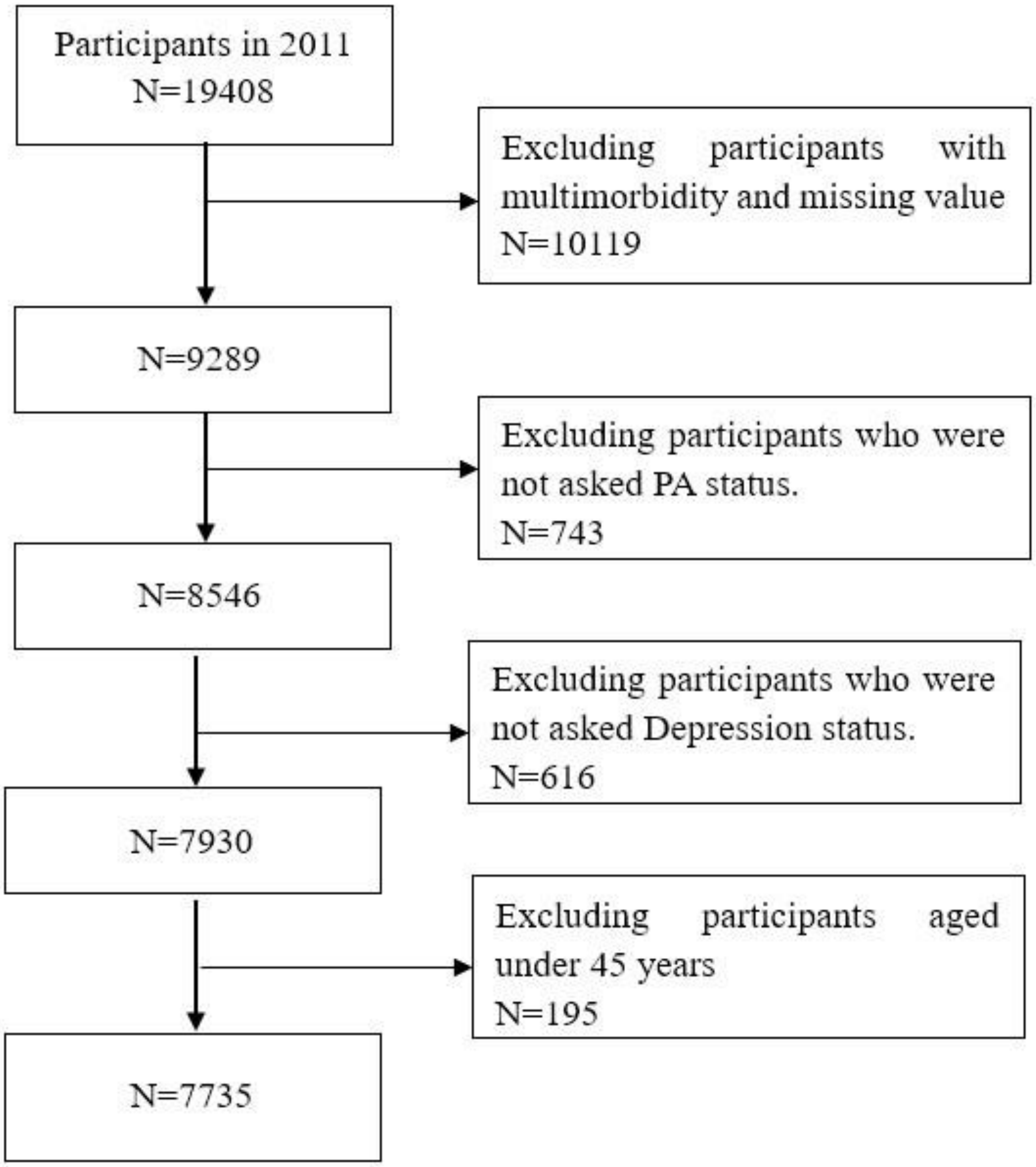


**Figure S1. Flow diagram of Sample selection**

**Table S1 Factors associated with the risk of multimorbidity (univariable cox proportional hazard models, N=7735)**

| Characteristics | | *HR* | *95%CI* | *P* |
|---|---|---|---|---|
| **Age (y)** | <50* | | | |
| | 50-69 | 1.25 | 1.13-1.39 | <0.001 |
| | 60-69 | 1.58 | 1.42-1.74 | <0.001 |
| | 70-79 | 1.41 | 1.25-1.60 | <0.001 |
| | >=80 | 1.12 | 0.90-1.41 | 0.311 |
| **Sex** | Male* | | | |
| | Female | 1.16 | 1.08-1.24 | <0.001 |
| **Hukou** | Rural* | | | |
| | Others | 1.03 | 0.95-1.13 | 0.452 |
| **Education** | Primary* | | | |
| | Secondary | 0.83 | 0.77-0.90 | <0.001 |
| **Marial** | Others* | | | |
| | Married | 0.91 | 0.83-0.99 | 0.027 |
| **Chronic Diseases** | No* | | | |
| | Yes | 2.85 | 2.66-3.06 | <0.001 |
| **Sleep (h)** | <7* | | | |
| | >=7 | 0.70 | 0.66-0.75 | <0.001 |
| **Social Activity** | No* | | | |
| | Yes | 0.96 | 0.9-1.02 | 0.215 |
| **Regular PA** | No* | | | |
| | Yes | 0.83 | 0.77-0.88 | <0.001 |
| **Eating** | No* | | | |
| | Yes | 0.97 | 0.88-1.07 | 0.594 |
| **Smoke** | No* | | | |
| | Yes | 1.46 | 1.36-1.56 | <0.001 |
| **Drink** | No* | | | |
| | Yes | 1.46 | 1.36-1.56 | <0.001 |
| **BMI** | | 1.04 | 1.03-1.05 | <0.001 |
| **Depression** | | 1.04 | 1.03-1.05 | <0.001 |

For peer review only

TRIPOD Checklist: Prediction Model Development and Validation

| Section/Topic | Item | | Checklist Item | Page |
|---|---|---|---|---|
| **Title and abstract** | | | | |
| Title | 1 | D;V | Identify the study as developing and/or validating a multivariable prediction model, the target population, and the outcome to be predicted. | 1 |
| Abstract | 2 | D;V | Provide a summary of objectives, study design, setting, participants, sample size, predictors, outcome, statistical analysis, results, and conclusions. | 2 |
| **Introduction** | | | | |
| Background and objectives | 3a | D;V | Explain the medical context (including whether diagnostic or prognostic) and rationale for developing or validating the multivariable prediction model, including references to existing models. | 3-5 |
| | 3b | D;V | Specify the objectives, including whether the study describes the development or validation of the model or both. | 5-6 |
| **Methods** | | | | |
| Source of data | 4a | D;V | Describe the study design or source of data (e.g., randomized trial, cohort, or registry data), separately for the development and validation data sets, if applicable. | 6 |
| | 4b | D;V | Specify the key study dates, including start of accrual; end of accrual; and, if applicable, end of follow-up. | 6 |
| S1Participants | 5a | D;V | Specify key elements of the study setting (e.g., primary care, secondary care, general population) including number and location of centres. | 6 |
| | 5b | D;V | Describe eligibility criteria for participants. | 6, Figure S1 |
| | 5c | D;V | Give details of treatments received, if relevant. | |
| Outcome | 6a | D;V | Clearly define the outcome that is predicted by the prediction model, including how and when assessed. | 6-7 |
| | 6b | D;V | Report any actions to blind assessment of the outcome to be predicted. | |
| Predictors | 7a | D;V | Clearly define all predictors used in developing or validating the multivariable prediction model, including how and when they were measured. | 7-9 |
| | 7b | D;V | Report any actions to blind assessment of predictors for the outcome and other predictors. | |
| Sample size | 8 | D;V | Explain how the study size was arrived at. | 6, Figure S1 |
| Missing data | 9 | D;V | Describe how missing data were handled (e.g., complete-case analysis, single imputation, multiple imputation) with details of any imputation method. | 6, Figure S1 |
| Statistical analysis methods | 10a | D | Describe how predictors were handled in the analyses. | 7-9 |
| | 10b | D | Specify type of model, all model-building procedures (including any predictor selection), and method for internal validation. | 9-10 |
| | 10c | V | For validation, describe how the predictions were calculated. | 9-10 |
| | 10d | D;V | Specify all measures used to assess model performance and, if relevant, to compare multiple models. | 9-10 |
| | 10e | V | Describe any model updating (e.g., recalibration) arising from the validation, if done. | 9-10 |
| Risk groups | 11 | D;V | Provide details on how risk groups were created, if done. | |
| Development vs. validation | 12 | V | For validation, identify any differences from the development data in setting, eligibility criteria, outcome, and predictors. | 9-10 |
| **Results** | | | | |
| Participants | 13a | D;V | Describe the flow of participants through the study, including the number of participants with and without the outcome and, if applicable, a summary of the follow-up time. A diagram may be helpful. | Figure S1 |
| | 13b | D;V | Describe the characteristics of the participants (basic demographics, clinical features, available predictors), including the number of participants with missing data for predictors and outcome. | Table 1 |
| | 13c | V | For validation, show a comparison with the development data of the distribution of important variables (demographics, predictors and outcome). | Table 1 |
| Model development | 14a | D | Specify the number of participants and outcome events in each analysis. | 11-12 |
| | 14b | D | If done, report the unadjusted association between each candidate predictor and outcome. | 12-13, Table S1 |
| Model specification | 15a | D | Present the full prediction model to allow predictions for individuals (i.e., all regression coefficients, and model intercept or baseline survival at a given time point). | 13-14, Table 2 |
| | 15b | D | Explain how to the use the prediction model. | 14, Figure 2 |
| Model performance | 16 | D;V | Report performance measures (with CIs) for the prediction model. | 15 |
| Model-updating | 17 | V | If done, report the results from any model updating (i.e., model specification, model performance). | |
| **Discussion** | | | | |
| Limitations | 18 | D;V | Discuss any limitations of the study (such as nonrepresentative sample, few events per predictor, missing data). | 18 |
| Interpretation | 19a | V | For validation, discuss the results with reference to performance in the development data, and any other validation data. | 15-17 |
| | 19b | D;V | Give an overall interpretation of the results, considering objectives, limitations, results from similar studies, and other relevant evidence. | 15-17 |
| Implications | 20 | D;V | Discuss the potential clinical use of the model and implications for future research. | 17-19 |

For peer review only

TRIPOD Checklist: Prediction Model Development and Validation

| Other information | | | | |
|---|---|---|---|---|
| Supplementary information | 21 | D;V | Provide information about the availability of supplementary resources, such as study protocol, Web calculator, and data sets. | |
| Funding | 22 | D;V | Give the source of funding and the role of the funders for the present study. | 19 |

*Items relevant only to the development of a prediction model are denoted by D, items relating solely to a validation of a prediction model are denoted by V, and items relating to both are denoted D;V. We recommend using the TRIPOD Checklist in conjunction with the TRIPOD Explanation and Elaboration document.

For peer review only
